# Orphan GPR110 (ADGRF1) targeted by *N*-docosahexaenoylethanolamine in development of neurons and cognitive function

**DOI:** 10.1038/ncomms13123

**Published:** 2016-10-19

**Authors:** Ji-Won Lee, Bill X. Huang, HeungSun Kwon, Md Abdur Rashid, Giorgi Kharebava, Abhishek Desai, Samarjit Patnaik, Juan Marugan, Hee-Yong Kim

**Affiliations:** 1 Laboratory of Molecular Signaling, NIAAA, NIH, 5625 Fishers Lane Room 3N-07, Bethesda, Maryland 20892-9410, USA; 2 Chemistry Division of Preclinical Innovation, National Center for Advancement of Translational Sciences (NCATS), NIH, 9800 Medical Center Dr, Rockville, Maryland 20892, USA

## Abstract

Docosahexaenoic acid (DHA, 22:6n-3) is an omega-3 fatty acid essential for proper brain development. *N*-docosahexaenoylethanolamine (synaptamide), an endogenous metabolite of DHA, potently promotes neurogenesis, neuritogenesis and synaptogenesis; however, the underlying molecular mechanism is not known. Here, we demonstrate orphan G-protein coupled receptor 110 (GPR110, ADGRF1) as the synaptamide receptor, mediating synaptamide-induced bioactivity in a cAMP-dependent manner. Mass spectrometry-based proteomic characterization and cellular fluorescence tracing with chemical analogues of synaptamide reveal specific binding of GPR110 to synaptamide, which triggers cAMP production with low nM potency. Disruption of this binding or GPR110 gene knockout abolishes while GPR110 overexpression enhances synaptamide-induced bioactivity. GPR110 is highly expressed in fetal brains but rapidly decreases after birth. GPR110 knockout mice show significant deficits in object recognition and spatial memory. GPR110 deorphanized as a functional synaptamide receptor provides a novel target for neurodevelopmental control and new insight into mechanisms by which DHA promotes brain development and function.

## Abstract (web summary)

*N*-docosahexaenoylethanolamine (synaptamide) promotes neurogenesis but the molecular mechanism involved is unknown. Here, the authors show that orphan G-protein coupled receptor 110 is the synaptamide receptor, with deletion in mice affecting development of neurons, object recognition and spatial memory.

## Graphical abstract

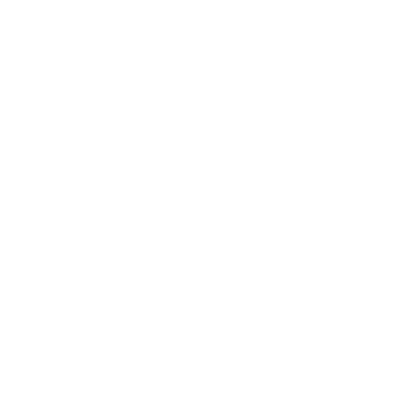

Omega-3 fatty acids are essential nutrients^1^. Docosahexaenoic acid (DHA, 22:6n-3), an omega-3 fatty acid highly enriched in the brain, plays a significant role in brain development, as well as cognitive and memory function^2^,^3^,^4^,^5^, but specific molecular mechanisms responsible for this beneficial effect are not clearly understood. DHA promotes neurite outgrowth, synaptogenesis and synaptic function in cultured hippocampal neurons^6^,^7^, and induces neuronal differentiation of neural stem cells (NSCs) *in vivo* and *in vitro*^8^. Several pre- and postsynaptic proteins involved in neurotransmission and synapse formation are reduced in the DHA-depleted brains^9^, indicating an important role of DHA in maintaining synapse integrity *in vivo*.

*N*-docosahexaenoylethanolamine, an endogenous metabolite of DHA, significantly increases neuritogenesis, synaptogenesis and glutamatergic synaptic activity^10^ and potently induces neurogenic differentiation^11^. The similarity of its structure to a well-known endocannabinoid *N*-arachidonylethanolamine (anandamide), suggests that synaptamide may use the biosynthetic mechanism involving hydrolysis of *N*-acylphosphatidylethanolamine (NAPE) that has been established for anandamide^12^. The significant decreases in synaptamide recently reported in the brain regions of NAPE-phospholipase D (NAPE-PLD) knockout (KO) mice^13^ support such a mechanism via *N*-docosahexaenoylphosphatidylethanolamine (NDPE). On the basis of the synaptogenic property and its endocannabinoid-like amide structure, the term ‘synaptamide' was coined for *N*-docosahexaenoylethanolamine^14^. We used ‘synaptamide' to represent *N*-docosahexaenoylethanolamine since the abbreviation ‘DHEA' is a widely used and accepted term for the steroid, dehydroepiandrosterone. The neurogenic, neuritogenic and synaptogenic bioactivities of DHA and synaptamide are qualitatively similar, but the latter is significantly more potent. The endogenous level of synaptamide in brain is affected by the dietary omega-3 fatty acid intake, which alters brain DHA content^10^,^15^. Furthermore, DHA-derived neurogenic, neuritogenic and synaptogenic bioactivity is considerably enhanced when fatty acid amide hydrolase is inhibited^10^,^11^. These findings suggest that synaptamide is a principal mediator for the observed bioactivity of DHA, most likely involving a specific target receptor.

G-protein coupled receptors (GPCR) are the major regulators of intracellular signalling and are important drug targets^16^,^17^, but many including most of the adhesion GPCRs (aGPCRs) still remain orphan without identified natural ligands^18^. Previously, GPR40 and GPR120 of class A GPCRs were deorphanized as fatty acid receptors that preferentially bind to long chain free fatty acids including DHA^19^,^20^. In this paper, using a chemical and proteomic strategy altogether with molecular and cellular characterization, we identified orphan GPR110 (ADGRF1), a member of the less-known aGPCRs, as the functional synaptamide receptor mediating synaptamide-induced neurite growth and synaptogenesis in cortical neurons and neurogenic differentiation of NSCs. We suggest that synaptamide-activated GPR110 signalling is an important molecular mechanism for the neurodevelopmental actions of omega-3 fatty acids.

## Results

### Synaptamide bioactivity is cAMP-dependent

**Figure 1:**
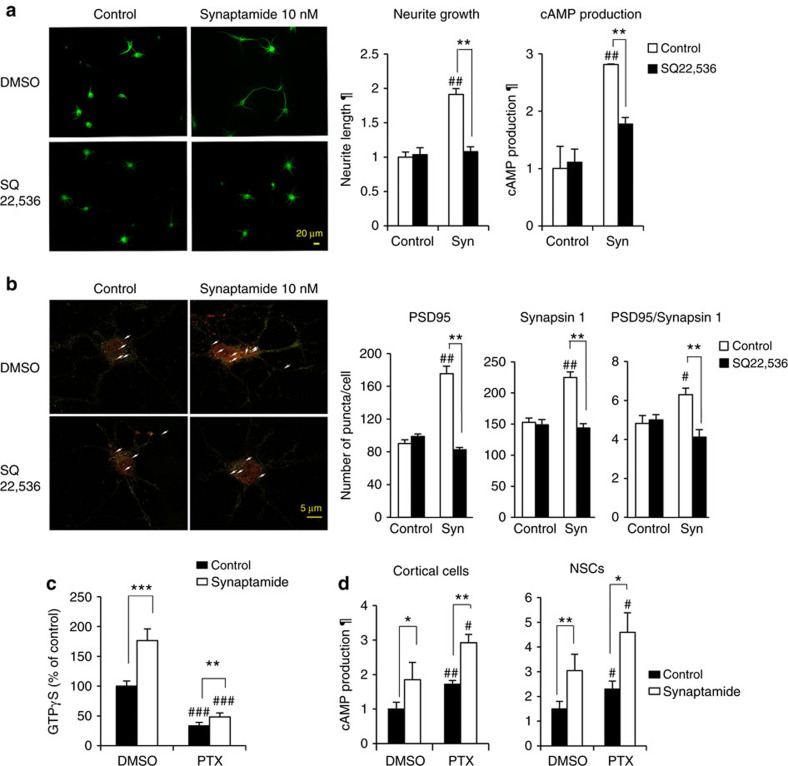
cAMP-dependent induction of neurite outgrowth, synaptogenesis and neurogenesis by synaptamide. In cortical neurons, 10 nM synaptamide (Syn) increased cAMP production, neurite outgrowth (**a**) and synaptogenesis (**b**) while pretreatment with SQ22,536 for 30 min prevented these effects. Neurite outgrowth and synaptogenesis were evaluated after treating neurons from day 1 *in vitro* (DIV1) for 2 or 6 days, respectively, while cAMP production and CREB phosphorylation were determined after stimulating DIV3 neurons with synaptamide for 10 min. Neurons and nuclei were visualized using MAP2 antibody (green) and DAPI (blue). Co-localization analysis of presynaptic marker synapsin1 (red) and postsynaptic marker PSD95 (green) revealed increased synapses (overlapping synapsin1/PSD95 puncta indicated by arrows) by synaptamide treated cortical neurons but not in the presence of SQ22,536 (**b**). For quantification of synaptic protein puncta, 20 cells were scored per well for 3 wells per each group. Synaptamide at 10 nM induced G-protein activation (**c**). The [γ-^35^S] GTP binding was measured after treating NSC membranes with 10 nM synaptamide for 15 min with or without overnight pretreatment with pertussis toxin (PTX, 0.1 μg ml^−1^). While expected increases in cAMP production were observed after PTX pretreatment of cortical cells or NSCs, synaptamide remained capable of increasing cAMP (**d**). Values are expressed as means±s.e.m. of biological triplicates (*n*=3), representing three independent experiments. Statistical analysis was performed using unpaired Student *t*-test. ^#^*P*<0.05, ^##^*P*<0.01, ^###^*P*<0.001, *P<0.05, **P<0.01, ***P<0.001 in comparison with the corresponding control. ¶, relative to control. Scale bars, 20 μm (**a**) and 5 μm (**b**).

Previously, we observed that synaptamide promotes neurite growth and synaptogenesis in hippocampal neurons^10^ and induces neurogenic differentiation^11^ and cyclic adenosine monophosphate (cAMP)/protein kinase A signalling at a low nM range in NSCs^21^. Therefore, we first examined whether the bioactivity of synaptamide depends on cAMP production using two model systems, cortical neurons and NSCs in culture (Fig. 1). Like NSCs^11^, cortical neurons produced synaptamide from DHA (Supplementary Fig. 1). Synaptamide at 10 nM time-dependently increased cAMP production in both cortical neurons and NSCs (Supplementary Fig. 2), and significantly increased CREB phosphorylation and CRE transcriptional activity (Supplementary Fig. 3). Synaptamide promoted neurite growth (Fig. 1a), synaptic protein expression, as well as synaptogenesis evaluated by the overlapping puncta of pre and post-synaptic proteins, synapsin 1 and PSD95 (Synapsin1/PSD95) in cortical neurons (Fig. 1b), and neurogenic differentiation of NSCs (Supplementary Fig. 4). As observed earlier in hippocampal neurons^10^, DHA exhibited similar effect on cortical neurite growth but at a substantially higher concentration than synaptamide while oleic acid (OA) was not effective (Supplementary Fig. 3). Preventing cAMP production with an adenylylcyclase inhibitor SQ22,536 abolished synaptamide-induced cortical neurite outgrowth and synaptogenesis, as well as neurogenic differentiation of NSCs (Fig. 1a,b; Supplementary Fig. 4). G-protein activation was apparent as 10 nM synaptamide increased [γ-^35^S] GTP binding (176.3±8.6%) (Fig. 1c). Despite the significant decrease in [γ-^35^S] GTP binding (33.5±5.7%) produced by overnight pretreatment with pertussis toxin (PTX), 10 nM synaptamide was still able to increase [γ-^35^S] GTP binding (48.4±6.5%). The synaptamide-induced cAMP production was also insensitive to pertussis toxin (Fig. 1d). These results indicate that synaptamide effects are mediated through cAMP signalling and that a non-Gαi-coupled GPCR is involved.

### GPR110 is the synaptamide-binding protein

**Figure 2:**
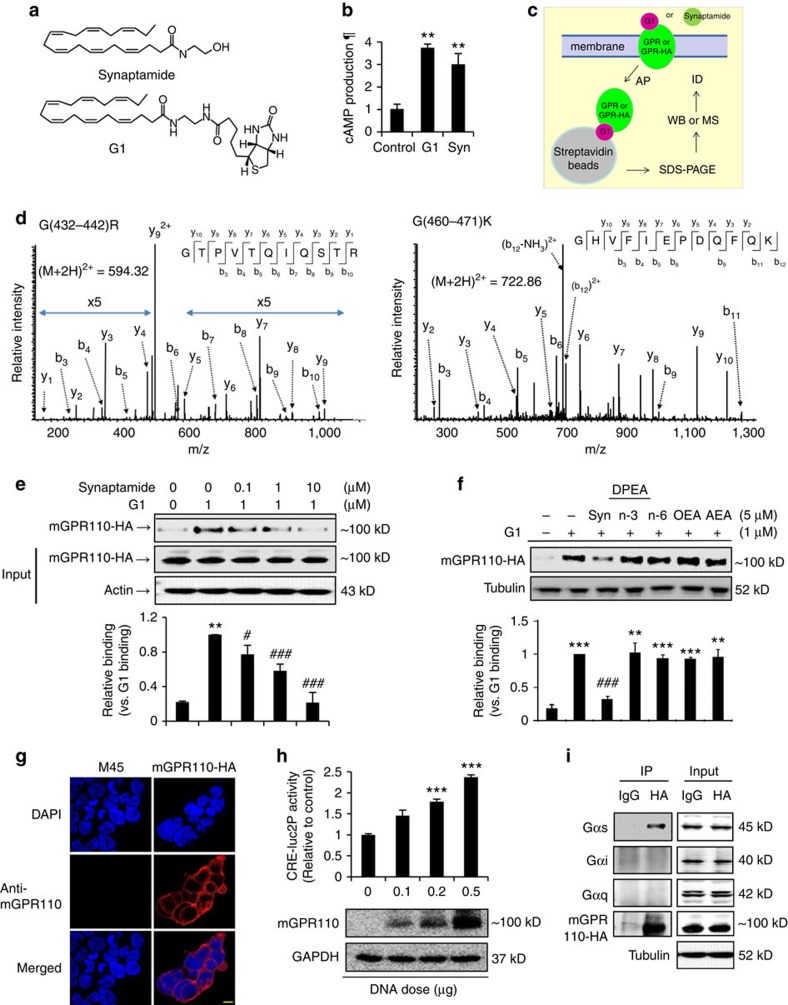
Identification of synaptamide receptor and associated G-protein. Biotinylated synaptamide analogue G1 (**a**) increased cAMP production in DIV3 neurons after 10 min stimulation (**b**). After mouse fetal brains or NSCs were lysed in PBS containing 0.5% Triton X-100, treated with G1, affinity-purified using streptavidin beads and subjected to SDS/PAGE, the entire gel was cut into several bands for tryptic digestion and protein identification (ID) by mass spectrometric analysis (**c**). The MS/MS spectra of GPR110 peptides G(432–442)R and G(460–471)K detected from the gel band in the 100–130 kD molecular weight region are shown (**d**). Binding of G1 to GPR110 was confirmed by the western blot detection of mGPR110-HA expressed in HEK cells at ∼100 kD after streptavidin pull-down while synaptamide dose-dependently decreased the mGPR110-HA recovered after G1-streptavidin pull-down (**e**). Among fatty acid ethanolamides, only synaptamide displaced the G1 binding to mGPR110 (**f**). GPR110 expression in the plasma membrane was detected (**g**). The cAMP response induced by 10 nM synaptamide increased in a GPR110 gene-dose-dependent manner (**h**). GPR110 interaction with Gαs was identified by co-immunoprecipitation of overexpressed mGPR110-HA with endogenous Gαs (**i**). Syn, synaptamide; n3, omega-3; n6, omega-6; DPEA, *N*-docosapentaenoylethanolamine; OEA, *N*-oleoylethanolamine; AEA, *N*-arachidonylethanolamine. Results in **d** and **i** represent two independent experiments. Data in (**b**,**e**,**f**,**h**) are means±s.e.m. of biological triplicates (*n*=3), representing three independent experiments. **P<0.01, ***P<0.001 versus control, and ^*#*^*P*<0.05, ^##^*P*<0.01, ^###^*P*<0.001 versus G1 binding by unpaired Student *t*-test. ¶, relative to control. Scale bar, 5 μm (**g**).

To identify the GPCR that binds to synaptamide, we devised a strategy using a pull down approach coupled to mass spectrometry (MS)^22^. A biotinylated synaptamide analogue was prepared for affinity-purification (AP); G1, (4*Z*,7*Z*,10*Z*,13*Z*,16*Z*,19*Z*)-*N*-(2-(5-((3a*S*,4*S*,6a*R*)-2-oxohexahydro-1*H*-thieno(3,4-d)imidazol-4-yl)pentanamido)ethyl)docosa-4,7,10,13,16,19-hexaenamide (Fig. 2a). This analogue exhibited bioactivity similar to synaptamide, inducing cAMP production (Fig. 2b) and neurite outgrowth (Supplementary Fig. 5). Using G1, we searched for GPCRs according to the scheme illustrated in Fig. 2c. Mouse fetal brains or NSCs were lysed under a mild condition, treated with G1, and incubated with streptavidin beads for affinity-purification. The affinity-purified product was subjected to SDS-PAGE for western blot (WB) or MS analysis for protein identification after in-gel tryptic digestion. In parallel, the lysates were subjected to the same procedure after treatment with biotin instead of G1, and the proteins thus detected were considered as background. This approach allowed us to identify two peptides, G(432–442)R (GTPVTQIQSTR) and G(460–471)K (GHVFIEPDQFQK) (Fig. 2d), that uniquely belong to GPR110 in G1-treated fetal brain and NSC samples (Supplementary Table 1). No other GPCRs were detected using this approach. GPR110 is an orphan receptor that belongs to the adhesion GPCR family and has seven transmembrane (7TM) helices with multiple *N*-linked glycosylation sites^23^,^24^. The molecular mass of GPR110 is about 100 kD, but lower molecular weight isoforms as well as differentially glycosylated forms have been reported^24^.

To confirm that GPR110 is indeed the G1-binding protein, *C*-terminal HA-tagged GPR110 (GPR110-HA) was expressed in HEK cells and subjected to the AP/MS procedure described above. The mouse GPR110-HA (mGPR110-HA) expressed in HEK cells was detected mainly at the apparent molecular weight of ∼100 kD along with a minor band at ∼130 kD using both HA and *N*-terminal targeting mGPR110 antibodies (Supplementary Fig. 6a). After immunoprecipitation (IP) using HA antibody, the IP product was applied to SDS PAGE, and the gel bands at ∼100 and ∼130 kD were cut out and analysed by mass spectrometry. Both *N*- and *C*-terminal, as well as transmembrane region tryptic peptides were detected (Supplementary Fig. 6b, Supplementary Tables 2 and 3), confirming that these protein bands were derived from the full length mGPR110-HA, and therefore, possibly represent different states of posttranslational modification. When the lysate from mGPR110-HA-expressing cells was treated with G1 followed by the streptavidin affinity purification, HA antibody detected the G1-binding protein at ∼100 kD (Fig. 2e,f), confirming binding of G1 to the expressed mGPR110-HA. The competitive binding assayed according to the strategy described above (Fig. 2c) revealed dose-dependent displacement of G1 by synaptamide (Fig. 2e), verifying that both G1 and synaptamide bind to the same receptor, GPR110. Among other fatty acid ethanolamides including anandamide (AEA), n-3 and n-6 docosapentaenoylethanolamide (DPEAn-3, DPEAn-6) or oleoylethanolamide (OEA), only synaptamide significantly displaced G1 binding to GPR110 (Fig. 2f), indicating that synaptamide is a specific ligand to GPR110.

Synaptamide binding activity to human GPR110 (hGPR110) was also examined (Supplementary Fig. 7). The hGPR110-HA expressed in HEK cells showed apparent molecular weight of ∼130 and ∼110 kD (Supplementary Fig. 7a). After deglycosylation using PNGase F, these bands shifted to ∼100 kD. All three bands detected at ∼130, ∼110 and ∼100 kD contained N and *C*-terminal as well as transmembrane region amino acid sequence (Supplementary Fig. 7b–d, Supplementary Tables 4–6), indicating that the bands detected at ∼130 and ∼110 kD are full length GPR110 with differential glycosylation states. Similar to mGPR110, hGPR110 showed G1 binding which was displaced by synaptamide (Supplementary Fig. 7e), demonstrating that synaptamide binds to hGPR110 as was observed with mGPR110.

When GPR110 was overexpressed in HEK293 cells permanently expressing CRE-luc2P as a cAMP sensor^25^, GPR110 expression was detected in the plasma membrane and synaptamide-induced cAMP production increased in a gene-dose dependent manner (Fig. 2g,h). Co-immunoprecipitation (IP) assay indicated that GPR110-HA specifically associates with Gαs (Fig. 2i, Supplementary Fig. 8). When HEK cells expressing GPR110-HA were lysed and subjected to co-IP using HA antibody, only Gαs but not Gαi and Gαq was detected from the pull-down precipitate (Fig. 2i). Reversed IP using Gαs antibody detected GPR110-HA, confirming the association of Gαs with GPR110 (Supplementary Fig. 8a). The interaction between GPR110-HA and GFP-Gαs was confirmed in HEK cells expressing both GPR110-HA and GFP-Gαs (Supplementary Fig. 8b). These data are consistent with the pertussis toxin-insensitive increase in [γ-^35^S] GTP binding and cAMP increases observed after synaptamide treatment (Fig. 1c,d).

### Synaptamide binds to GPR110 in living cells

**Figure 3:**
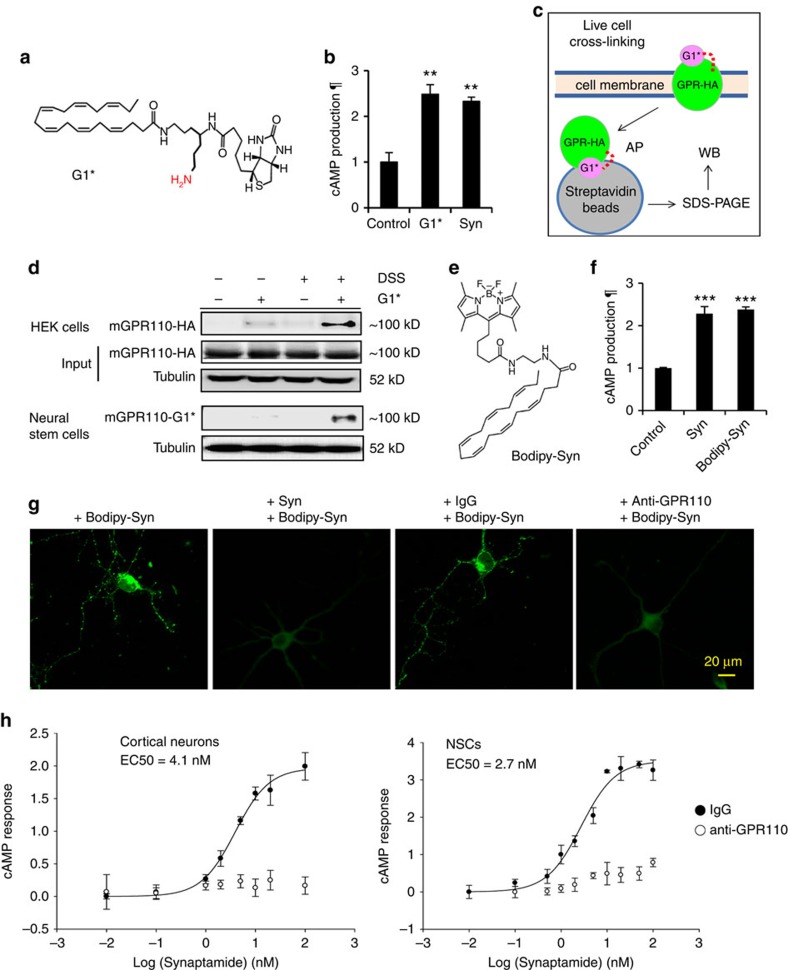
Synaptamide binding to GPR110 in living cells identified by in-cell cross-linking/affinity purification or fluorescence microscopy. Chemical structure of the biotinylated synaptamide analogue (G1*) containing a cross-linkable primary amine group (**a**). G1* and synaptamide at 10 nM similarly induced cAMP production in DIV3 cortical neurons after a 10-min incubation (**b**). Strategy to detect G1* bound-GPR110 in living cells (**c**). The G1*-receptor complex stabilized by DSS cross-linking was detected by anti-HA antibody for GPR110-HA-expressing HEK cells or peroxidase conjugated streptavidin for endogenous GPR110 in NSCs. The control was treated with biotin instead of G1* (**d**). Chemical structure of bodipy-synaptamide (**e**). Similar to synaptamide, bodipy-synaptamide at 10 nM increased cAMP production in DIV3 cortical neurons after a 10-min incubation (**f**). Confocal images of fluorescent endocytic receptor puncta detected after incubating DIV3 cortical neurons with bodipy-synaptamide (100 nM) for 30 min (**g**). Only weak non-specific signal without fluorescent puncta were observed after the pretreatment with anti-GPR110 antibody (0.4 μg ml^−1^) or synaptamide (10 nM) for 30 min, indicating specific binding of synaptamide to GPR110 on the surface of live cortical neurons (**g**). Synaptamide dose-dependently increased cAMP production in cortical neurons and NSCs with EC_50_ in the low nM range (closed circle), which was blocked by 30 min pretreatment with GPR110 antibody at 0.4 μg ml^−1^ (open circle) (**h**). DSS: disuccinimidyl suberate. Data represent two (**d**,**h**), three (**b**,**f**) or six (**g**) independent experiments. Values in (**b**,**f**,**h**) are means±s.e.m. of biological triplicates (*n*=3). **P<0.01, ***P<0.001 versus control by unpaired Student *t*-test. ¶, relative to control. Scale bar, 20 μm (**g**).

To capture the interaction between GPR110 and synaptamide in living cells, in-cell cross-linking^26^ was performed using a cross-linkable analogue G1* ((4*Z*,7*Z*,10*Z*,13*Z*,16*Z*,19*Z*)-*N*-(7-amino-4-(5-((3a*S*,4*S*,6a*R*)-2-oxohexahydro-1*H*-thieno(3,4-*d*)imidazol-4-yl)pentanamido)heptyl)docosa-4,7,10,13,16,19-hexaenamide) that carries a primary amine group (Fig. 3a). This analogue also retained synaptamide-like bioactivity, increasing cAMP (Fig. 3b) and neurite outgrowth (Supplementary Fig. 5). Live HEK cells expressing mGPR110-HA were treated with G1*, cross-linked with a lysine-specific cross-linker disuccinimidylsuberate (DSS), washed extensively and lysed before AP using streptavidin beads as illustrated in Fig. 3c. Subsequent WB analyses using HA-antibody detected GPR110-HA in the G1*-treated, DSS-cross-linked sample, confirming G1* binding to GPR110-HA in live HEK cells (Fig. 3d). The same in-cell cross-linking approach confirmed the binding of endogenous GPR110 to G1* in mouse NSCs (Fig. 3d). In this case, the G1*-bound GPR110 at ∼100 kD was detected by peroxidase conjugated streptavidin, as the GPR110 antibody currently available was not sufficiently sensitive to detect a minute level of cross-linked GPR110. Of note, endogenous expression of mGPR110 in NSCs and cortical neurons was clearly indicated by the protein band at ∼100 kD, particularly after IP enrichment using this antibody (Supplementary Fig. 9a).

In parallel, we microscopically examined the binding of synaptamide to GPR110 in living cells by using a fluorescent analogue of synaptamide, bodipy-synaptamide 10-(5-((2-((4*Z*,7Z,10*Z*,13*Z*,16*Z*,19*Z*)-docosa-4,7,10,13,16,19-hexaenamido)ethyl)amino)-5-oxopentyl)-5,5-difluoro-1,3,7,9-tetramethyl-5*H*-dipyrrolo(1,2-c:2',1'-f)(1,3,2)diazaborinin-4-ium-5-uide, (Fig. 3e). This analogue also retained bioactivity comparable to synaptamide as indicated by the cAMP production in cultured cortical neurons (Fig. 3f). As the fatty acyl ethanolamides are lipophilic, non-specific binding to cell membranes made it difficult to microscopically determine specific binding of bodipy-synaptamide to the receptors on the cell surface. For this reason, we have examined endocytic receptors as fluorescent puncta based on the fact that most GPCRs are internalized after ligand binding^27^. Indeed, the cortical neurons treated with bodipy-synaptamide for 30 min showed punctate fluorescence signals in the cell body and neurites (Fig. 3g). We observed that an *N*-terminal targeting mGPR110 antibody used to detect full length GPR110 in Supplementary Figs S6a and S9a dose-dependently decreased synaptamide-induced cAMP production in both cortical neurons and NSCs (Supplementary Fig. 9b), suggesting that this antibody successfully interfered with the binding of synaptamide to GPR110. When GPR110 is blocked by the pretreatment with this *N*-terminal targeting anti-GPR110 antibody under a non-permeable condition, the fluorescent puncta were no longer visible, indicating that those puncta signals were derived from the binding of bodipy-synaptamide to GPR110 on the surface of cortical neurons. The pretreatment of the neurons with synaptamide also abolished the puncta, indicating the binding of synaptamide to GPR110. Both microscopic and in-cell cross-linking data consistently demonstrated that synaptamide binds to GPR110 in living cells. Synaptamide potently induced cAMP production with EC_50_ in the low 2–5 nM range in cultured cortical neurons and NSCs (Fig. 3h). The same *N*-terminal targeting GPR110 antibody completely blocked the synaptamide-induced cAMP production, indicating the importance of synaptamide binding to GPR110 to increase cAMP. A physical binding assay based on microscale thermophoresis^28^,^29^ using immunopurified hGPR110 and bodipy-synaptamide, a fluorescent synaptamide analogue, indicated apparent Kd in the low nM range (Supplementary Fig. 10), further supporting synaptamide as a potent GPR110 ligand.

Neither GPR110 binding nor synaptamide-like bioactivity was observed with other endogenous *N*-acylethanolamines. Only synaptamide blocked cellular binding of bodipy-synaptamide detected as the fluorescent endocytic receptor puncta (Supplementary Fig. 11), and significantly displaced G1 bound to GPR110 (Fig. 2e), indicating that synaptamide is a specific ligand to GPR110. Accordingly, other fatty acid ethanolamides did not significantly induce cAMP production (Supplementary Fig. 12a) or cortical neurite outgrowth (Supplementary Fig. 12b).

### *N*-terminal part of GPR110 is important for synaptamide binding

**Figure 4:**
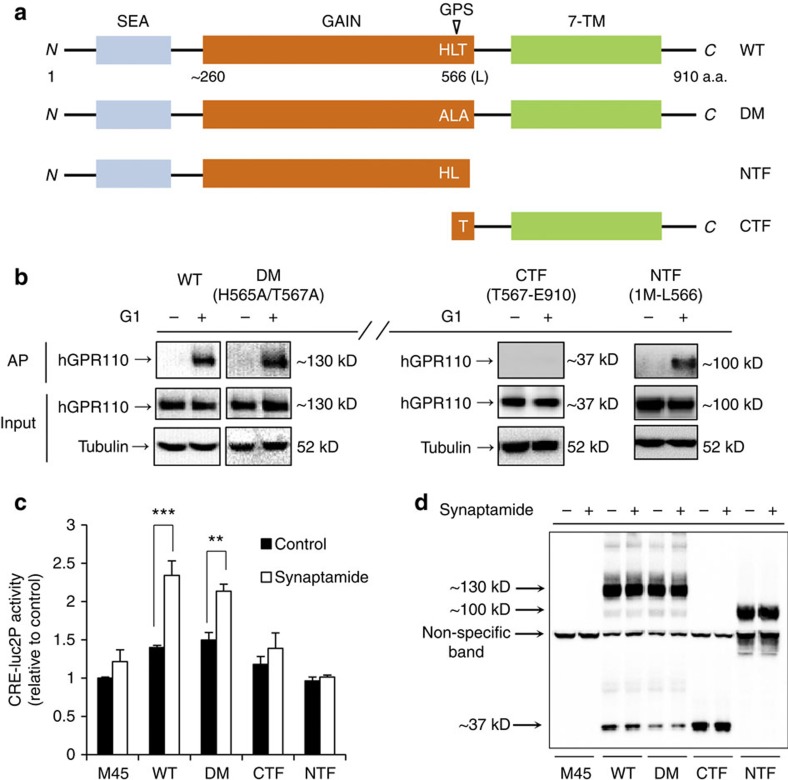
Significance of GPR110 *N*-terminus in ligand binding and synaptamide-induced cAMP production. Human GPR110 sequence map shows wild type full size hGPR110 (1–910, WT), GPS cleavage sequence (HL̂T) double mutant (H565A/T567A, DM), *N*-terminal fragment (1–566, NTF) and *C*-terminal fragment (567–910, CTF) (**a**). When the *N*-terminus was truncated (CTF), neither binding of synaptamide analogues G1 to hGPR110 (**b**) nor synaptamide-induced cAMP production (**c**) was observed, while the *C*-terminal truncated form, NTF, showed G1 binding. Double mutation at GPS (DM) to prevent the autocleavage did not alter the ligand binding ability or synaptamide-induced cAMP production. The treatment with 10 nM synaptamide did not affect hGPR110 autocleavage (**d**). To evaluate cAMP production, various mutant forms of hGPR110 were transiently overexpressed in HEK293 cells permanently expressing CRE-luc2P as a cAMP sensor and treated with 10 nM synaptamide for 16 h. M45: empty vector expressing cells. Data represent three independent experiments (**b**–**d**). Values in **c** are means±s.e.m. of biological triplicates (*n*=3). ***P<0.001 versus the non-synaptamide treated control by unpaired Student *t*-test.

The observation that the *N*-terminal targeting antibody blocked the synaptamide binding to GPR110 (Fig. 3g) and synaptamide-induced cAMP production (Fig. 3h, Supplementary Fig. 9b) led us to test the significance of GPR110 *N*-terminus in synaptamide-induced GPR110 activation. GPR110 has a long *N*-terminus with an autoproteolysis-inducing (GAIN) domain containing a GPCR proteolysis site (GPS). As a cleaved form derived from GPS hydrolysis is considered important for GPR110 activation^30^, we prepared the HA-tagged mutant forms shown in Fig. 4a. These hGPR110 mutants overexpressed in HEK-CRE-luc2P cells were tested for synaptamide analogue G1 binding and cAMP production (Fig. 4b,c). Double mutation at GPS (DM, H565A/T567A) to minimize the autocleavage at GPS altered neither ligand binding ability nor synaptamide-induced cAMP production observed with wild type GPR110 (WT). When the *N*-terminus was truncated at GPS (CTF), both G1-binding and cAMP production induced by synaptamide were either no longer apparent or greatly diminished at best. In contrast, the *N*-terminal side of the cleavage product (NTF) showed considerable binding to G1 although cAMP production was not induced as expected from the lack of the *C*-terminus for G-protein coupling. Furthermore, synaptamide did not affect GPR110 autocleavage (Fig. 4d). These data clearly indicate the importance of the *N*-terminal sequence for synaptamide-induced GPR110 activation.

### GPR110 is a functional receptor for synaptamide

**Figure 5:**
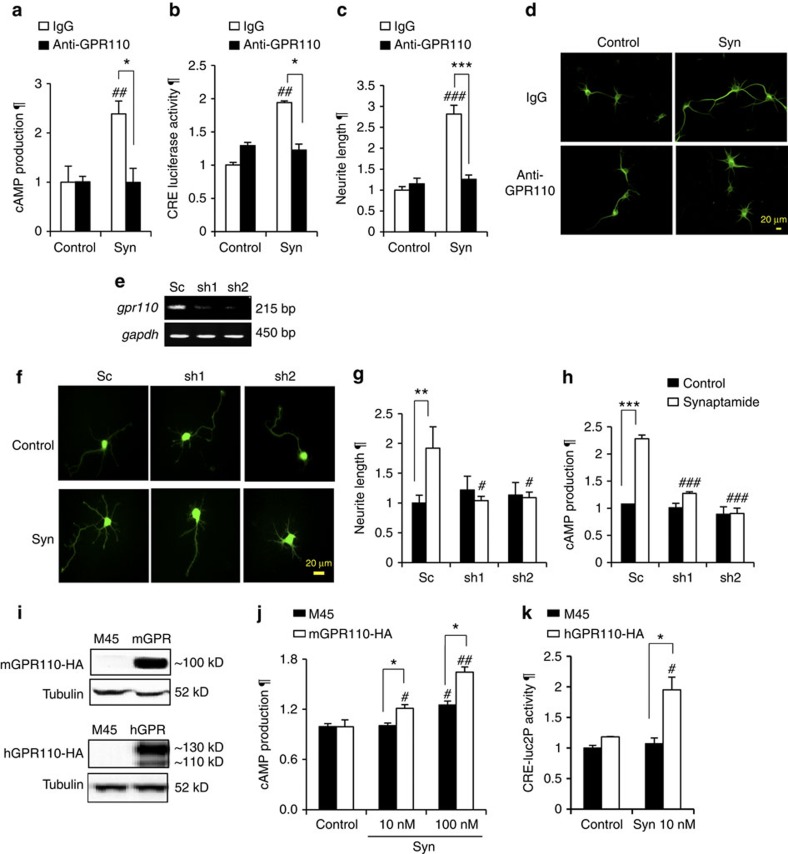
Synaptamide-induced cAMP production and neurite outgrowth affected by GPR110 knockdown, pretreatment with anti-GPR110 antibody or GPR110 overexpression. Cortical neurons were pre-treated with 0.4 μg ml^−1^ *N*-terminal targeting anti-GPR110 antibody for 30 min on DIV1 or DIV3, and incubated with 10 nM synaptamide for 48 h or 10 min for the evaluation of neurite outgrowth or cAMP production, respectively. The GPR110 antibody pretreatment abolished synaptamide-induced increases in cAMP (**a**), CRE activity (**b**) and MAP2-immunostained neurite outgrowth (**c**,**d**). Transfection of cortical cells with GFP-expressing GPR110 shRNAs (sh1 and sh2) performed on day 0 effectively suppressed GPR110 expression on DIV3 (**e**). shRNA expression prevented synaptamide-induced neurite outgrowth (**f**,**g**) and cAMP production (**h**) while scrambled control RNA (Sc) showed no effects. For cAMP assay, transfected cortical cells were treated with 10 nM synaptamide on DIV3 for 10 min. Neurite outgrowth was evaluated for transfected (GFP-positive) cells after treatment with 10 nM synaptamide on DIV1 for 2 days. Overexpression of HA-tagged mGPR110 in A549 cells or HA-tagged hGPR110 in CRE-luc2P HEK cells for 24 h (**i**) significantly increased cAMP production after 10 min stimulation with 10–100 nM synaptamide compared with the M45 empty-vector expressing cells (**j**,**k**). Data represent three independent experiments. Values are shown as means±s.e.m. of biological triplicates (*n*=3). *P<0.05, **P<0.01, ***P<0.001, ^#^*P*<0.05, ^##^*P*<0.01, ^###^*P*<0.001 by unpaired Student *t*-test versus the corresponding control. ¶, relative to control. Scale bars, 20 μm (**d**,**f**).

The functional significance of GPR110 was tested for synaptamide-induced cAMP production and neurite outgrowth after disrupting synaptamide-GPR110 interaction through pretreatment with the *N*-terminal targeting GPR110 antibody described above or by GPR110 gene silencing or overexpression (Fig. 5). Blocking GPR110 by pretreatment of cortical neurons with the GPR110 antibody for 30 min prevented a synaptamide-induced increase in cAMP (Fig. 5a), CRE transcriptional activity (Fig. 5b) and neurite outgrowth (Fig. 5c,d). Similarly, synaptamide-induced cAMP production and neurogenic differentiation of NSCs was diminished after pretreatment with the same GPR110 antibody while the corresponding IgG control did not show any effect (Supplementary Fig. 13). GPR110 gene knockdown consistently revealed a critical role of GPR110 in mediating synaptamide bioactivity. The GPR110 expression in cortical cells was successfully silenced after transfection with GFP-expressing shRNAs compared with the scrambled control RNA (Sc) (Fig. 5e). The GPR110 knockdown almost completely abolished synaptamide-induced neurite outgrowth (Fig. 5f,g) and cAMP production (Fig. 5h). In contrast to the gene silencing, GPR110 overexpression potentiated synaptamide-derived bioactivity (Fig. 5i–k). Synaptamide at 100 nM slightly but significantly increased cAMP production in A549 human lung cancer cells where endogenous expression of GPR110 was noted previously^24^ (Fig. 5j). Overexpression of mGPR110 in A549 cells further increased the synaptamide effect, and this was abrogated by the pretreatment with GPR110 antibody (Supplementary Fig. 14). Likewise, when hGPR110 was transiently overexpressed in HEK293-CRE-luc2P cells, 10 nM synaptamide significantly increased the cAMP-driven CRE activity, while no synaptamide effect was observed in empty vector-transfected control cells. These data altogether with physical binding of synaptamide to GPR110 clearly indicate that GPR110 is a functional receptor for synaptamide.

### GPR110 expression in brain

**Figure 6:**
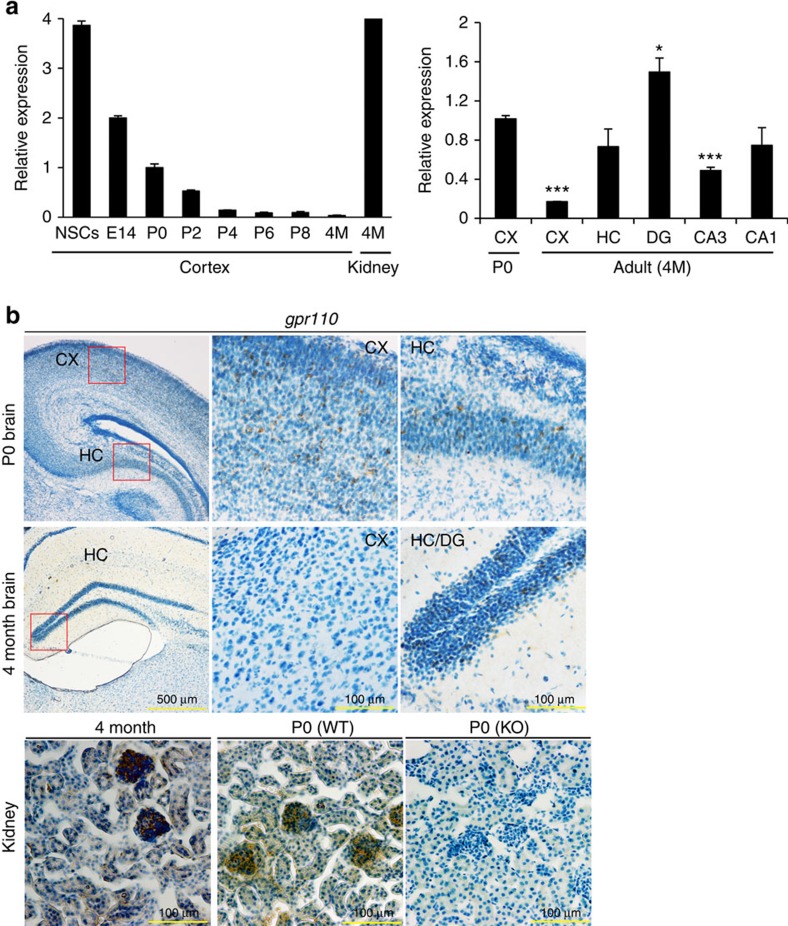
Expression of *gpr*110 transcripts in the mouse brain. Temporal and regional expression of GPR110 mRNA evaluated by quantitative real-time PCR indicated high expression in NSCs and fetal brain (FB) as well as in hippocampus (HC), particularly the dentate gyrus (DG) area, in 4 month (4 M) old adult brains (**a**). The expression was normalized to the level in the P0 cortex (**b**). *In situ* hybridization also indicated the considerable expression of *gpr*110 in P0 brain cortex and hippocampus as wells as adult hippocampal DG area while adult cortical region showed rare expression (**b**). Coronal sections of newborn and adult mouse brains are shown. The boxed areas in the micrographs on the far left panels are shown at a higher magnification for CX and HC regions in the two right panels. Nuclei were counterstained with hematoxylin. Positive staining is indicated by the high expression in glomeruli of P0 and adult kidney. The P0 kidney from KO mouse was used as a negative control. CX, cerebral cortex. Data represent three (**a**) or five (**b**) independent experiments. Data in **a** are means±s.e.m. of biological triplicates (*n*=3). *P<0.05, ***P<0.001 by unpaired Student *t*-test versus newborn (P0) cortex.

The temporal and regional specificity of GPR110 expression in the brain was apparent. The GPR110 mRNA expression was notably high in NSCs and fetal brains (E14) and decreased significantly after birth (Fig. 6a). In 4 month old adult brains, cortical expression of GPR110 is minimal as reported earlier (Lum *et al*.^24^); however, hippocampus showed a considerable signal at a level comparable to that of newborn brains (P0). The dentate gyrus (DG) in particular, where neurogenesis occurs in adulthood, showed higher GPR110 expression in comparison to other hippocampal regions. *In situ* hybridization also indicated expression of *gpr110* in the P0 brain and adult hippocampal DG, confirming the qPCR results (Fig. 6b). The *gpr110* was strongly detected in the glomerular structure of kidney but was not detected in the kidney from GPR110 KO animals, indicating that the probe used for this assay was specific to *gpr110*. Similarly, the *gpr110* signal detected after overexpression in HEK cells was diminished after co-expression with shRNAs used in Fig. 5e (Supplementary Fig. 15), also indicating the *in situ* hybridization signal was specific for *gpr110*. Higher expression of GPR110 in the DG area in the adult stage suggests a role of synaptamide/GPR110 signalling in adult neurogenesis *in vivo*.

### GPR110 KO abolishes synaptamide bioactivity

**Figure 7:**
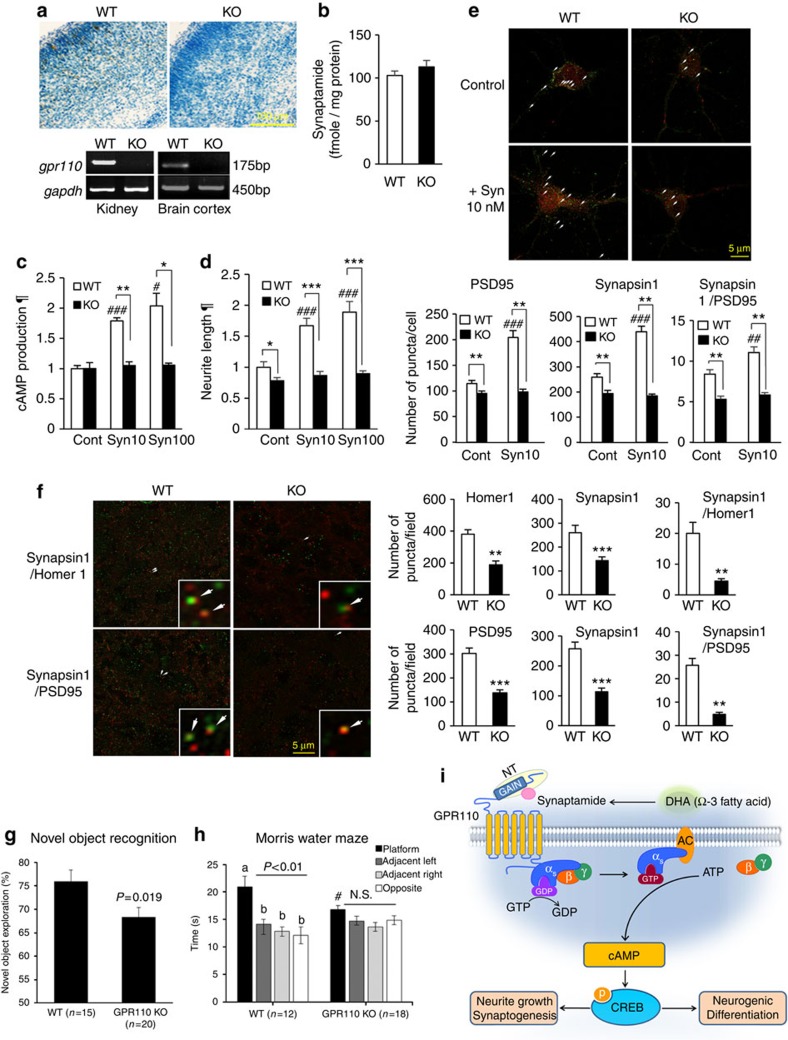
Synaptamide bioactivity abolished by GPR110 KO. No expression of gpr110 in GPR110 KO kidney and brain cortex indicated by PCR and *in situ* hybridization (**a**). The endogenous synaptamide level (mean±s.d., biological triplicates, *n*=3) in WT and KO P0 brains determined by mass spectrometry was comparable (**b**). In cortical neurons from KO mice, synaptamide up to 100 nM showed no effect on cAMP production (**c**) or neurite outgrowth (**d**). Co-localization analysis of presynaptic marker synapsin1 (red) and postsynaptic marker PSD95 (green) revealed increased synapses (overlapping puncta indicated by arrows) by synaptamide treatment in WT but not in KO cortical neurons (**e**). For quantification of synaptic protein puncta in primary neuron culture, 20 cells were microscopically scored per condition, and the data represent three independent experiments. *In vivo* synaptogenesis evaluated in cortical synaptic zone of P10 mouse brain showing severe loss of synapsin1, Homer1 and PSD95 puncta as well as synapsin1/Homer1 or synapsin1/PSD95 colocalizing puncta in the KO brain, indicating significant loss of synapses (**f**). KO animals exhibited significantly reduced recognition memory (**g**) and spatial memory compared with WT (**h**). Schematic representation of synaptamide signalling (**i**). DHA-derived synaptamide binds to GPR110 which in turn triggers Gαs activation, cAMP increase and CREB phosphorylation to promote neurite growth, synaptic protein expression and NSC neurogenic differentiation. Data represent three (**a**–**e**) and two (**f**) independent experiments. Values are means±s.e.m. of biological triplicates (*n*=3) unless specified otherwise. *P<0.05, **P<0.01, ***P<0.001, ^#^*P*<0.05, ^##^*P*<0.01, ^###^*P*<0.001 versus corresponding control (**c**–**e**) or WT platform quadrant time (**h**) by unpaired Student *t*-test. For the comparison of the time in four quadrants within the WT or KO group (**h**), one-way ANOVA followed by Tukey *post hoc* test was used with the statistical significance at *P*<0.05. Means designated with the same letter (a or b) are not significantly different. NT, *N*-terminus. N.S., Not significant. ¶, relative to control. Scale bars, 100 μm (**a**) and 5 μm (**e**,**f**).

The essential role of GPR110 in synaptamide-induced bioactivity was further confirmed using GPR110 KO mice recently produced in our laboratory. Both *in situ* hybridization and PCR shown for P0 kidney and brain cortex (Figs 6b and 7a) indicated complete deletion of *gpr110*. The endogenous synaptamide level in the P0 cortex from WT and KO animals was similar (Fig. 7b). While WT cortical neurons significantly increased cAMP production and neurite growth following synaptamide treatment, no synaptamide effect was observed in neurons prepared from KO mice (Fig. 7c,d, Supplementary Fig. 16). Deletion of *gpr110* also prevented synaptamide-induced synaptogenesis seen in WT neurons (Fig. 1b). The expression of synapsin1 and PSD95, as well as the synapse number evaluated by synapsin1/PSD95 overlapping puncta were significantly lower in KO neurons compared with WT (Fig. 7e). More importantly, synaptamide increased neither synapsin1 and PSD95 expression nor synaptogenesis, unlike the case with WT neurons. These data indicate the importance of GPR110 in mediating synaptamide bioactivity. To determine the *in vivo* significance of GPR110, the synapse number was evaluated using synapsin1/PSD95 and synapsin1/Homer1 co-localizing puncta in the cortical synaptic zone (Fig. 7f). Although the endogenous synaptamide level was similar (Fig. 7b), the lack of synaptamide receptor GPR110 resulted in a severe deficit not only in individual synaptic protein puncta but also in synapse number. Western blot analysis indicated decreases in the synapsin1, PSD95 and Homer1 protein level in KO animals (Supplementary Fig. 17), further confirming the synaptic protein loss microscopically evaluated in Fig. 7f. These *in vitro* and *in vivo* data from GPR110 KO mice provide clear evidence that synaptamide promotes synapse development by acting through GPR110.

### GPR110 KO animals exhibit memory deficit

To further determine the *in vivo* significance of GPR110, the memory function was evaluated by novel object recognition and Morris water maze tests. Both WT and GPR110 KO mice at 2–4 months (2.7±0.6 versus 3.0±0.8 month old, WT versus KO; *P*=0.15; *t*-test) explored the novel object for a longer time than the familiar one; however, GPR110 KO mice spent significantly less time exploring the novel object than WT mice (*P*<0.05; *t*-test; Fig. 7g), even though the total exploration time for both objects is similar between the genotypes (17.8±0.78 versus 18.04±0.89 s). In the Morris water maze test, WT and GPR110 KO mice showed similar learning curves from day 1 to day 4, with the escape latencies and distance to platform for GPR110 KO mice being similar to the WT mice (Supplementary Fig. 18), suggesting that both WT and GPR110 KO mice had comparable visual acuity, swimming skills and ability to learn the task of reaching the platform. As escape latencies are not a sensitive measure of reference memory^31^, the probe trials were performed. During the probe trial, WT mice spent significantly more time in the platform quadrant compared with each of the other three quadrants (*P*<0.01; one way ANOVA with Tukey *post hoc* test), indicating intact memory for the platform location (Fig. 7h). In contrast, GPR110 KO mice did not show significant preference for the platform quadrant. The time spent in the platform quadrant was significantly shorter for KO compared with WT animals (*P*<0.05; *t*-test). Impaired performance of GPR110 KO mice observed in the probe trial despite successful learning of the platform location was indicative of impaired spatial memory as has been reported in a number of studies^31^,^32^,^33^.

## Discussion

Synaptamide is an endogenous metabolite of DHA with potent bioactivity on neurite growth and neurogenesis. Taking advantage of chemical analogues of synaptamide, molecular interaction of synaptamide with the target receptor was probed using cell- and mass spectrometry-based approaches in this study. We discovered that synaptamide is an endogenous ligand to the orphan GPR110, triggering Gαs-coupled cAMP production. Synaptamide-induced neurogenic differentiation and neurite outgrowth were dependent on synaptamide binding to GPR110, indicating the importance of the GPR110 signalling for the observed bioactivity of synaptamide. Synaptamide signalling through GPR110 activation leading to neurogenesis, neuritogenesis and synaptogenesis is schematically depicted in Fig. 7i.

The signalling mechanisms as well as biological function are still elusive for many adhesion GPCRs largely due to lack of known agonists, although some activation mechanisms and functional involvement have recently emerged^34^. The GAIN domain/GPS has been shown to mediate receptor activity either in a GPS-cleaved or GPS-intact fashion^35^,^36^. It has been suggested that autoproteolysis at the GPS is critical for the trafficking and function of adhesion GPCRs^37^,^38^, and the resulting short *N*-terminal stalk region of the 7TM domain was recently shown to be a tethered agonist for the activation of GPR56 and GPR110 (ref. 30). In contrast, the proteolytic cleavage is not required for the function of some adhesion GPCRs such as BAI3 (ref. 39), GPR133 (ref. 40) and latrophilin-1 (LAT-1) receptors^41^, suggesting the importance of the highly conserved GAIN/GPS motif itself for receptor signalling. We observed that synaptamide remained capable of binding to and activating the cleavage resistant mutant form (H565A/T567A) of GPR110 (Fig. 4), indicating that GPS cleavage is not necessary for synaptamide-induced GPR110 activation. In fact, synaptamide was not able to bind to or activate GPR110 when the *N*-terminus was truncated, suggesting that this endogenous ligand signals in a manner distinctively different from the GPS cleavage-dependent mechanism. The requirement of the *N*-terminus for synaptamide-induced GPR110 activation enabled us to successfully employ an *N*-terminal targeting antibody as a tool to block the synaptamide action, providing additional evidence that GPR110 is the synaptamide target receptor mediating neurogenic differentiation of NSCs and neurite growth. With synaptamide identified as an endogenous ligand for GPR110 in this study, molecular details of GPR110 activation may now be further investigated under a physiologic context.

Although aGPCRs have seven TM like other GPCR families, intracellular G protein coupling has not been well-established. Enhancement of constitutive GPR110 activity was observed when GPR110 and Gαq were co-overexpressed^42^. In addition, basal G protein activity was shown to increase when isolated GPR110-expressing membranes were reconstituted with Gαq but not with any other G proteins, suggesting Gαq as an exclusive GPR110 partner at least *in vitro*^30^. Nevertheless, it is not clear whether Gαq coupling is an endogenous G protein signalling mechanism for ligand-activated GPR110. In this work, using a co-IP approach, we demonstrated the association of GPR110 with endogenous Gαs but not with Gαi and Gαq, and this coupling was further verified using overexpressed Gαs (Fig. 2i, Supplementary Fig. 8). The fact that synaptamide induces a cAMP increase that is insensitive to pertussis toxin (Fig. 1) is consistent with GPR110 coupling with Gαs.

Synaptamide biosynthesis was widely observed in brain cells and homogenate preparations^10^,^11^,^14^, although the exact biosynthetic mechanism has yet to be established. In addition, endogenous expression of GPR110 was detected in cultured cortical neurons and NSCs (Supplementary Fig. 9), as well as in fetal and adult brain regions (Fig. 6). The persistent presence of both synaptamide biosynthetic activity and GPR110 expression in brain cells from embryonic to adult stages, despite varying extent, suggests sustained operation of synaptamide/GPR110 signalling in the brain. Previously, expression of this receptor was shown to be low except in kidney^24^; however, we found that in the fetal brain and NSCs, GPR110 mRNA expression is notably higher than in postnatal brains, and is even comparable to that in kidney (Fig. 6a). Furthermore, hippocampal DG, which retains neurogenic capacity throughout life^43^, showed sustained expression of GPR110, suggesting a role of GPR110 in promoting neurogenesis even after embryonic development. GPR110 KO mice recently produced in our laboratory showed no appreciable phenotype in terms of viability or fertility as has been previously described^44^; however, detailed phenotypic characterization needs to be performed when a sufficient number of KO animals become available. Nevertheless, GPR110 KO abolished the synaptamide bioactivity (Fig. 7c–e) and significantly reduced synaptic protein expression and synapse number in developing brains (Fig. 7f, Supplementary Fig. 17) despite no alteration in endogenous synaptamide levels (Fig. 7b), indicating the importance of synaptamide action through GPR110 for synapse development. While GPR110 may not be critical for survival, the synaptamide/GPR110 signalling may be an essential mechanism to meet the developmental need for active neurogenesis, neuritogenesis and synaptogenesis which are vital for proper development of brain function. Indeed, the recognition memory and spatial memory were significantly compromised in adult GPR110 KO mice, supporting the notion that developmental deficits in synaptamide/GPR110 signalling can lead to suboptimal brain function at a later stage. Considering the close link between DHA and synaptamide levels in brain^11^,^14^, inadequate synaptamide-activated GPR110 signalling, particularly during development, may provide an explanation for learning disability and memory deficits associated with brain DHA reduction caused by insufficient dietary omega-3 fatty acid intake^45^. Likewise, genetic or environmental modification of GPR110 may also have significant ramifications in development of brain function.

In conclusion, synaptamide, an endogenous metabolite of DHA, is a specific GPR110 ligand which triggers cAMP-dependent signal transduction leading to potent induction of neurogenesis, neuritogenesis and synaptogenesis in developing neurons. GPR110 deorphanized as the functional receptor for synaptamide provides a mechanism that explains the critical importance of omega-3 fatty acid nutrition in brain development. Furthermore, GPR110 emerges as a potential novel target for controlling physiological and pathophysiological processes of neurodevelopment and function.

## Methods

### Animals

Time pregnant female C57BL/6 mice or Wistar rats were obtained from NIH-NCI animal production program or Charles River Laboratories (Portage, MI), respectively, and GPR110 (adhesion G protein-coupled receptor F1: Adgrf1) heterozygous mice on C57BL/6 background were generated by Knockout Mouse Project (KOMP) Repository. Animals were housed in SPF facility and acclimated for a day before brains were collected for the preparation of primary cortical neurons or neural stem cells (NSCs), *in situ* hybridization or immunohistochemistry. GPR110 KO mice were generated by heterozygote mating in our laboratory. All experiments in this study were carried out in accordance with the guiding principles for the care and use of animals approved by the National Institute on Alcohol Abuse and Alcoholism (LMS-HK13 or 41).

### Primary cell culture and transfection

Primary cortical neurons were prepared from C57BL/6 mouse brains according to the established protocol^46^,^47^,^48^. Briefly, cortices were isolated from P0 pups and digested with 100 U of papain for 30 min at 37 °C and mechanically disrupted by pipetting several times in neurobasal medium (Invitrogen, Carlsbad, CA) supplemented with 2% B27 (Invitrogen, Carlsbad, CA) and 1% glutamin/glutamax mixture (1:3) (Invitrogen, Carlsbad, CA). The dissociated cortical neurons were seeded in poly-D-lysine-coated 24-well plates (0.5 × 10^4^ cells per well) or 60-mm dishes (2.5 × 10^6^ cells per dish) for neurite outgrowth analysis or western blotting, respectively. Electroporation of primary cortical cells was performed before plating using an AMAXA V4XC-2024 4D-Nucleofector X kit (Lonza, Basel, Switzerland). In these studies, 5 × 10^6^ cells cortical cells were transferred into a new tube, centrifuged at 800*g* for 10 min at room temperature and the pellet was suspended in 100 μl of transfection solution. After addition of 3 μg of shRNA plasmids, cells were transferred into the electroporation cuvette and pulsed. Subsequently, the pulsed cells were diluted in 1,000 μl of medium and incubated in a humidified CO_2_ incubator (5% CO_2_, 37 °C) for 2 h before the medium change. HEK293 and A549 cells that were authenticated and certified with non-detectable mycoplasma contamination were obtained from American Type Culture Collection (ATCC, Manassas, VA). HEK293 cells were cultured in DMEM (Invitrogen, Carlsbad, CA) with 10% fetal bovine serum (FBS, Invitrogen, Carlsbad, CA) in the humidified CO_2_ incubator. A549 cells were cultured in F12K (Invitrogen, Carlsbad, CA) with 10% FBS. GPR110-HA transfection in HEK293 and A549 cells were performed using Lipofectamin 2000 (Invitrogen) according to the manufacturer's instructions.

NSCs were cultured by the neurosphere method described earlier^11^,^49^. Briefly, rat or mouse forebrain cortices were isolated on E14.5 or E12.5, respectively, and mechanically disrupted into single cells by repeated pipetting in a serum-free conditioned medium (N2 medium) containing DMEM/F12 (1:1), 0.6% (wt per vol) glucose, 0.1125% (wt per vol) sodium bicarbonate, 2 mM l-glutamine, 5 mM HEPES, 100 μg ml^−1^ human apo-transferrin, 20 nM progesterone, 30 nM sodium selenite, 60 μM putrescine and 25 μg ml^−1^ insulin. The dissociated cells were cultured in 60-mm dishes at 5 × 10^5^ cells per dish in N2 medium with 20 ng ml^−1^ basic fibroblast growth factor (bFGF) and 2 μg ml^−1^ heparin in a humidified 5% CO_2_/95% air incubator at 37 °C. Within 3–4 days, the cells grew as free-floating neurospheres that were then collected by centrifugation, mechanically dissociated by pipetting, and passaged twice. The neurospheres enriched with nestin- and SOX2-positive NSCs were subsequently dissociated and 2.5 × 10^5^ cells were plated onto poly-L-ornithine-coated 24-well plates in N2 medium without bFGF and heparin to initiate differentiation^11^.

### Fatty acid or fatty acyl ethanolamide supplementation

Fatty acids and fatty acyl ethanolamides were dissolved in DMSO or methanol, aliquoted under an argon atmosphere and stored at −70 °C until use to prevent oxidation. Cells were treated with DHA, OA (Nu-Chek Prep, Elysian, MN) or fatty acyl ethanolamides such as synaptamide, bodipy-synaptamide, anandamide (AEA), n-3 and n-6 docosapentaenoylethanolamide (DPEAn-3, DPEAn-6), oleoylethanolamide (OEA) or palmitoylethanolamide (PEA), which were first complexed with fatty acid-free bovine serum albumin (BSA) (Sigma, St Louis, MO) in the presence of vitamin E (Sigma)^50^,^51^. The final concentrations of vitamin E and BSA in the culture medium were 40 μM, and 0.01% (wt per vol), respectively. Medium containing 0.01% (wt per vol) BSA and 40 μM vitamin E was used as the vehicle control.

### Immunocytochemistry

For immunofluorescence staining of primary cells, 1.25 × 10^5^ NSCs or 0.5 × 10^4^ cortical cells were cultured in 24-well plates with 0.5 ml medium unless specified otherwise. Cells were fixed with 0.4% paraformaldehyde (Sigma) in PBS (pH 7.4) for 30 min, and blocked with 0.1 M Tris-buffered saline (pH 7.5, TBS) containing 10 % goat serum (Gibco) and 0.3% Triton X-100 (Sigma) for 1 h at 25 °C. The fixed cells were incubated at 4 °C overnight with primary antibodies such as microtubule-associated protein (MAP2, mouse monoclonal 1:1,000; Sigma). After washing with TBS, the cells were incubated with Alexa Fluor 488- or 555-conjugated secondary antibodies (1:1,000, Life Technologies Corporation, Carlsbad, CA) at 25 °C for 60 min. To visualize nuclei, cells were counter-stained with 2 μg ml^−1^ of 4′, 6-diamidino-2-phenylindole (DAPI). After cells were mounted with 80% (vol/vol) glycerol, image data were collected using a fluorescence microscope (IX81; Olympus Corp., Tokyo, Japan) and neurite length was analysed using Metamorph software (Molecular Devices, Sunnyvale, CA). Unless specified otherwise, neurite outgrowth was evaluated for a total of 80–100 neurons per experimental point using the Metamorph neurite outgrowth module, by taking 3–4 images containing 8–10 MAP2-positive neurons per image from triplicate samples. A similar procedure was used for GFP-expressing shRNA transfected neurons, except only the images of neurons that were positive for GFP expression were selected for quantification of MAP2-immunostained neurite outgrowth. Approximately 60 neurons per experimental point were analysed from triplicates in each of three independent experiments. For evaluation of neurogenic differentiation of NSCs, the number of MAP2-positive cells was counted in three separate wells with 6–8 random fields per well for each individual experiment. Analyses were performed blind as to the treatment. At least three independent experiments were performed. The percentages of neuronal cells were calculated against the total DAPI-positive cells which included undifferentiated stem cells and differentiated neuronal and astroglial cells. To determine synaptamide binding to GPR110 in living cells, cortical neurons were treated with 100 nM bodipy-synaptamide for 30 min, in DMEM containing 0.01% BSA and 40 μM vitamin E, and the formation of the endocytic receptor puncta was examined with a Zeiss LSM 700 confocal laser-scanning microscope (Carl Zeiss, Jena, Germany). To visualize the GPR110 membrane localization, HEK-CRE-luc2P cells (Promega) were cultured and transfected with mGPR110-HA (0.2 μg DNA in 2 ml for 5 × 10^3^ cells) for 48 h on poly-lysine coated glass cover slips. For staining under a non-permeable condition, cells were fixed for 10 min in 2% paraformaldehyde at room temperature, and washed three times with PBS at 4 °C. After washing with a detergent-free blocking buffer containing 10% goat serum and 1% BSA, the cells were incubated with anti-GPR110 monoclonal antibody (1:200 dilution, Abmart, NJ, USA) overnight at 4 °C followed by incubation with Fluor 488- or 555-conjugated secondary antibody (1:500 dilution) along with DAPI for 1 h at room temperature for examination with a Zeiss LSM 700 confocal laser-scanning microscope.

### Luciferase assay for CRE activity

Cortical cells were plated in 24-well plates at 2.5 × 10^5^ cells per well and cultured in neurobasal medium supplemented with 2% B27 and 1% glutamine/glutamax mixture (1:3). Transient transfection of inducible CRE-responsive firefly luciferase construct and Renilla luciferase construct (40:1) (Qiagen, Valencia, CA) was performed on DIV 2 using Lipofectamine 2000 (Invitrogen). After transfection for 24 h, cells were stimulated with synaptamide, DHA or OA in DMEM containing 0.01% BSA and 40 μM vitamin E. After 18 h, the CRE activity was assayed using the Dual Luciferase Reporter Assay (Qiagen) and the CRE-responsive firefly luciferase expression was normalized to Renilla expression. The effect of GPR110 overexpression on synaptamide activity was also evaluated using CRE-luc2P HEK293 cells (Promega). CRE-luc2P HEK293 cells were plated in 24-well plates at 0.25 × 10^6^ cells perwell and transfected with full length hGPR110-HA (WT), hGPR110-DM-HA (H565A/T567A GPS double mutant), *N*-terminal truncated hGPR110-HA *C*-terminal fragment (CTF), *C*-terminal truncated hGPR110-HA *N*-terminal fragment (NTF) or empty vector M45 (GeneCopoeia) for 24 h, and stimulated with 10 nM synaptamide, in DMEM containing 0.01% BSA and 40 μM vitamin E. After 16 h, the CRE activity was determined using the Dual-Glo luciferase assay kit (Promega) following the manufacturer's instructions. All cell lines were tested negative for mycoplasma contamination.

### RT-PCR analysis

Brain tissues were homogenized using a glass Teflon homogenizer. RNA was extracted with an RNeasy mini kit (Qiagen) and measured by a NanoDrop 1000 spectrophotometre (Thermo Scientific). cDNA synthesis was carried out using a cDNA reverse transcription kit (Applied Biosystems) according to manufacturer's protocol. Briefly, 1 μg of isolated RNA, 50 nM random RT primer, 0.25 mM each of the dNTPs, 3.33 U μl^−1^ multiscribe reverse transcriptase, and 0.25 U μl^−1^ RNAase inhibitor were used for each RT reaction. The forward/reverse primers used in polymerization chain reactions include 5′-CCAAGAGAAGCCAAACCTCC-3′/5′-TTCGATAAGCCAGCAGGATG-3′ or 5′-AAAGGGTTCCTGGAGTGTGC-3′/5′-AAGTGCCCCAAACTTTTGCT-3′ for GPR110 and 5′-ACCACAGTCCATGCCATCAC-3′/5′-CACCACCCTGTTGCTGTAGCC-3′ for glyceraldehydes-3-phosphate dehydrogenase (GAPDH), respectively. Quantitative real-time PCR was carried out using a QuantiTect SYBR Green PCR kit (Qiagen) and an ABI Prism 7900 HT system. Briefly, 1 μl cDNA, 6.5 μl of QuantiTect SYBR Green master mix, 1 μl each of GPR110 primers and the control GAPDH gene was used in each PCR. The thermal cycling program was composed of 2 min at 50 °C, 15 min at 95 °C followed by 40 cycles of denaturation at 95 °C for 15 s, annealing at 53 or 57 °C for 30 s, and extension at 72 °C for 30 s. The relative quantification of mRNA expression was performed using the comparative threshold method.

### cAMP assay

Approximately, 1.25 × 10^5^ NSCs or 0.25 × 10^6^ cortical cells were cultured in 0.5 ml medium unless otherwise specified. Cultured NSCs (DIV 4) or cortical cells (DIV 3) were treated with synaptamide for 10 min and cAMP levels were determined using cyclic AMP XP assay kit (Cell Signaling Technology, Danvers, MA) according to the manufacturer's protocol. Briefly, cAMP from the cell lysate was added to the kit to displace HRP-linked cAMP which was bound to an anti-cAMP XP Rabbit mAb immobilized on a 96-well plate. After removing excess cAMP, HRP substrate TMB was added to determine cAMP concentration colorimetrically. In some cases, cells were pretreated with 100 μM adenylyl cyclase inhibitor SQ22,536 (Sigma) or 0.4 μg ml^−1^ IgG (Cell signaling Technology, Danvers, MA) or *N*-terminal targeting GPR110 antibody (Abmart) for 30 min before the stimulation with synaptamide. For evaluating synaptamide-induced cAMP production in GPR110 overexpressing cells, A549 cells were transfected with HA-tagged mGPR110 or empty vector (M45) for 24 h, and treated with 10–100 nM synaptamide for 10 min. In some cases, the transfected cells were pretreated with 0.4 μg ml^−1^ GPR110 antibody or control IgG for 30 min before the synaptamide addition.

### Affinity purification and MS analysis

Fetal mouse brains or HEK293 cells overexpressing mouse or human full length GPR110-HA (WT), hGPR110-DM-HA (H565A/T567A GPS double mutant), *N*-terminal truncated hGPR110-HA *C*-terminal fragment (CTF), *C*-terminal truncated hGPR110-HA *N*-terminal fragment (NTF) or empty vector M45 (GeneCopoeia) were mildly lysed in phosphate buffered saline (PBS) containing 0.5% Triton X-100 and protease inhibitors. The lysate was treated with 1 μM G1 or biotin control for 20 min followed by incubation with streptavidin beads at 25 °C for 30 min. After washing, the affinity-purified products were eluted with 2 × lithium dodecyl sulphate buffer (LDS) for 30 min at 37 °C and subjected to SDS-PAGE for western blot (WB) or MS analysis. MS analysis and protein identification were carried out using the method published previously^52^. Briefly, the entire gel was cut into several bands and each band diced into small pieces. For identification of G1 binding GPR110-HA, affinity purified GPR110-HA was run on the gel and the bands of interest were cut off in the apparent molecular weight around 90 and 110 kD where positive detection by HA antibody (Santa Cruz Biotechnology, 1:200) was indicated. Following reduction by DTT and alkylation by iodoacetamide, proteins in the gel pieces were digested with trypsin at 37 °C overnight. The tryptic peptides were desalted beforenano-LC-ESI-MS/MS analysis performed on an LTQ-Orbitrap XL mass spectrometre (Thermo Scientific) equipped with an Eksigent nanoLC 1D system. Protein identification was achieved by Mascot search against the NCBInr mouse database. The search parameters included trypsin as the protease with a maximum of two missed cleavages allowed, MS tolerance of 10 p.p.m., fragment ion mass tolerance of 0.5 Da, fixed carbamidomethylation at cysteine residues and variable modification for oxidation at methionine. For analysis of deglycosyaltion, deamidation of asparagine and glutamine was also set as a variable modification. The false positive rate was set at <1%.

### Immunopurification and deglycosylation

HEK cells expressing mouse or human HA-tagged GPR110 were lysed with PBS containing 0.5% Triton X-100 and protease/phasphotase inhibitors followed by immunoprecipitation with anti-HA antibody (Santa Cruz) for co-IP/western blot analysis. For mass spectrometric analysis, the immunoprecipitated GPR110-HA were washed five times with lysis buffer, and GPR110-HA was eluted with HA-peptide (Thermo Fisher, 1 mg ml^−1^ in lysis buffer). For deglycosylation, the immunopurified hGPR110 was subjected to *N*-linked and *O*-linked deglycosylation using PNGase F or a deglycosylation enzyme mix from New England BioLabs in accordance with the manufacturer's instruction. GPR110-HA samples with or without deglycosylation were subjected to SDS-PAGE, Coomassie staining, in-gel tryptic digestion and MS analysis.

### Competition binding assay

HEK293 cells overexpressing human or mouse GPR110-HA was mildly lysed in PBS containing 0.5% Triton X-100 and protease inhibitors. The lysate was treated with with 1 μM G1 at 25 °C for 30 min in the presence of 0–10 μM synaptamide. The treated lysate was incubated with streptavidin beads at 25 °C for 30 min and the beads were washed three times with the lysis buffer. The affinity-purified products were eluted by incubating with 2 × LDS at 37 °C for 30 min and subjected to SDS-PAGE for western blot analysis.

### In-cell cross-linking and in-cell binding assay procedures

GPR110-HA overexpressed HEK293 cells or NSCs were washed with PBS, treated with 1 μM G1* (the cross-linkable analogue of G1) or biotin for 20 min and cross-linked by reacting with 1 mM DSS for 30 min at 25 °C. Cross-linking was quenched with 30 mM Tris–HCl for 10 min. Cells were washed thoroughly using 0.01% BSA-containing buffer and lysed with PBS containing 0.5% Triton X-100 and protease inhibitors. The lysate was incubated with streptavidin beads at 25 °C for 30 min and washed three times. The affinity-purified products were recovered by incubating the streptavidin beads with 2 × LDS at 37 °C for 30 min, and subjected to SDS-PAGE for western blot analysis. To monitor fluorescent endocytic receptor puncta formation in living cells, cortical cells were treated with 100 nM bodipy-synaptamide for 30 min with or without pretreatment with various fatty acyl ethanolamides (1 μM) or 0.4 μg ml^−1^ *N*-terminal targeting GPR110 antibody for 30 min. After washing with PBS-T, the cells were fixed with 0.4% paraformaldehyde (Sigma) in PBS (pH 7.4) for 30 min for microscopic analysis.

### Plasmid constructs and shRNA

CMV expression vectors for *C*-terminal HA-tagged GPR110 constructs were prepared by GeneCopoeia (Rockville, MD). The construct contains the full-length open reading frame sequence of human GPR110 (NM_153840.2) and mouse GPR110 (NM_133776.1). The hGPR110-DM-HA have H565A and T567A GPS double mutations in full-length human GPR110. *N*-terminal fragment (NTF)-HA is a *N*-terminal domain of human GPR110 residues 1–566. *C*-terminal fragment (CTF)-HA is a *C*-terminal domain of human GPR110 residues 567–910. The control empty vector is M45. The pcDNA3 expression vector for GFP-tagged Gαs was kindly provided by Dr Mark Rasenick (University of Illinois College of Medicine, Chicago, IL). Knockdown of GPR110 by shRNA was performed using GPR110 OmicsLink short hairpin RNA clones with target sequences of tgcaggtgacatacagaga for sh1 and cgctggcaaactctttaac for sh2 both of which co-express GFP protein to facilitate visualization of transfected cells. The shrna and non-targeting scrambled shRNA (Sc) vectors were obtained from GeneCopoeia (Rockville, MD).

### Co-immunoprecipitation

HEK293 cells were co-transfected with empty vector (CMV), HA-tagged GPR110, and/or GFP-tagged Gαs by using Lipofectamin 2000 (Invitrogen). Cell lysates were prepared using ice-cold cell lysis buffer (Cell Signaling Technology, Danvers, MA). Cell lysates were pre-cleared with protein A/G-agarose beads to minimize non-specific binding. As an additional control for non-specific binding, samples were also immunoprecipitated with mouse IgG. The GPR110-HA protein was immunoprecipitated from cell lysates (1 mg of total protein for each sample) using anti-HA antibody (Santa Cruz Biotechnology, Inc., Dallas, TX). The co-immunoprecipitated products were subjected to SDS-PAGE and immunoblotted using antibodies against Gαs/i/q or GFP (Millipore, Temecula, CA, 1:1,000). Separately, endogenous Gαs was immunoprecipitated from cell lysates (1 mg of total protein for each sample) using anti-Gαs (Millipore). The samples were then subjected to SDS-PAGE and immunoblotted using anti-HA antibody (Santa Cruz Biotechnology, 1:200). Alternatively, GPR110 from cortical cells was immunoprecipitated using a mouse GPR110 antibody (Abmart, Shanghai, China).

### Western blotting

Proteins in cell lysates (generally 30 μg protein) were electrophoresed in 4–12% Bis-Tris gels at 100 V using MOPS SDS running buffer. Proteins were electrophoretically transferred to a PVDF or nitrocellulose membrane at 100 V for 1.5 h. The membrane was blocked with 5% milk or BSA in TBS containing 0.1% Tween 20 (TBS-T) at room temperature for 1 h. Blots were incubated with primary antibodies at 4 °C overnight. After washing with TBS-T buffer, the membranes were incubated for 60 min with peroxidase-conjugated secondary antibodies in TBS-T, and the labelled proteins were detected with chemiluminescence reagents (Thermo Fisher Scientific, Rockford, IL). Unless specified otherwise, α-tubulin, or β-actin (Abcam, 1:1,000) were used as the loading control for all western blotting analyses. Western blot bands were quantified using a Kodak Gel Logic 440 imaging system with ImageQuant 5.1 software (Molecular Dynamics, Sunnyvale, CA). All uncropped western blots can be found in Supplementary Fig. 22.

### Binding of bodipy-synaptamide to immunopurified hGPR110-HA

Direct binding of the bodipy-synaptamide to hGPR110-HA was measured by microscale thermophoresis (MST) with a Monolith NT.115 instrument (NanoTemper Technologies, Munich, Germany)^28^,^29^. Bodipy-synaptamide at a fixed concentration (20 nM) was mixed with increasing concentrations of immunopurified hGPR110-HA in PBS containing 0.5% Triton X-100 and protease inhibitors. After incubation for 10 min at 4 °C, the samples were centrifuged at 14,000*g* for 4 min and loaded into MST premium capillaries (NanoTemper). Measurements were performed with various LED power. Data analyses were performed using the NanoTemper analysis software.

### RNA *in situ* hybridization

Tissue sections cut at 20 μm thickness were processed for RNA in situ detection using the RNAscope Detection Kit (Chromogenic) according to the manufacturer's instructions (Advanced Cell Diagnostics, Hayward, CA)^53^. Briefly, frozen tissue samples were incubated with Pretreat 1 buffer for 10 min at Room temperature (RT). Slides were boiled in Pretreat 2 buffer for 15 min, followed by incubation with Pretreat 3 buffer for 30 min at 40 °C. Slides were incubated with the relevant probes for 2 h at 40 °C, followed by successive incubation with Amp 1 to 6 reagents. Staining was visualized with 3,3′-diaminobenzine (DAB) for 10 min, then lightly counterstained with Gill's haematoxylin. RNAscope probes used were *gpr110* (NM_ 133776.2, cat#300031) and dapB (negative control probe, EF191515, region: 414–862, cat# 310043).

### Synaptic protein puncta analysis

For synaptic protein puncta analysis, 10 day old (P10) GPR110 KO and littermate WT mice from GPR110 heterogygote mating were used. The brains were fixed overnight with 4% paraformaldehyde in PBS at 4 °C, cryoprotected with 30% sucrose in PBS and then embedded in 30% sucrose in PBS:OCT (Tissue-Tek, Sakura, Japan), and cryosectioned at 15 μm using Leica CM3050S (Leica, Germany). The coronal sections were washed and permeabilized in PBS with 0.2% Triton-X 100 (TBST; Roche, Switzerland) and blocked in 5% Normal Goat Serum (NGS) in PBS-T for 1 h at room temperature. The primary antibodies including Anti-PSD95 (1:500, Thermo, MA), anti-Homer1 (1:500, Abcam, MA) and anti-Synapsin1 (1:250, Cell signaling, MA) were diluted in 5% NGS containing PBST. The brain sections were incubated overnight at 4 °C with these primary antibodies followed by Alexa-fluorophore conjugated secondary antibodies (1:200 in PBST with 5% NGS, Life technology, MA) for 2 h at room temperature. Slides were mounted and images were acquired on a Zeiss LSM700 confocal laser-scanning microscope. Brain sections were stained with pre- (synapasin-1) and post-synaptic (PSD95, Homer1) marker pairs. Three random fields in the cortex synaptic zone were examined per each brain section, three separate sections per mouse and three mice were used for analyses. Five μm thick confocal z-stacks (optical section depth 0.33 μm, 15 optical sections/z-stack) of the synaptic zone in cortex area were imaged at × 100 magnification. Maximum projections of three consecutive optical sections (corresponding to 1 μm total depth) were generated from the original z-stack. Analyses were performed blind as to genotype. A plug-in Puncta Analyzer (available upon request from Duke University at https://sites.duke.edu/eroglulab/publications/) under the Image J analysis software platform was used to count the number of pre-, post-, and co-localized synaptic protein puncta. This quantification method is based on the fact that pre- and post-synaptic proteins are not within the same cellular compartments and would appear co-localized only at synapses due to their close proximity. Details of the quantification method have been described by Ippolito and Eroglu^54^. Briefly, 1 μm thick maximum projections are separated into red and green channels, background subtracted (rolling ball radius=50) to detect discrete puncta without introducing noise. The Puncta Analyzer plugin then uses an algorithm to detect the number of puncta that are in close alignment across the two channels, yielding quantified co-localized puncta. At least five optical z-stack sections per brain section were taken per section and three brain sections per animal were analysed. Considering three random fields examined, a total of 45–60 image datasets per brain region were obtained for each animal. We used three animals for each genotype. For synapse staining on the primary cortical cell, primary cortical neuron cultures from P0 WT or KO mice were immunostained with PSD95 and Synapsin-1antibodies. For quantification of synaptic protein puncta, 20 cells were microscopically scored per well, 3 wells per sample and three independent experiments were conducted.

### Behavioural testing

Animals were housed in SPF facility with a 12 h light dark cycle. Male mice from 2 to 4 months of age were used for behavioural experiments, which were performed during the light cycle with the experimenter blinded to the experimental groups.

#### Novel object recognition test

A modified procedure for novel object recognition test was followed^55^ using the ANY-maze video tracking and analysis software (Stoeling Co., Illinois). A total of 35 mice were used. Mice were introduced in a 40 × 40 cm^2^ opaque acrylic chamber for 5 min each for habituation. On the next day, two identical objects were placed in the chamber ∼10 cm away from the walls and from each other. Mice were allowed to freely explore the two objects for 10 min or till 20 s of exploration. A mouse was considered to explore the objects when it was looking toward the object with its nose within 2 cm of the object. The second trial was performed 24 h later to assess object memory. Mice were placed in the chamber with two objects, one object was from the first trial while the other object was a new object that the mice had not encountered before. The mice were allowed to explore the objects for 10 min or till the total exploration time was 20 s. The positions of the objects were reversed for about half of the mice tested. The floor and the objects were wiped clean with 70% ethanol after each trial.

#### Morris water maze test

A seamless pool with 120 cm diameter was filled with water (22–24 °C) rendered opaque with non-toxic tempera paint. It was divided virtually into four equal sized quadrants. A 10 cm^2^ transparent platform was placed in the centre of one of the quadrants such that it was about 5 mm below the surface of the water. A total of 34 mice were used. Four mice were excluded from the experiment. One mouse had difficulty in swimming; three mice were excluded due to lack of exploration/floating during the acquisition trials. Each mouse was given four trials daily for 4 days. In each trial, the mouse was released in the water maze from random points facing the wall and allowed to swim until it located and climbed onto the submerged platform. In case the mouse was unable to locate or climb onto the platform within 90 s, it was gently guided to the platform and allowed to stay on it for about 10 s before being transferred to the home cage. Extra-maze cues in the room were present to serve as guides for the mice for spatial recognition of the platform location. The time taken by the mice to find the platform and the path length to the platform was assessed using the ANY-maze video tracking and analysis software. A 60 s probe test was conducted on the fifth day after removing the platform^56^ and the time spent by the mice in each of the quadrants was analysed. For Water maze test, one-way ANOVA followed by Tukey *post hoc* test was used with the statistical significance at *P*<0.05. Means designated with the same letter are not significantly different. For comparisons of the quadrant time between WT and KO groups in the probe trial, the *t*-test for unequal variance was used.

### Statistical analysis

Unless specified, all data were obtained from at least triplicate samples and represent at least three independent experiments and presented as mean±s.e.m. Statistical analysis was performed using unpaired student *t*-tests.

### Chemical synthesis of synaptamide analogues

Unless stated otherwise, all reactions were carried out under an atmosphere of dry argon or nitrogen in dried glassware. Indicated reaction temperatures refer to those of the reaction bath, while room temperature is noted as ∼25 °C. All anhydrous solvents, commercially available starting materials, and reagents were purchased from Aldrich Chemical Co. and used as received. Analytical thin layer chromatography (TLC) was performed with Sigma Aldrich TLC plates (60 Å, 250 μm). Chromatography on silica gel was performed using forced flow (liquid) of the indicated solvent system on Biotage KP-Sil pre-packed cartridges and using the Biotage SP-1 automated chromatography system. ^1^H spectra were recorded on a Varian Inova 400 MHz spectrometre. Chemical shifts are reported in p.p.m. with the solvent resonance as the internal standard (MeOH-d4 3.31 p.p.m., DMSO-d_6_ 2.50 p.p.m., for 1H). Data are reported as follows: chemical shift, multiplicity (s=singlet, d=doublet, t=triplet, q=quartet, br s=broad singlet, m=multiplet), coupling constants and number of protons. Analytical purity analysis and retention times (RT) reported here were performed on an Agilent LC/MS (Agilent Technologies, Santa Clara, CA). A Phenomenex Luna C18 column (3 micron, 3 × 75 mm) was used at a temperature of 50 °C. The solvent gradients are mentioned for each compound and consist of a percentage of acetonitrile (containing 0.025% trifluoroacetic acid) in water (containing 0.05% trifluoroacetic acid). A 4.5 min run time at a flow rate of 1 ml min^−1^ was used. Unless specified, the prepared analogues were determined to have >95% purity based on the above methods. Mass determination was performed using an Agilent 6130 mass spectrometre with electrospray ionization (ESI) in the positive ion mode.

#### Preparation of biotinylated synaptamide analogue G1

The synthetic preparation scheme for G1, (4*Z*,7*Z*,10*Z*,13*Z*,16*Z*,19*Z*)-*N*-(2-(5-((3a*S*,4*S*,6a*R*)-2-oxohexahydro-1*H*-thieno(3,4-d)imidazol-4-yl)pentanamido)ethyl)docosa-4,7,10,13,16,19-hexaenamide, is shown in Supplementary Fig. 19. A vial charged with DHA (75 mg, 0.23 mmol) was treated with ethane-1,2-diamine (137 mg, 2.28 mmol) followed by HATU (87 mg, 0.23 mmol) and diisopropylamine (0.060 ml, 0.34 mmol). The reaction was stirred for 16 h, then concentrated, and subjected to purification by silica gel flash chromatography (0–25% MeOH/DCM with 1% NH_4_OH). LC/MS Gradient 40 to 100% acetonitrile (0.05% TFA) over 3.6 min; RT 1.78 min; ESI (M+1)^+^ calculated m/z 371.3, found m/z 371.3. The material obtained (assumed 100% conversion) was treated with biotin (0.084 g, 0.342 mmol), HATU (0.130 g, 0.342 mmol) and diisopropylethylamine (0.060 ml, 0.342 mmol). The reaction was stirred for 2 h, diluted with saturated aqueous NH_4_Cl, the organic layer was separated, dried, and purified by flash silica gel column chromatography (0 to 10% MeOH in DCM) to obtain G1 (25 mg, 0.042 mmol, 18% yield over 2 steps). LC/MS Gradient 40 to 100% acetonitrile (0.05% TFA) over 3.6 min; RT 2.28 min; ESI (M+1)^+^ calculated m/z 597.4, found m/z 597.4. ^1^H NMR (400 MHz, Methanol-*d*_4_) δ 5.47–5.26 (m, 12H), 4.52–4.46 (m, 1H), 4.30 (dd, *J*=8.0, 4.5 Hz, 1H), 3.30–3.26 (m, 4H), 3.25–3.17 (m, 1H), 2.93 (dd, *J*=4.8, 12.7 Hz, 1H), 2.89–2.77 (m, 10H), 2.71 (d, *J*=12.7 Hz, 1H), 2.43–2.33 (m, 2H), 2.31–2.16 (m, 4H), 2.15–2.03 (m, 2H), 1.80–1.52 (m, 4H), 1.50–1.39 (m, 2H), 0.97 (t, *J*=7.6 Hz, 3H).

#### Preparation of cross-linkable analogue G1*

The total synthetic preparation scheme for the cross-linkable analogue G1*, (4*Z*,7*Z*,10*Z*,13*Z*,16*Z*,19*Z*)-*N*-(7-amino-4-(5-((3a*S*,4*S*,6a*R*)-2-oxohexahydro-1*H*-thieno(3,4-d)imidazol-4-yl)pentanamido)heptyl)docosa-4,7,10,13,16,19-hexaenamide, is shown in Supplementary Fig. 20.

4-Methoxybenzyl 4-oxoheptane-1,7-diyldicarbamate (**2**).

5-Oxononanedioic acid (**1**) (636 mg, 3.15 mmol) in benzene (5 ml) was refluxed with triethylamine (0.964 ml, 6.92 mmol) and diphenyl phosphorazidate (1.73 g, 6.29 mmol) for 1 h. Excess *para*-methoxy benzyl alcohol (1.952 ml, 15.73 mmol) was then added and the mixture refluxed for another 16 h (ref. 57). The mixture was cooled, concentrated in vacuo, and purified by silica gel flash chromatography (10–100% EtOAc in hexanes). A solid residue was observed on top of fractions that had product (the product did not ionize well so analysis by LC/MS was misleading and staining during TLC analysis was also weak with KMnO_4_). The fractions were combined to provide **2** (705 mg, 1.49 mmol, 47.4% yield). ^1^H NMR (400 MHz, DMSO-*d*_6_) *δ* 7.31–7.23 (m, 4H), 7.16 (t, *J*=5.9 Hz, 2H), 6.95–6.86 (m, 4H), 4.91 (s, 4H), 3.74 (s, 6H), 2.94 (q, *J*=6.5 Hz, 4H), 2.38 (t, *J*=7.2 Hz, 4H), 1.56 (p, *J*=7.1 Hz, 4H).

Bis(4-methoxybenzyl) (4-aminoheptane-1,7-diyl)dicarbamate (**3**).

**2** (1.32 g, 2.79 mmol) was stirred and heated (∼50 °C) in MeOH (25 ml) to dissolve. Ammonium acetate (5.0 g, 65 mmol) was added and the mixture heated at ∼60 °C for 2 h to ensure imine formation (not monitored). Sodium cyanoborohydride (1.76 g, 27.9 mmol) was then added and the reaction stirred for 12 h. Acetic acid (3.20 ml, 55.9 mmol) was then added and stirred for 15 min. The reaction was concentrated, purified by silica gel flash chromatography (staining of fractions with ninhydrin). ^1^H NMR spectrum showed some impurity at −0.5 to 0.5 p.p.m. The residue was dissolved in DCM and washed with 1 N NaOH. The organic layer became a fine suspension which eventually cleared; the white residue seemed to be in the water layer at the junction of the two layers. The organic layer was dried (MgSO_4_), filtered and concentrated to provide 1.25 g of **3** (94% yield) product. ^1^H NMR (400 MHz, DMSO-*d*_6_) δ 7.31–7.23 (m, 4H), 7.16 (t, *J*=5.8 Hz, 2H), 6.95–6.86 (m, 4H), 4.91 (s, 4H), 3.74 (s, 6H), 2.95 (q, *J*=6.7 Hz, 4H), 1.50–1.21 (m, 8H), 1.15–1.07 (m, 2H). (The CH-NH_2_ appears to be underneath solvent peaks). LC/MS gradient 4 to 100% acetonitrile (0.05% TFA) over 3.0 min; RT 2.94 min; ESI (M+1)^+^ calculated m/z 474.3, found m/z 474.2

Bis(4-methoxybenzyl) (4-(5-((3a*S*,4S,6a*R*)-2-oxohexahydro-1*H*-thieno(3,4-*d*)imidazol-4yl)pentanamido) heptane-1,7-diyl)dicarbamate (**4**).

**3** (243 mg, 0.513 mmol) was treated with biotin (188 mg, 0.770 mmol), HATU (293 mg, 0.770 mmol) and diisopropylethylamine (0.269 ml, 1.54 mmol) in DMF (5 ml) for 2 h. Another reaction was set up with 4-methoxybenzyl 4-aminoheptane-1,7-diyldicarbamate (1.01 g, 2.13 mmol), biotin (0.782 g, 3.20 mmol), HATU (1.22 g, 3.20 mmol) and diisopropylethylamine (1.12 ml, 6.40 mmol) in DMF (10 ml). Both reactions were combined, concentrated via rotatory evaporation to remove about half of the DMF. The rest was purified via by preparative reverse phase column chromatography (5 to 90% MeCN in water, both solvents have 1% TFA, Combi Flash Companion, 100g RediSep Rf Gold column). TLC analysis on fractions was done in normal phase with silica gel plates, elution with ∼10% MeOH/DCM) to yield **4** (1.22 g, 66% yield). LC/MS gradient 4 to 100% acetonitrile (0.05% TFA) over 3.0 min; RT 3.23 min, ESI (M+1)^+^ calculated m/z 700.3, found m/z 700.3. ^1^H NMR (400 MHz, DMSO-*d*_6_) *δ* 7.45 (d, *J*=8.6 Hz, 1H), 7.33–7.21 (m, 4H), 7.12 (t, *J*=5.7 Hz, 2H), 6.95–6.83 (m, 4H), 6.39 (s, 1H), 6.34 (s, 1H), 4.91 (s, 4H), 4.29 (dd, *J*=7.8, 5.0 Hz, 1H), 4.16–4.09 (m, 1H), 3.65 (m, 1H), 3.09 (m, 1H), 2.94 (q, *J*=6.2 Hz, 4H), 2.81 (dd, *J*=12.5, 5.1 Hz, 1H), 2.57 (dd, *J*=12.5, 0.9 Hz, 1H), 2.04 (t, *J*=7.4 Hz, 2H), 1.68–1.13 (m, 14H).

*N*-(1,7-diaminoheptan-4-yl)-5-((3a*S*,4*S*,6a*R*)-2-oxohexahydro-1*H*-thieno(3,4-*d*)imidazol-4-yl)pentanamide (**5**).

**4** (100 mg, 0.143 mmol) was taken in DCM (2 ml). The compound was not completely soluble until trifluoroacetic acid (0.275 ml, 3.57 mmol) was added. The solution turned from pink to magenta over 1 h. The mixture was concentrated, diluted with EtOH, and then concentrated again to get rid of TFA and kept under high vacuum. LC/MS gradient 4–100% acetonitrile (0.05% TFA) over 2.8 min; RT 1.97 min, ESI (M+1)^+^ calculated m/z 372.2, found m/z 372.3. ^1^H NMR (400 MHz, Methanol-*d*_4_) δ 4.51 (ddd, *J*=7.9, 5.0, 1.0 Hz, 1H), 4.31 (dd, *J*=7.9, 4.5 Hz, 1H), 3.88 (dq, *J*=9.6, 5.6, 4.9 Hz, 1H), 3.22 (ddd, *J*=9.7, 5.6, 4.5 Hz, 1H), 3.05–2.82 (m, 5H), 2.71 (d, *J*=12.8 Hz, 1H), 2.26 (t, *J*=7.4 Hz, 2H), 1.83–1.39 (m, 14H). The exchangeable protons (NH and NH_2_) are not observed.

(4*Z*,7*Z*,10*Z*,13*Z*,16*Z*,19*Z*)-*N*-(7-amino-4-(5-((3a*S*,4*S*,6a*R*)-2-oxohexahydro-1*H*-thieno(3,4-*d*)imidazol-4-yl)pentanamido)heptyl)docosa-4,7,10,13,16,19-hexaenamide (**6**, G1*).

The crude diamine **5** was then taken up with DCM/MeOH, passed through a bicarbonate cartridge, concentrated and added a solution in DMF (0.5 ml) to a mixture of DHA (55 mg, 0.17 mmol), HATU (64 mg, 0.17 mmol) and diisopropylethylamine (78 μl, 0.45 mmol) in DMF (1 ml). The reaction mixture was stirred for 15 h and then purified by reverse phase chromatography on a Waters semi-preparative HPLC with a Luna C18 column (5 micron, 30 × 75 mm) at a flow rate of 45 ml min^−1^. A gradient of 10 to 50% acetonitrile in water, each with 0.1% TFA over 8 min was used during the purification. Fractions (collection triggered by UV detection at 220 nM) containing compound were passed through PL HCO_3_ MP Resin cartridges, frozen, and lyophilized. Pure product **6** was isolated in low yield (7.0 mg, 10.3 μmol, 4.6% yield over two steps). LC/MS gradient 4 to 100% acetonitrile (0.05% TFA) over 3.0 min; RT 3.63 min; ESI (M+1)^+^ calculated m/z 682.5, found m/z 682.4. ^1^H NMR (400 MHz, DMSO-*d*_6_) *δ* 7.74 (t, *J*=5.4 Hz, 1H), 7.48 (d, *J*=8.7 Hz, 1H), 6.41 (s, 1H), 6.35 (s, 1H), 5.45–5.18 (m, 12H), 4.36–4.25 (m, 1H), 4.13 (dd, *J*=7.7, 5.3 Hz, 1H), 3.66 (m, 1H), 3.09 (m, 1H), 2.99 (m, 2H), 2.87–2.75 (m, 11H), 2.57 (d, *J*=12.5 Hz, 1H), 2.30–2.18 (m, 2H), 2.14–1.97 (m, 6H), 1.72–1.17 (m, 14H), 0.92 (t, *J*=7.5 Hz, 3H).

#### Preparation of bodipy-synaptamide

The synthetic preparation scheme for bodipy-synaptamide, 10-(5-((2-((4*Z*,7*Z*,10*Z*,13*Z*,16*Z*,19*Z*)-docosa-4,7,10,13,16,19-hexaenamido)ethyl)amino)-5-oxopentyl)-5,5-difluoro-1,3,7,9-tetramethyl-5*H* dipyrrolo(1,2-c:2′,1′-f)(1,3,2)diazaborinin-4-ium-5-uide, is shown in Supplementary Fig. 21. DHA (90 mg, 0.27 mmol) in MeCN (5.0 ml) was treated with ethane-1,2-diamine (165 mg, 2.74 mmol) (precipitation was observed) followed by HATU (125 mg, 0.329 mmol) and diisopropyethylamine (0.07 ml, 0.41 mmol). The resultant slurry was stirred for 16 h and washed with saturated aqueuos NaHCO_3_, extracted with EtOAc. The organic layer was dried (MgSO_4_), concentrated and purified by flash silica gel chromatography (10–30% MeOH/DCM, TLC stained with PAA) to provide (4*Z*,7*Z*,10*Z*,13*Z*,16*Z*,19*Z*)-*N*-(2-aminoethyl)docosa-4,7,10,13,16,19-hexaenamide (6.0 mg, 0.02 mmol, 6 % yield). ^1^H NMR (400 MHz, Chloroform-*d*) δ 6.21 (t, *J*=5.8 Hz, 1H), 5.48–5.23 (m, 12H), 3.33 (q, *J*=5.8 Hz, 2H), 2.94–2.74 (m, 12H), 2.67 (s, 2H), 2.40 (q, *J*=7.1 Hz, 2H), 2.25 (dd, *J*=8.2, 6.6 Hz, 2H), 2.06 (pd, *J*=7.4, 1.4 Hz, 2H), 0.96 (t, *J*=7.5 Hz, 3H). This material (4.8 mg, 0.013 mmol) was then treated with 5-(5,5-difluoro-1,3,7,9-tetramethyl-5*H*-4l^4^,5l^4^-dipyrrolo(1,2-*c*:2',1'-*f*)(1,3,2)diazaborinin-10-yl)pentanoic acid (**1**) (4.5 mg, 0.013 mmol; prepared according to Boldyrev and Molotkovsky^58^ in MeCN (1.0 ml) follwed by HATU (9.8 mg, 0.03 mmol) and triethylamine (3.6 μl, 0.026 mmol). The reaction was stirred for 5 h, diluted with saturated aqueous NH_4_Cl and extracted with ethyl acetate. The organic layer was separated, dried with MgSO4, filtered, concentrated and purified by flash silica gel chromatography 10–100% EtOAc/DCM to provide **2** (6.0 mg, 8.6 μmol, 66% yield). ^1^H NMR (400 MHz, Chloroform-*d*) δ 6.23 (br s, 1H), 6.05 (s, 2H), 6.01 (br s, 1H), 5.45–5.26 (m, 12H), 3.41–3.33 (m, 4H), 3.03–2.92 (m, 2H), 2.91–2.76 (m, 10H), 2.51 (s, 6H), 2.42 (s, 6H), 2.40–2.34 (m, 2H), 2.21 (m, 4H), 2.13–2.01 (m, 2H), 1.83 (m, 2H), 1.66 (m, 2H), 0.97 (td, *J*=7.5, 0.8 Hz, 3H).

### Data availability

Data supporting the findings of this study are available within the article and its Supplementary Information Files and from the corresponding author on reasonable request. Raw mass spectrometry data have been deposited in https://figshare.com and are accessible on request.

## Additional information

**How to cite this article:** Lee, J.-W. *et al*. Orphan GPR110 (ADGRF1) targeted by *N*-docosahexaenoylethanolamine in development of neurons and cognitive function. *Nat. Commun.* **7,** 13123 doi: 10.1038/ncomms13123 (2016).

## Supplementary Material

Supplementary Information Supplementary Figures 1-22, Supplementary Tables 1-6
