# Supplementary material for: Orphan GPR110 (ADGRF1) targeted by N-docosahexaenoylethanolamine in development of neurons and cognitive function
Source: Nat Commun. 2016 Oct 19;7:13123. doi: 10.1038/ncomms13123 (PMC5075789; doi:10.1038/ncomms13123)
Supplement: Supplementary Information — Supplementary Figures 1-22, Supplementary Tables 1-6 [file ncomms13123-s1.pdf]

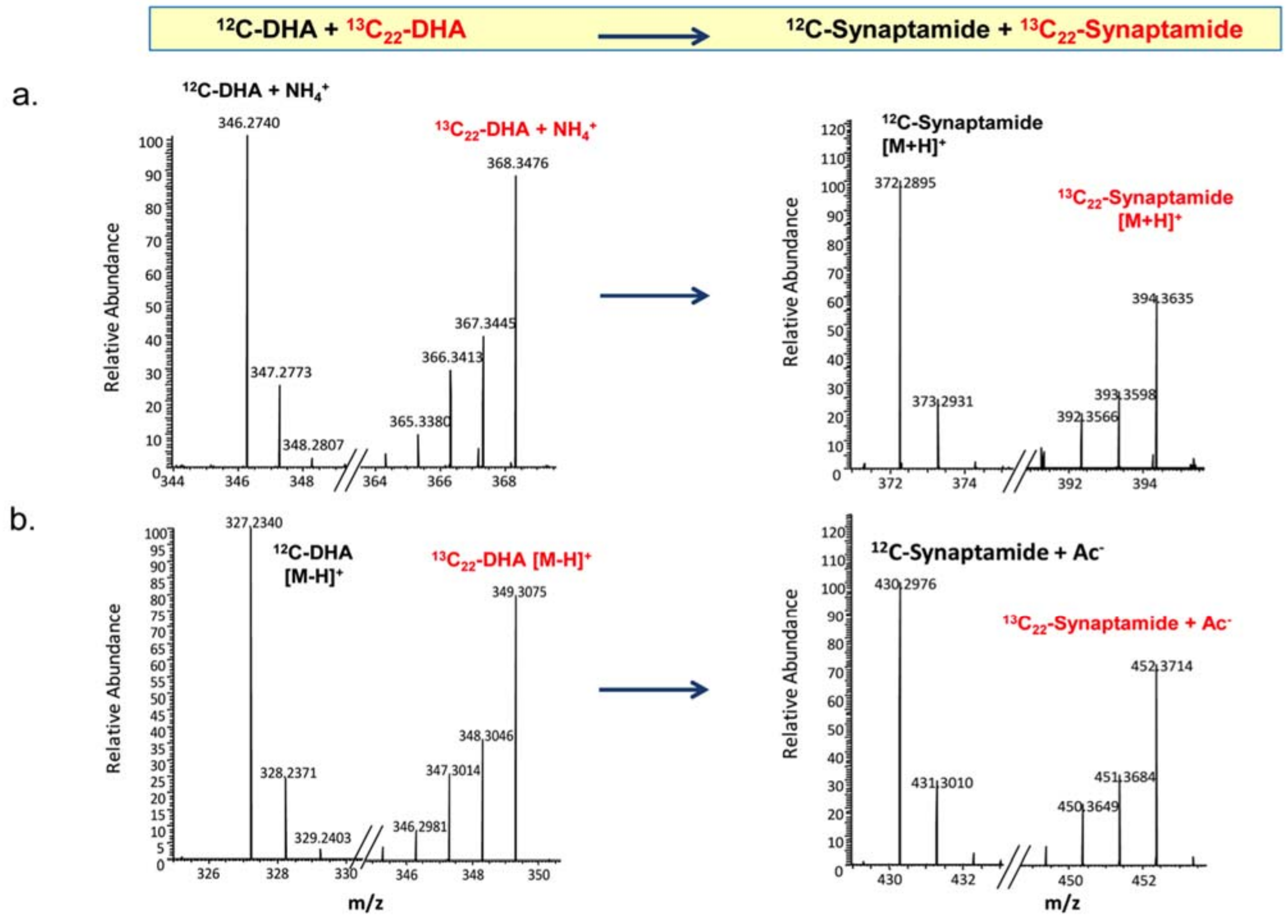

Supplementary Fig. 1. Production of synaptamide from DHA in cortical neuron cultures. When DIV1 cortical neuron cultures were incubated with the mixture of ^{12}C- and uniformly labeled $^{13}C_{22}$-DHA for 1 h, the production of ^{12}C- and $^{13}C_{22}$-synaptamide was detected in the positive (a) and negative ion (b) ESI mass spectra. Data represent at least three independent experiments.

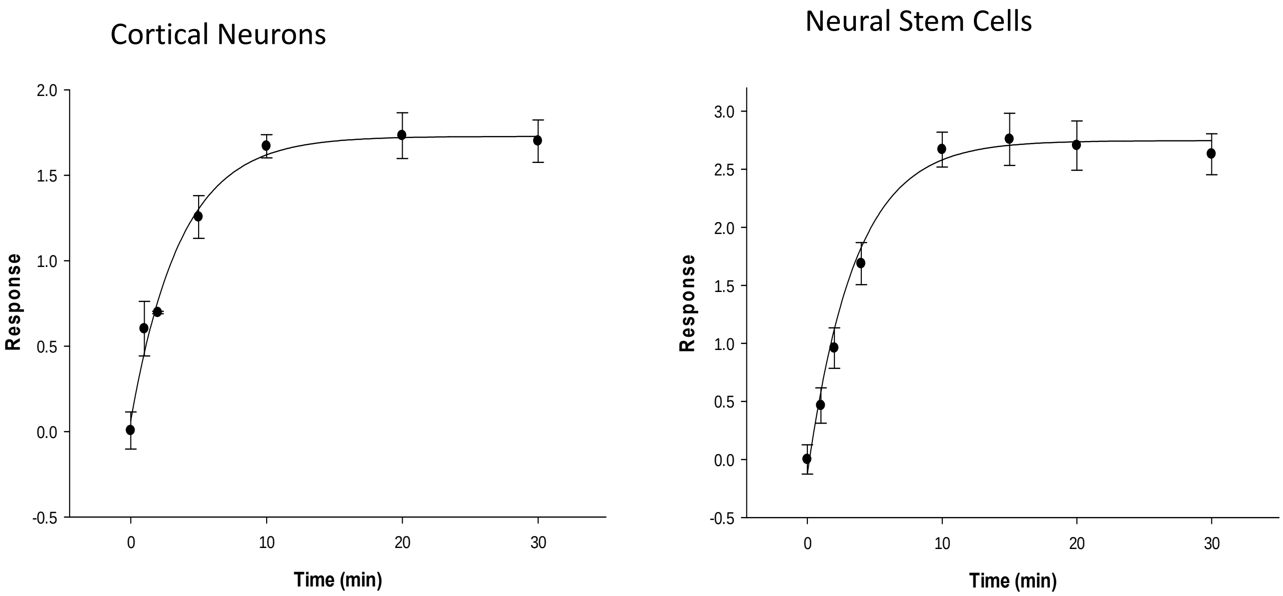

Supplementary Fig. 2. Time course of cAMP production in response to synaptamide in cortical neurons and NSCs. Synaptamide at 10 nM time-dependently increased cAMP production in cultured cortical neurons and NSCs. The cAMP production reached a plateau in 10-15 min. Each data point is presented as mean ± s.e.m. of biological triplicates, representing two independent experiments.

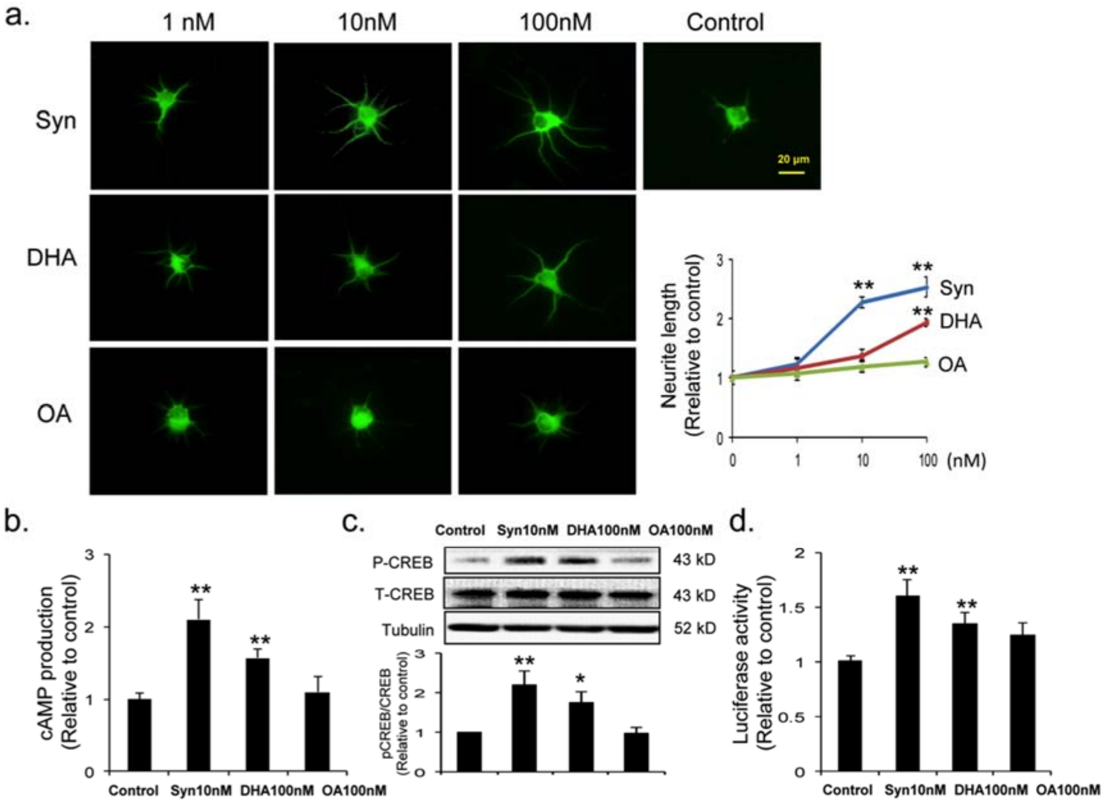

Supplementary Fig. 3. Effects of synaptamide, DHA and OA on neurite growth, cAMP production and CREB activation in cortical neurons. Synaptamide (Syn) dose-dependently increased neurite growth (a). DIV1 neurons were treated with synaptamide for 48 h. cAMP production (b) and CREB phosphorylation (c) were increased after treating DIV3 neurons with synaptamide for 10 min. Similarly, increased CRE transcriptional activity was also observed when DIV2 cortical neurons were transfected with the CRE reporter plasmid for 24 h and treated with synaptamide for additional 18 h (d). Data are presented as means ± s.e.m. of biological triplicates, representing three independent experiments. **p*<0.05, ***p*<0.01 in comparison with control (unpaired Student *t*-test). Scale bar: 20 μm.

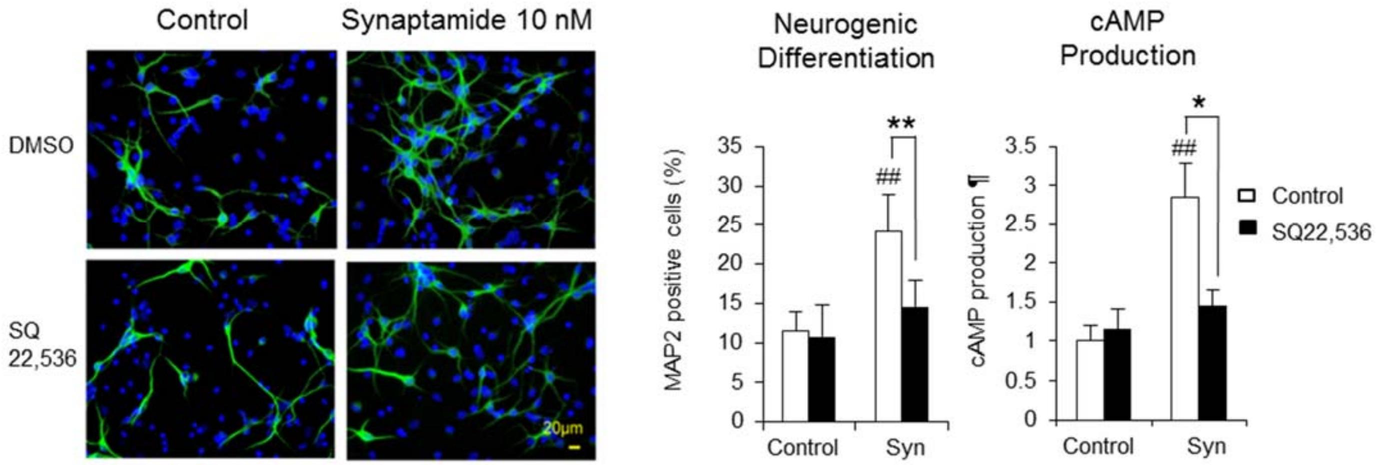

Supplementary Fig. 4. cAMP-dependent neurogenesis induced by synaptamide. Synaptamide-induced neurogenic differentiation and cAMP production in NSCs were abolished by SQ22,536. To evaluate neurogenic differentiation, NSCs were treated with 10 nM synaptamide from 6 h after seeding for 3 days, and DIV3 NSCs were stimulated with synaptamide for 10 min to measure cAMP production. Data are presented as means ± s.e.m. of biological triplicates, representing three independent experiments. $^{\#\#}p<0.01$, $^{*}p<0.05$, $^{**}p<0.01$, *vs.* corresponding control (unpaired Student t-test). ¶, relative to control.

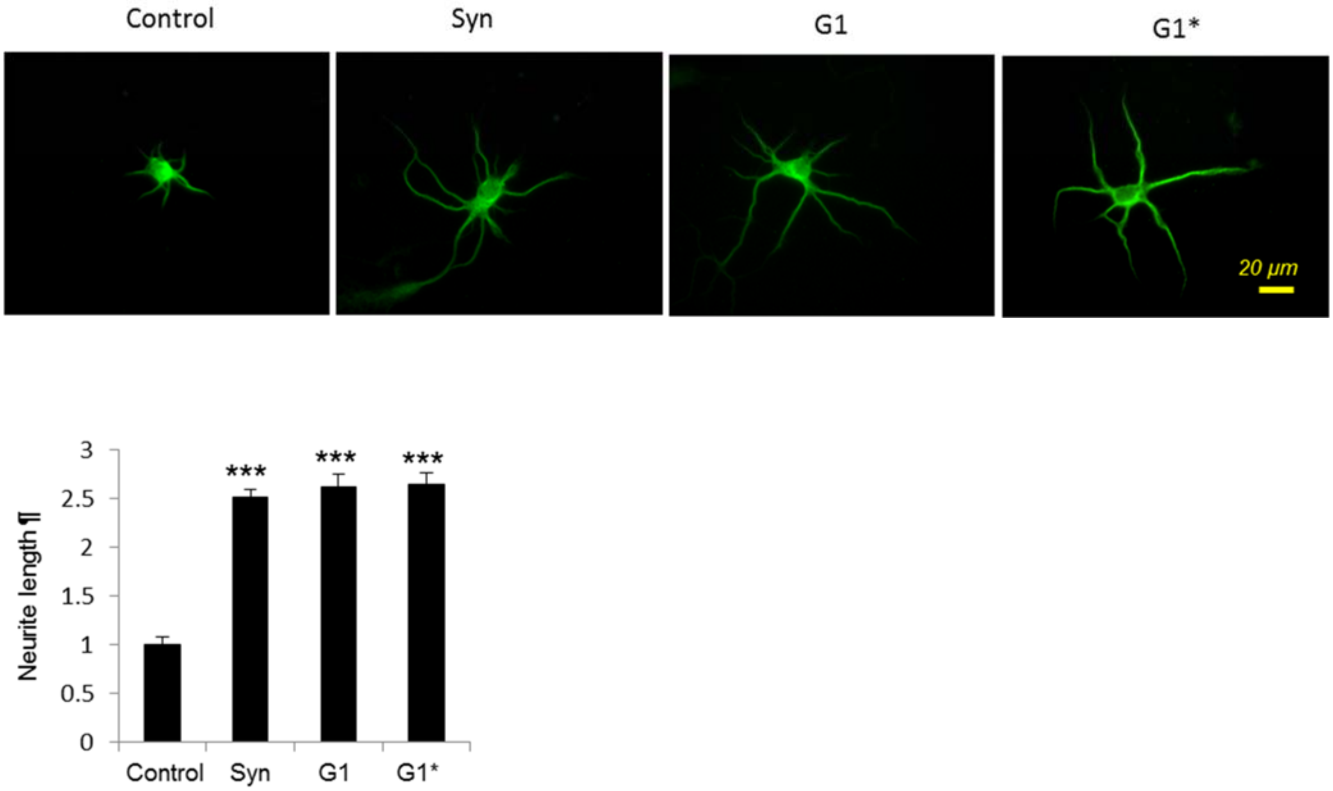

Supplementary Fig. 5. Neurite growth promoted by synaptamide analogues. Synaptamide, biotinylated synaptamide analogue (G1) and biotinylated synaptamide analogue (G1*) containing a crosslinkable primary amine group G1* at 10 nM increased cortical neurite outgrowth to an extent comparable to synaptamide treated on DIV1 for 48 h. Data are means ± s.e.m. of biological triplicates, representing three independent experiments. ***p<0.001 vs. control (unpaired Student *t*-test).

a.

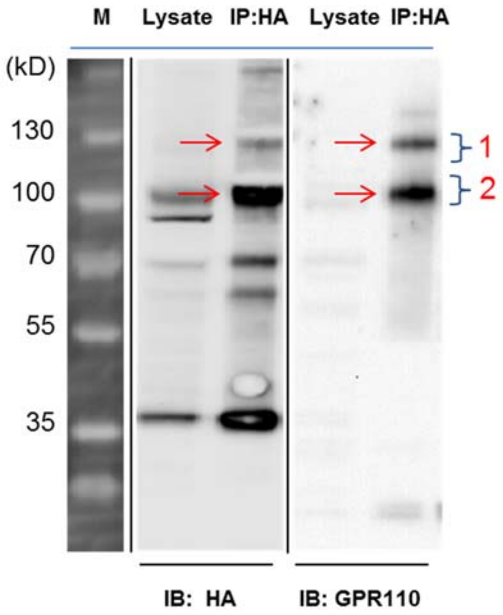

b.

Band 1

Score: 244, identified peptides 10 (red)

Sequence coverage: 14%

1 MRIGLLWLVP LFTLTEGTDG FLQQKNDGRR TKEIVSMVEE RHPVHEYEVL
51 LQVTYRDPEE KRELKRFLKL LRSPSPSLRG PSKIIRVKAT TYCRSRKGFL
101 ECACEDSYTW FPPSCLDPQN CCLHTTGPVP SCNCSLRGLR QSVNFCERAK
151 VWGTFEIDEN FPEDLWNSSS DVYAHYTVGI ENQLKEAYRR VHGFESVRVT
201 QFRKGSIVVG YEVTGSTSPP ELLFAIEQEA EKAQEALRRQ FPVKYGSFRV
251 FGKAPCNSIS FGFGSENDEY TVPCSSGFTG SMTVRCQSSG WQITRESCVL
301 SQLEELKKEL RMIAGKITEA GVASLVQNLS TILLQSPSTT VGNLGSVVSL
351 LSNISSLSLA NSLTVSNLTL MNVINIADHI LDSASITNWT ILLQDAKDAS
401 SQLLKTLESI SSLIPSMALP LNFSGKFIDW KGTPVTQIQS TRGYNYQMEM
451 RQNASLPIRG HVFIEPDQFQ KSHPKTIISM ASLTFGDILP ITQRGNAWVN
501 GPVISTLIQN YSISEIFLNF SKIKGNLTQP RCVFWDFSQL QWSNAGCQLV
551 NETLDTVLCR CSHLTSFSML MSPFVPSSVV PVVKWITYIG LSISIASLIL
601 CLIIESLFWK QTKRSQTSYT RNICLVNIAV SLLIADVWFI IAATVDPSVS
651 PSGVCVAAVF FTHFFYLAVF FWMLVLGILL AYRIILVFHH MALTTMMAIG
701 FCLGYGCPLL ISIITLAVTQ PSNSYKRNDV CWLNWSDKSK PLLAFVVPAL
751 TIVAVNLVVV LLVLRKLWRP AVGERLNQDD KATAIRMGKS LLVLTPLLGL
801 TWGFGIGTMA NSHNLAWHVL FALLNAFQGF FIFCFGILLD TKLRQLLSNK
851 LTTLSSWKQT SKRNASDTVT QPKCLRTFNI LQHRGMYALS HTGDSSSDIT
901 LTQFLSTE

Band 2

Score: 760, identified peptides 20 (red)

Sequence coverage: 17%

1 MRIGLLWLVP LFTLTEGTDG FLQQKNDGRR TKEIVSMVEE RHPVHEYEVL
51 LQVTYRDPEE KRELKRFLKL LRSPSPSLRG PSKIIRVKAT TYCRSRKGFL
101 ECACEDSYTW FPPSCLDPQN CCLHTTGPVP SCNCSLRGLR QSVNFCERAK
151 VWGTFEIDEN FPEDLWNSSS DVYAHYTVGI ENQLKEAYRR VHGFESVRVT
201 QFRKGSIVVG YEVTGSTSPP ELLFAIEQEA EKAQEALRRQ FPVKYGSFRV
251 FGKAPCNSIS FGFGSENDEY TVPCSSGFTG SMTVRCQSSG WQITRESCVL
301 SQLEELKKEL RMIAGKITEA GVASLVQNLS TILLQSPSTT VGNLGSVVSL
351 LSNISSLSLA NSLTVSNLTL MNVINIADHI LDSASITNWT ILLQDAKDAS
401 SQLLKTLESI SSLIPSMALP LNFSGKFIDW KGTPVTQIQS TRGYNYQMEM
451 RQNASLPIRG HVFIEPDQFQ KSHPKTIISM ASLTFGDILP ITQRGNAWVN
501 GPVISTLIQN YSISEIFLNF SKIKGNLTQP RCVFWDFSQL QWSNAGCQLV
551 NETLDTVLCR CSHLTSFSML MSPFVPSSVV PVVKWITYIG LSISIASLIL
601 CLIIESLFWK QTKRSQTSYT RNICLVNIAV SLLIADVWFI IAATVDPSVS
651 PSGVCVAAVF FTHFFYLAVF FWMLVLGILL AYRIILVFHH MALTTMMAIG
701 FCLGYGCPLL ISIITLAVTQ PSNSYKRNDV CWLNWSDKSK PLLAFVVPAL
751 TIVAVNLVVV LLVLRKLWRP AVGERLNQDD KATAIRMGKS LLVLTPLLGL
801 TWGFGIGTMA NSHNLAWHVL FALLNAFQGF FIFCFGILLD TKLRQLLSNK
851 LTTLSSWKQT SKRNASDTVT QPKCLRTFNI LQHRGMYALS HTGDSSSDIT
901 LTQFLSTE

Supplementary Fig. 6. Expression of full length mouse GPR110 (mGPR110) verified by Western blot analysis and mass spectrometry. mGPR110-HA expressed in HEK293 cells and immunopurified (IP) using HA antibody was detected at ~130 (band 1) and ~100 kD (band 2) by both HA and *N*-terminal targeting GPR110 antibodies (a). Mass spectrometric analysis of these bands confirmed that both are full-length GPR110 based on the detected tryptic peptides highlighted in red (b). The peptide sequence highlighted in blue corresponds to the epitope sequence for the *N*-terminal targeting GPR110 antibody used in this study. This antibody did not

detect the C-terminal fragment at ~37 kD, the GPS autocleavage product of GPR110. M: Molecular weight marker. Data represent two independent experimnents.

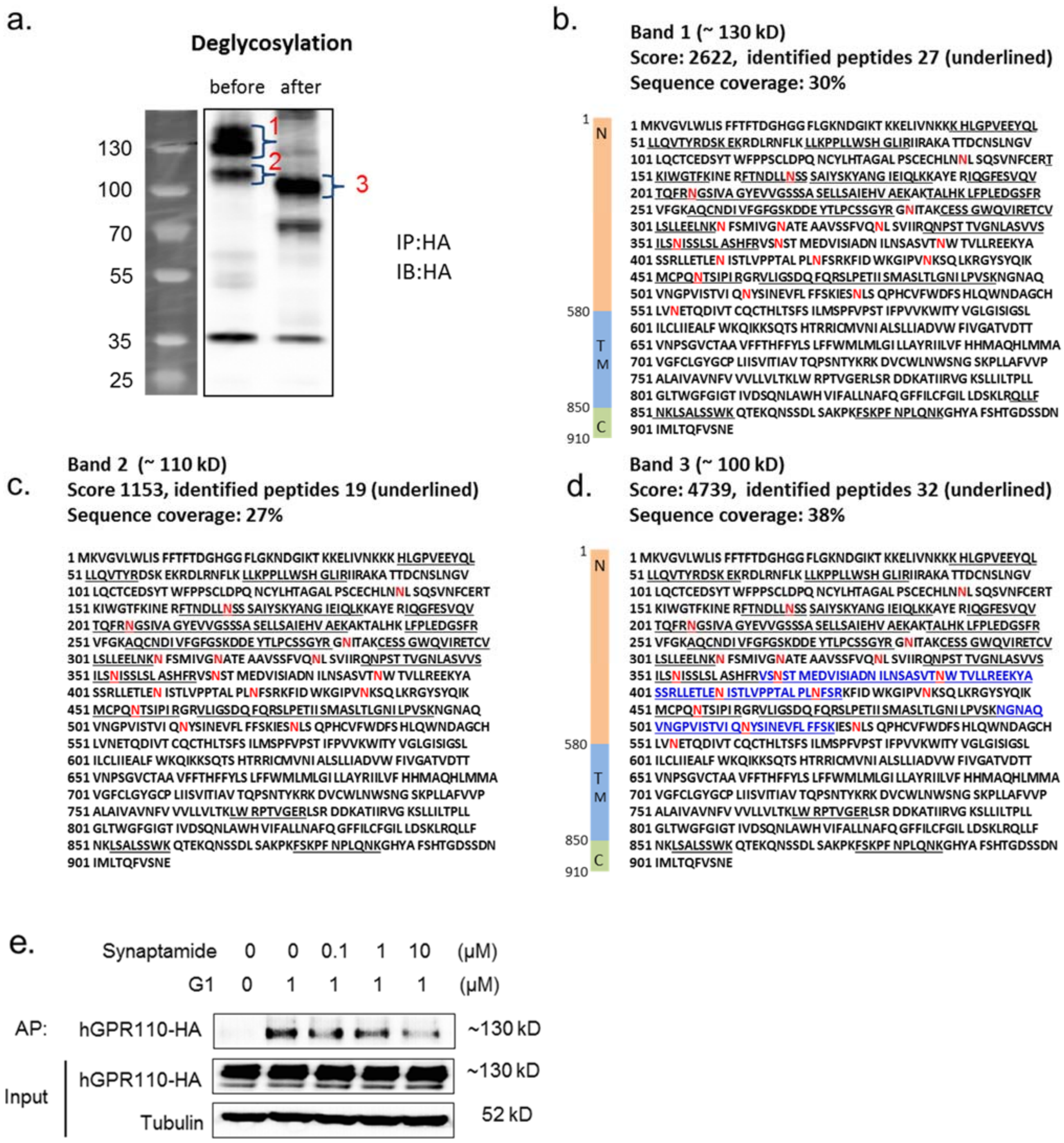

Supplementary Fig. 7. Expression of full length human GPR110 verified by Western blot analysis and mass spectrometry. a, Western blot analysis of hGPR110-HA. HA-tagged GPR110

was overexpressed in HEK293 cells and immunoprecipitated with anti-HA antibody. The immunopurified human GPR110-HA (hGPR110-HA) was detected at ~130 kD (band 1) and ~110 kD (band 2). After deglycosylation using PNGase F, a major band at ~100 kD (band 3) was observed. b-d, Mass spectrometric detection of full-length hGPR110 (gi/61743940). *N*- and *C*-terminal peptides as well as transmembrane (TM) peptides were identified by MS in all of the three bands indicated. The N-linked deglycosylation by PNase F accompanies deamidation of the asparagine residue to aspartic acid, although non-enzymatic deamination can also occur. Five additional *N*-terminal peptides (highlighted in blue), along with deamidation of N368, N389, N410, N423, and N512 were identified from the deglycosylated sample (d), in comparison to the case without treating with the deglycosylation enzyme (b and c). The data suggests that these asparagine residues are most likely the N-linked glycosylation sites of hGPR110. The peptides identified by MS are underlined, and the predicted N-linked glycosylation sites (N-!P-S/T) are highlighted in red in the primary structure of hGPR110. As observed with mGPR110, the G1 binding to hGPR110 was displaced by synaptamide (e), indicating that synaptamide binds to hGPR110. Data represent two independent experimnents.

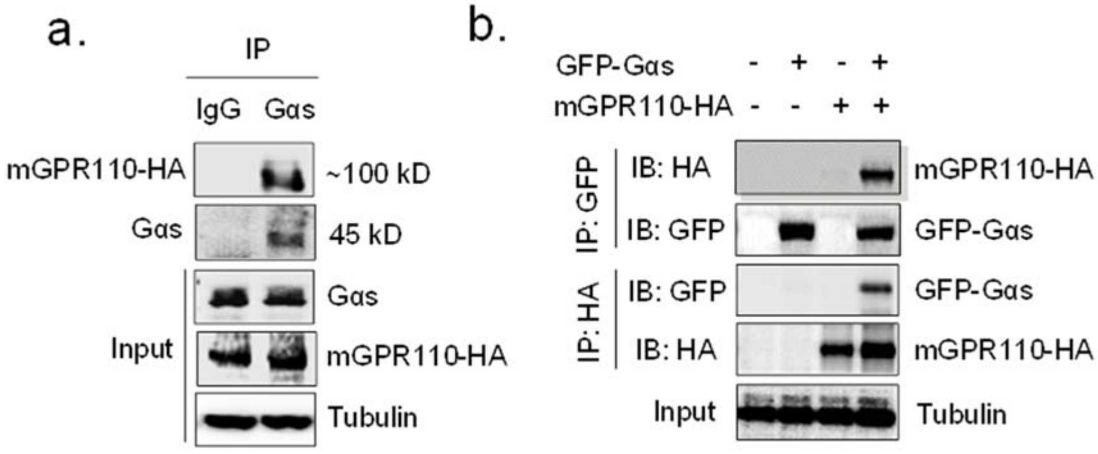

Supplementary Fig. 8. Verification of GPR110 interaction with Gαs by co-immunoprecipitation. GPR110 interaction with Gαs was verified by co-immunoprecipitation of overexpressed mGPR110-HA with endogenous Gαs using Gαs antibody (a). GPR110-HA and GFP-Gαs co-overexpressed in HEK cells were also co-immunoprecipitated using HA or GFP antibody, further confirming the association of GPR110 and Gαs (b). Data represent two independent experimnents.

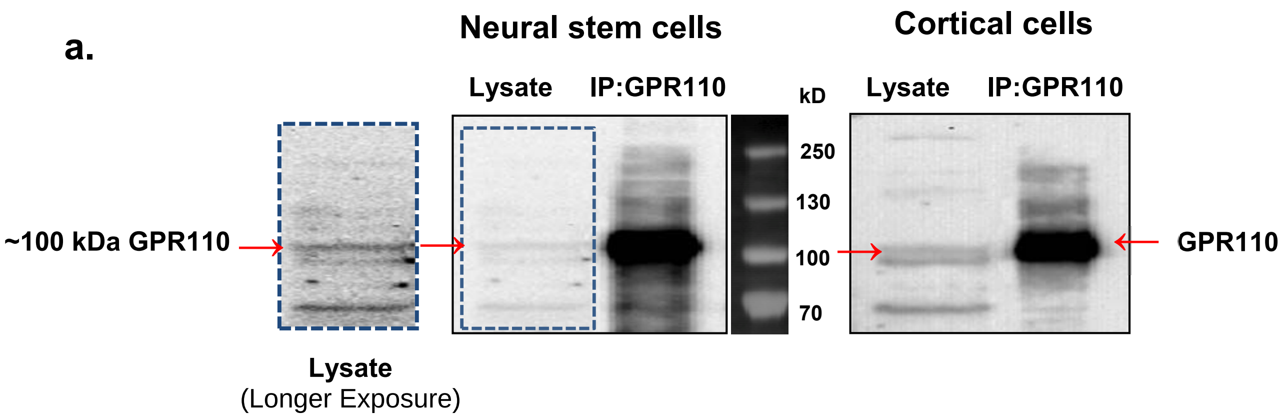

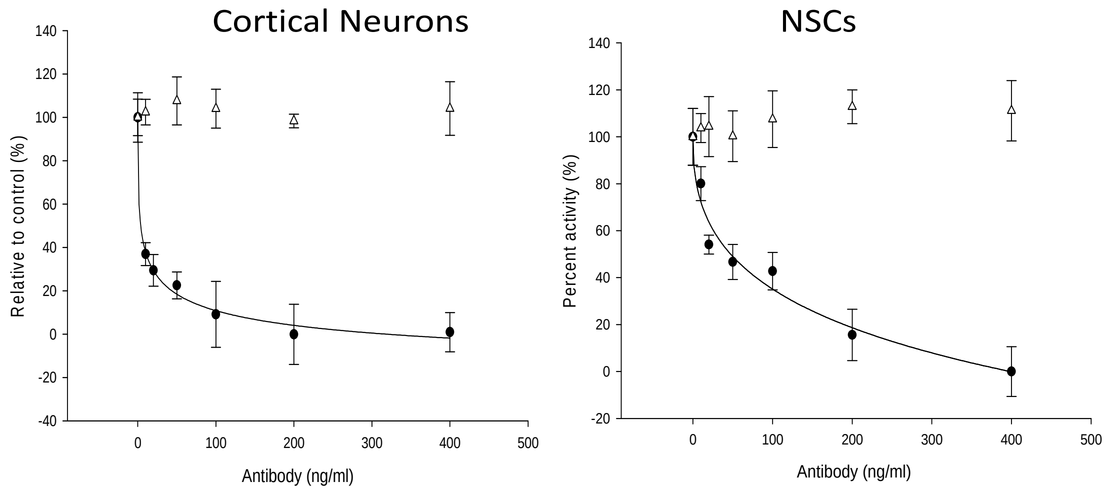

Supplementary Fig. 9. *N*-terminal targeting GPR110 antibody detecting endogenous GPR110 and blocking synaptamide-induced cAMP production in cortical neurons and neural stem cells (NSCs). The *N*-terminal targeting GPR110 antibody detects the expression of GPR110 from the lysate of NSCs and cortical neurons at ~100 kD. Data represent three independent experimnents. Enrichment of GPR110 was indicated after immunopurification with the same GPR110 antibody (a). The GPR110 antibody (closed circle) dose-dependently blocked the cAMP production induced by 10 nM synaptamide while control IgG (open triangle) did not affect

synaptamide-induced cAMP prodcution (b). Each data point in b is presented as mean ± s.e.m. of biological triplicates, representing two independent experiments.

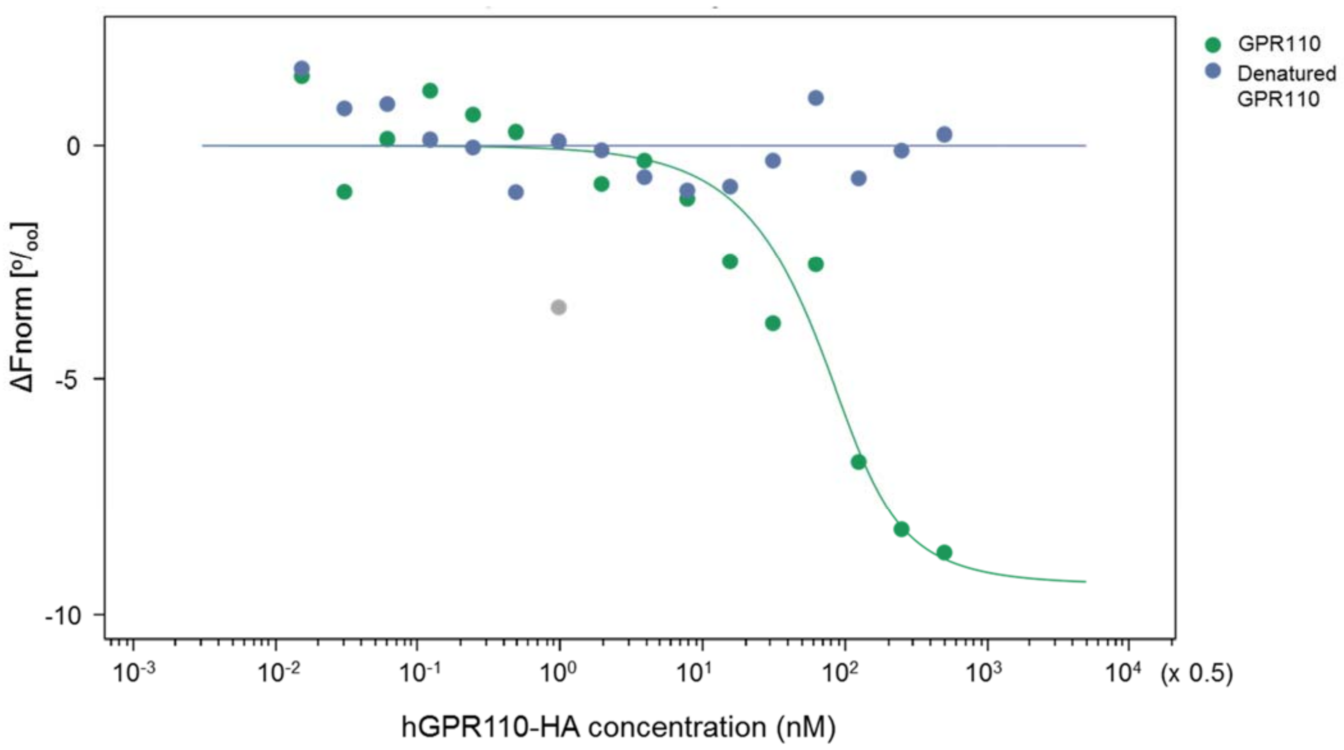

Supplementary Fig. 10. Binding of bodipy-synaptamide to hGPR110-HA. The direct interaction between bodipy-synaptamide, a fluorescent group-labeled synaptamide, and hGPR110-HA immunopurified in PBS containing 0.5% Triton X-100 from HEK cells was assessed by microscale thermophoresis. Under this condition, the apparent Kd was found to be ~20 nM, most likely right-shifted as the bodipy-synaptamide concentration (20 nM) used in this assay was above the low nM Kd expected from the cAMP production (EC50 = 2-5 nM). Nevertheless, the data confirmed the binding between GPR110 and bodipy-synaptamide. No binding was observed when hGPR110-HA was thermally denatured by heating at 60 °C for 30 min. Data represent two independent experimnents.

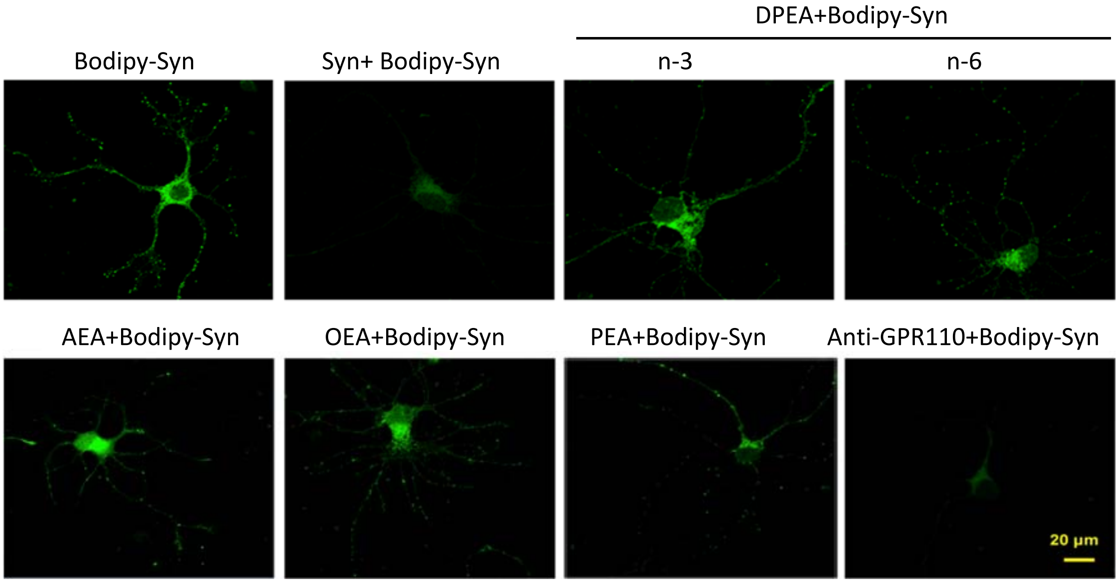

Supplementary Fig. 11. Synaptamide is a specific ligand to GPR110. Only synaptamide (1 µM) and *N*-terminal targeting GPR110 antibody (0.4 µg/mL) among various fatty acyl ethanolamides (1 µM, pretreatment for 30 min) abolished the fluorescent endocytic receptor puncta formed after bodipy-synaptamide (100 nM) treatment for 30 min. Syn, synaptamide; n3, omega-3; n6, omega-6; DPEA, *N*-docosapentaenoylethanolamine; OEA, *N*-oleoylethanolamine; AEA, *N*-arachidonylethanolamine; PEA, *N*-palmitoylethanolamine. Representative data from five independent experimnents are presented.

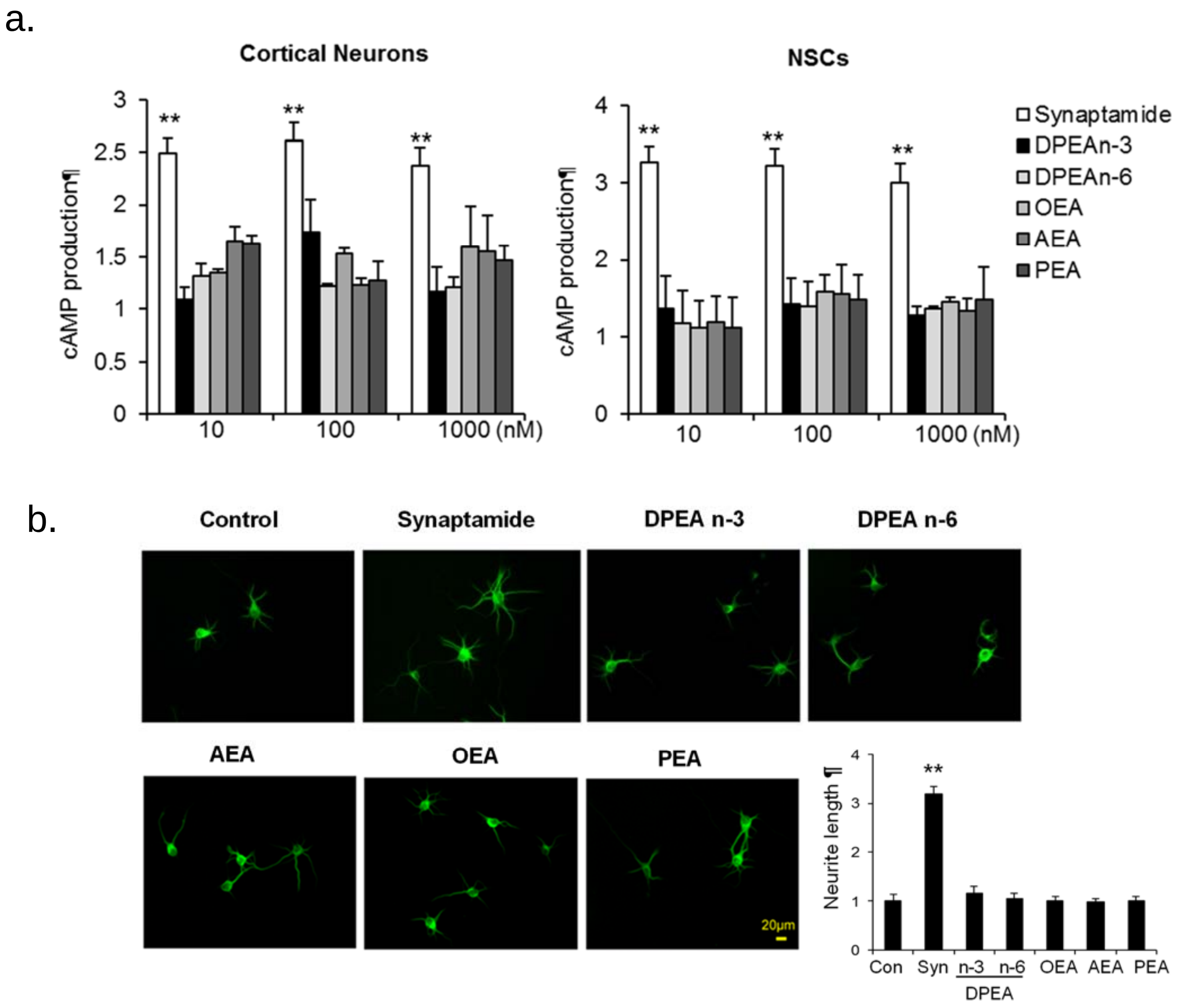

Supplementary Fig. 12. Induction of cAMP production and neurite growth specifically by synaptamide. a. Only synaptamide among various fatty acyl ethanolamides significantly increased cAMP at concentrations up to 1 µM. For cAMP production, DIV3 cortical neurons or NSCs were stimulated with fatty acyl ethanolamides for 10 min. **b.** Representative photomicrographs of MAP-2 positive neurons were obtained after treating DIV1 cortical cultures with 10 nM *N*-acylethanolamines for 48 h, showing induction of neurite outgrowth only by synaptamide. Data are

presented as means ± s.e.m. of biological triplicates, representing three independent experiments. **p<0.01 vs. control (unpaired Student *t*-test). ¶, relative to control.

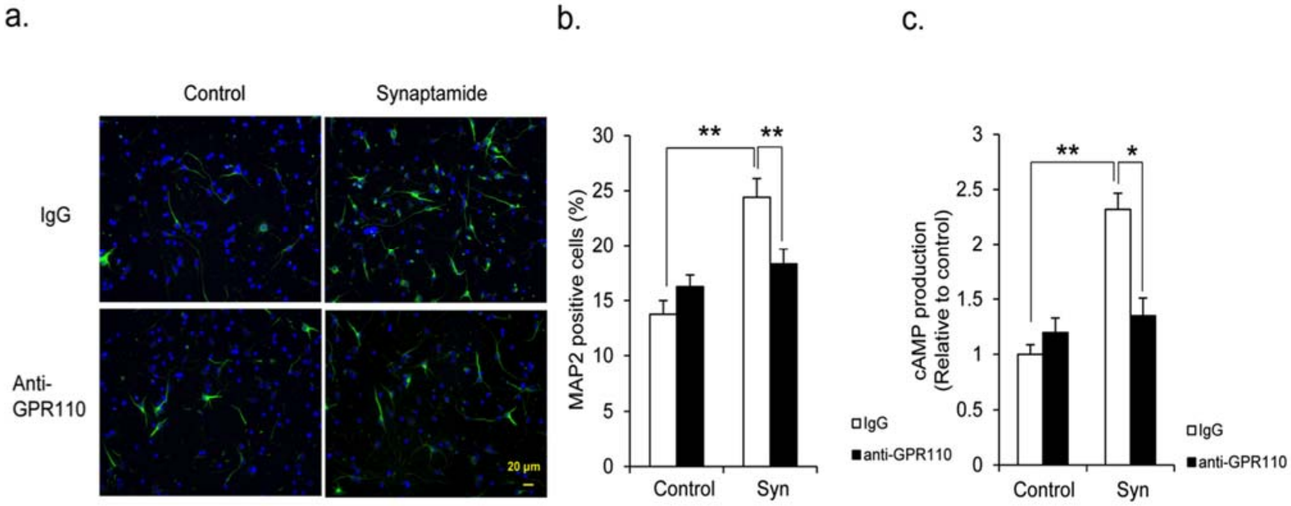

Supplementary Fig. 13. Synaptamide-induced cAMP production and neurogenic differentiation blocked by pretreatment with anti-GPR110 antibody. Mouse NSC cells were pretreated with 0.4 μg/mL *N*-terminal targeting GPR110 antibody for 30 min under a non-permeable condition at 6 h after seeding and subsequently treated with 10 nM synaptamide for 3 days for the evaluation of neurogenic differentiation. Similarly, cAMP production was evaluated after stimulating DIV3 NSCs with synaptamide for 10 min after pretreatment with GPR110 antibody for 30 min. Synaptamide-induced neurogenic differentiation (a, b) and cAMP production (c) were completely blocked by the treatment with GPR110 antibody. Data are presented as means ± s.e.m. of biological triplicates, representing three independent experiments. *p<0.05, **p<0.01 vs. control (unpaired Student *t*-test). Scale bar: 20 μm.

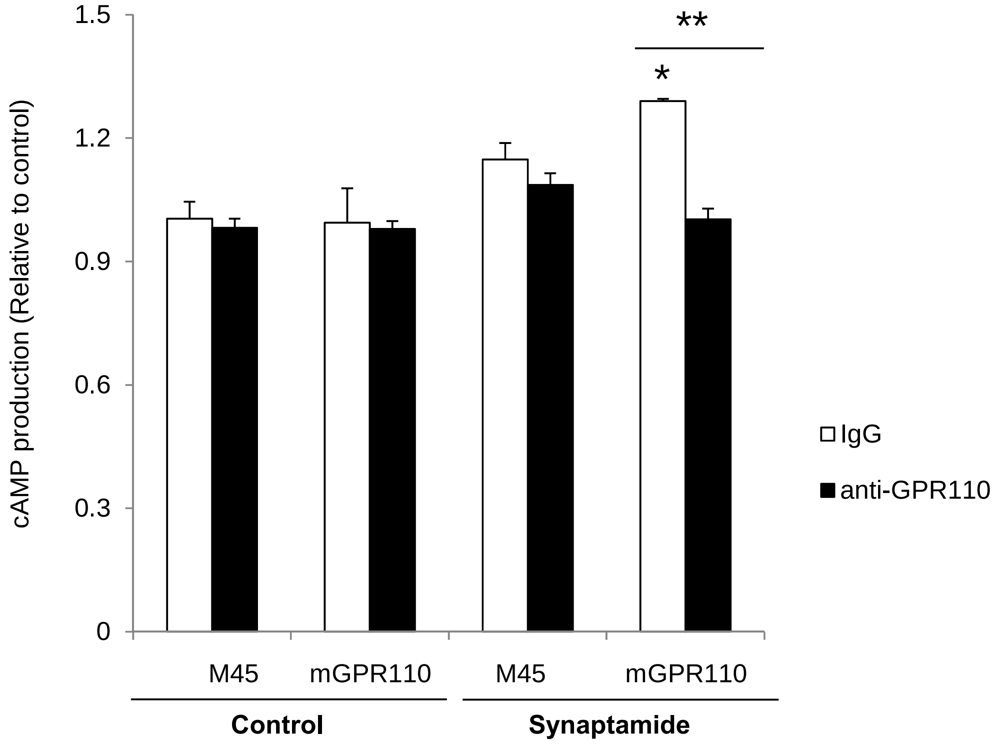

Supplementary Fig. 14. Prevention of synaptamide-induced cAMP production by GPR110 antibody. A549 cells were transfected with empty vector (M45) or HA-tagged mouse GPR110 (mGPR110-HA). After 24 h, cells were treated with 0.4 µg/mL *N*-terminal targeting GPR110 antibody or control IgG for 30 min followed by 10 nM synaptamide for 10 min. Although the increase in cAMP in mGPR110-transfected A549 cells is relatively small in response to 10 nM synaptamide, the increase is still abrogated by anti-GPR110. Data are presented as means ± s.e.m. of biological triplicates, representing three independent experiments. *p<0.05, **p<0.01 vs. control (unpaired Student *t*-test).

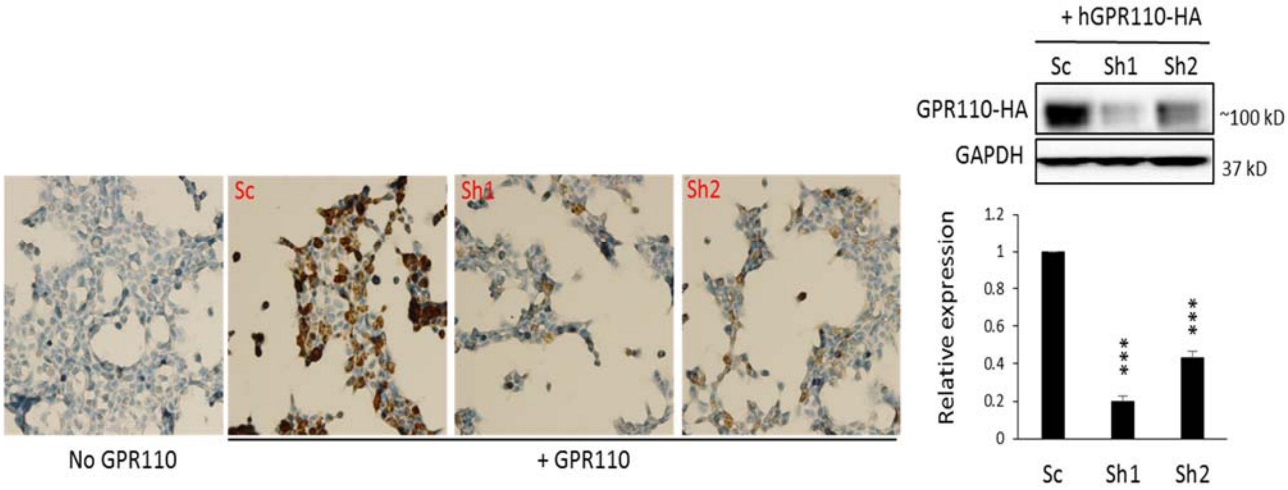

Supplementary Fig. 15. ***In situ* hybridization detecting *gpr*110 overexpression and silencing.** HEK293 cells were co-transfected with mGPR110-HA and GPR110 shRNAs (sh1 and sh2) or scrambled RNA (Sc). Cells were hybridized with the *gpr*110 RNAscope probe. The overexpressed *gpr*110 signal detected by *in situ* hybridization is shown in brown. GPR110 shRNA expression prevented *gpr*110 expression while scrambled control RNA (Sc) showed no effects. Quatitative data are presented as means ± s.e.m. for biological triplicate, representing three independent experiments. ***$p<0.001$ vs. control (unpaired Student *t*-test).

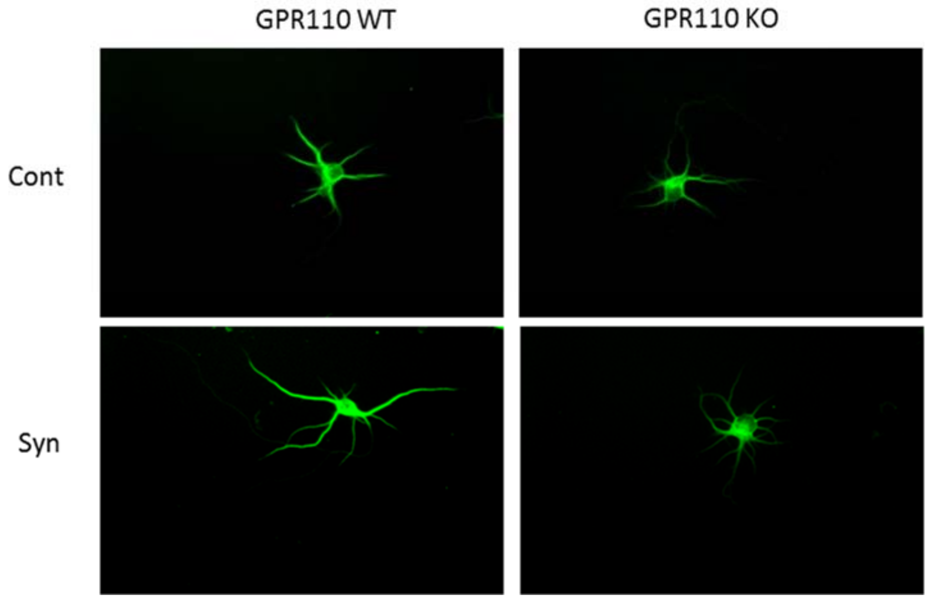

Supplementary Fig. 16. Representative micrographs showing no synaptamide effect on neurite outgrowth in cortical neurons from GPR110 KO mice. The DIV1 cortical neurons obtainbed from GPR110 WT and KO P0 animals were treated with 10 nM synaptamide for 48 h. Synaptamide (Syn) increased neurite growth in WT but not in KO neurons. Representive micrographs are presented from three independent experiments.

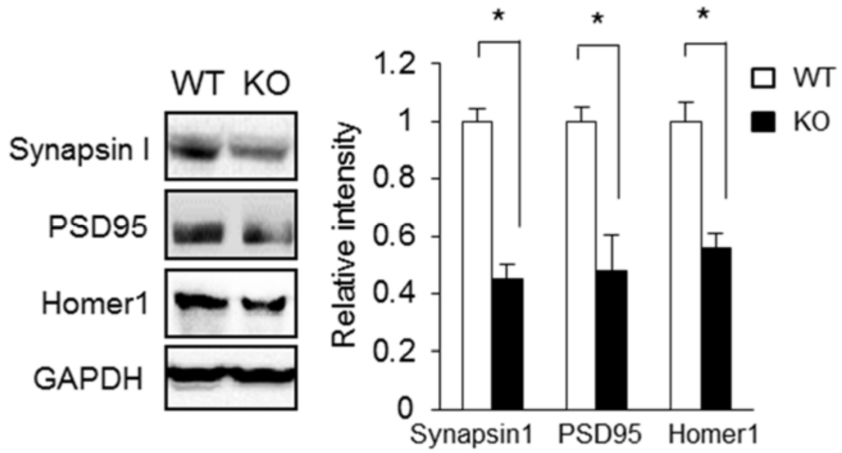

Supplementary Fig. 17. Western blot analysis showing significant decreases in synapsin1, PSD95 and Homer1 proteins in the KO brain. The cortical expression of synapsin1, PSD95 and homer1 in P10 KO brains was significantly lower compared to WT. Data are presented as means ± s.e.m. of biological triplicates, representing two independent experiments. *p<0.05 vs. control (unpaired Student *t*-test).

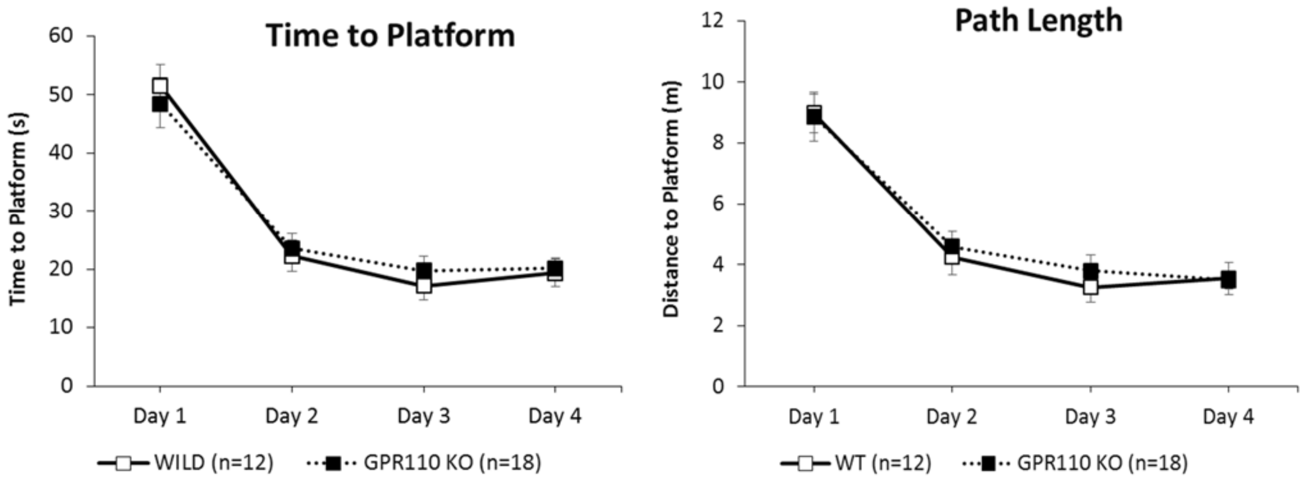

Supplementary Fig. 18. Acquisition trials for Morris water maze. The performance of WT and GPR110 KO mice improved at a similar rate during the training sessions across 4 days. Both the escape time and the distance to platform is comparable between the two groups ($p>0.05$, Two way repeated measures ANOVA). Data are expressed as means ± s.e.m. with the animal numbers as indicated in the figure legend.

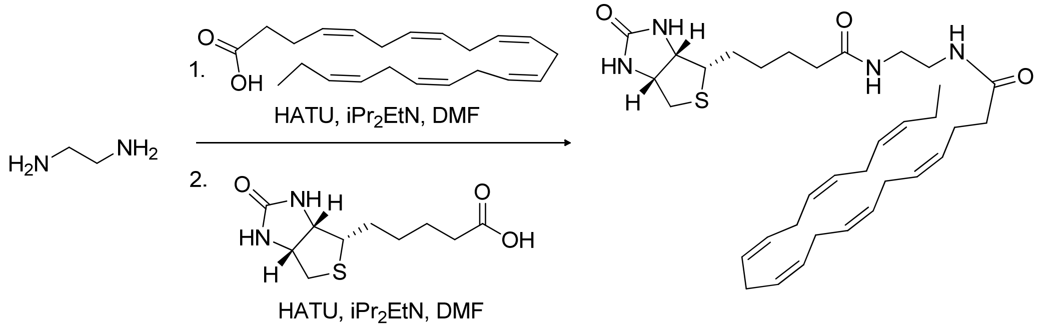

G1

Supplementary Fig. 19. Chemical synthesis of (4*Z*,7*Z*,10*Z*,13*Z*,16*Z*,19*Z*)-*N*-(2-(5-((3a*S*,4*S*,6a*R*)-2-oxohexahydro-1*H*-thieno[3,4-*d*]imidazol-4-yl)pentanamido)ethyl)docosa-4,7,10,13,16,19-hexaenamide (G1).

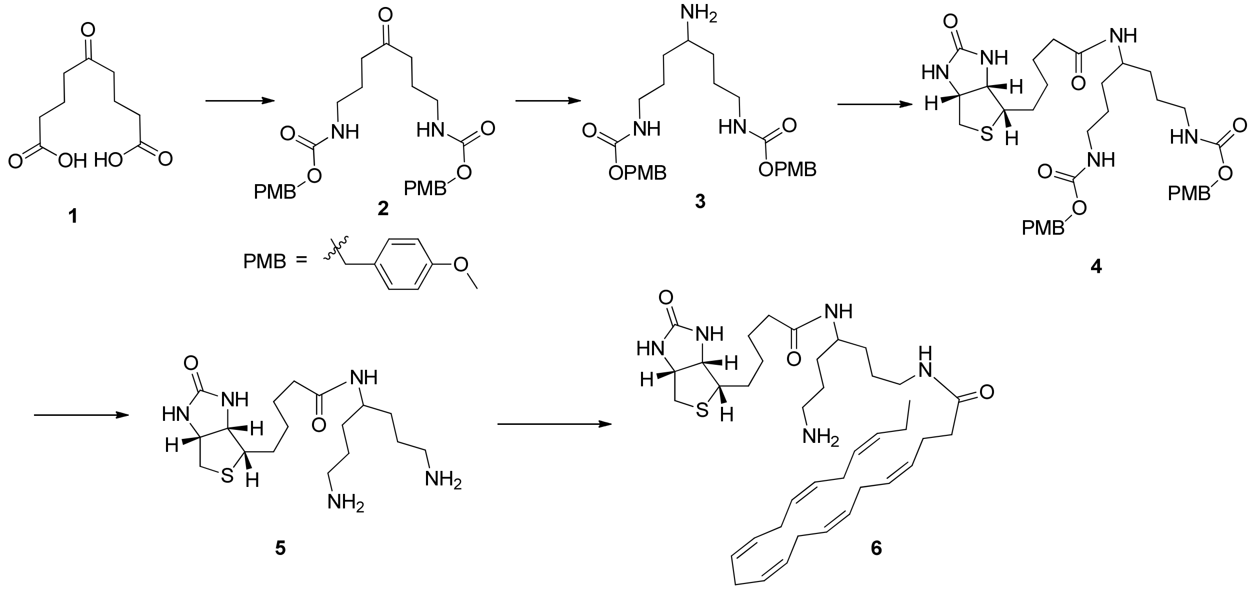

Supplementary Fig. 20. Preparation of (4*Z*,7*Z*,10*Z*,13*Z*,16*Z*,19*Z*)-*N*-(7-amino-4-(5-((3a*S*,4*S*,6a*R*)-2-oxohexahydro-1*H*-thieno[3,4-*d*]imidazol-4-yl)pentanamido)heptyl)docosa-4,7,10,13,16,19-hexaenamide (G1*).

(1) 5-Oxononanedioic acid

(2) 4-Methoxybenzyl 4-oxoheptane-1,7-diyldicarbamate

(3) Bis(4-methoxybenzyl) (4-aminoheptane-1,7-diyl)dicarbamate

(4) Bis(4-methoxybenzyl) (4-(5-((3a*S*,4S,6a*R*)-2-oxohexahydro-1*H*-thieno[3,4-*d*]imidazol-4yl)pentanamido) heptane-1,7-diyl)dicarbamate

(5) *N*-(1,7-diaminoheptan-4-yl)-5-((3a*S*,4*S*,6a*R*)-2-oxohexahydro-1*H*-thieno[3,4-*d*]imidazol-4-yl)pentanamide

(6) (4Z,7Z,10Z,13Z,16Z,19*Z*)-*N*-(7-amino-4-(5-((3a*S*,4*S*,6a*R*)-2-oxohexahydro-1*H*-thieno[3,4-*d*]imidazol-4-yl)pentanamido)heptyl)docosa-4,7,10,13,16,19-hexaenamide (6, G1*).

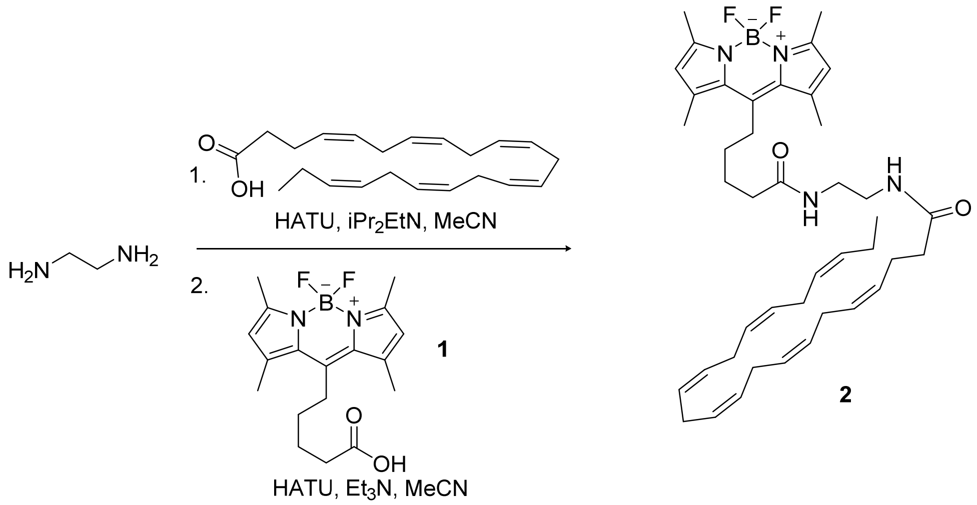

Supplementary Fig. 21. Preparation of 10-(5-((2-((4*Z*,7*Z*,10*Z*,13*Z*,16*Z*,19*Z*)-docosa-4,7,10,13,16,19-hexaenamido)ethyl)amino)-5-oxopentyl)-5,5-difluoro-1,3,7,9-tetramethyl-5*H*-dipyrrolo[1,2-c:2',1'-f][1,3,2]diazaborinin-4-ium-5-uide (Bodipy-synaptamide).

1. 5-(5,5-difluoro-1,3,7,9-tetramethyl-5*H*-$4l^4$,$5l^4$-dipyrrolo[1,2-*c*:2',1'-*f*][1,3,2]diazaborinin-10-yl)pentanoic acid
2. Bodipy-synaptamide

Supplementary Fig. 22. Uncropped western blots

Fig. 2e.

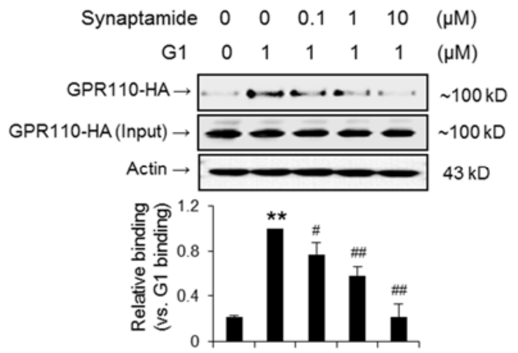

Fig. 2f.

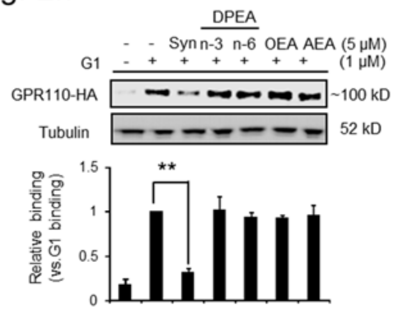

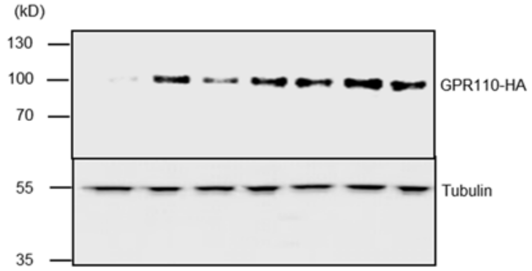

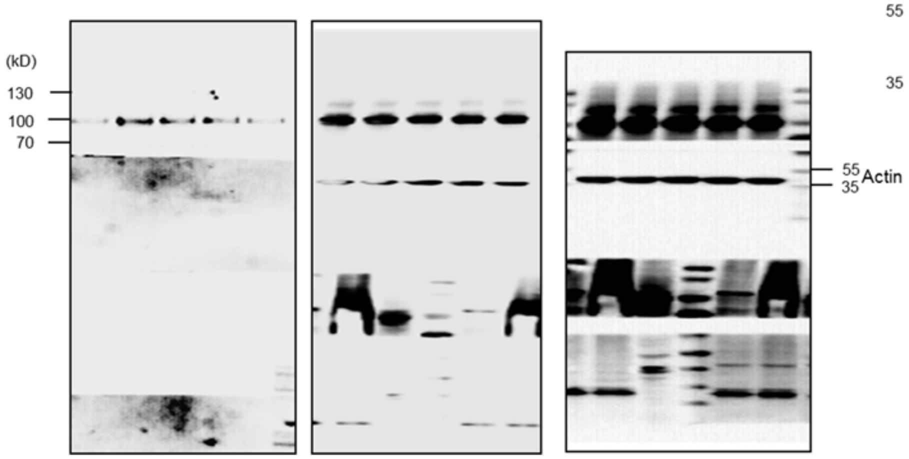

Fig 2h.

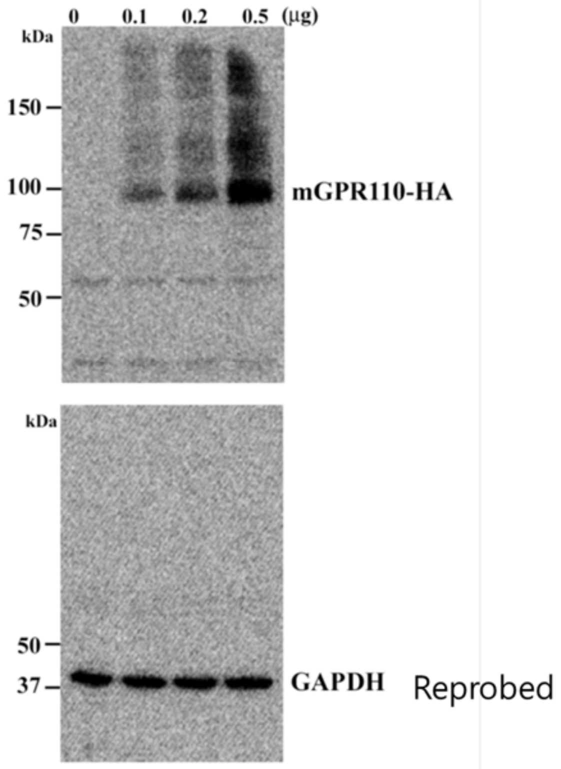
0
0.1
0.2
0.5 (μg)
kDa
150
100
75
50
mGPR110-HA
kDa
50
37
GAPDH
Reprobed

Fig. 2i.

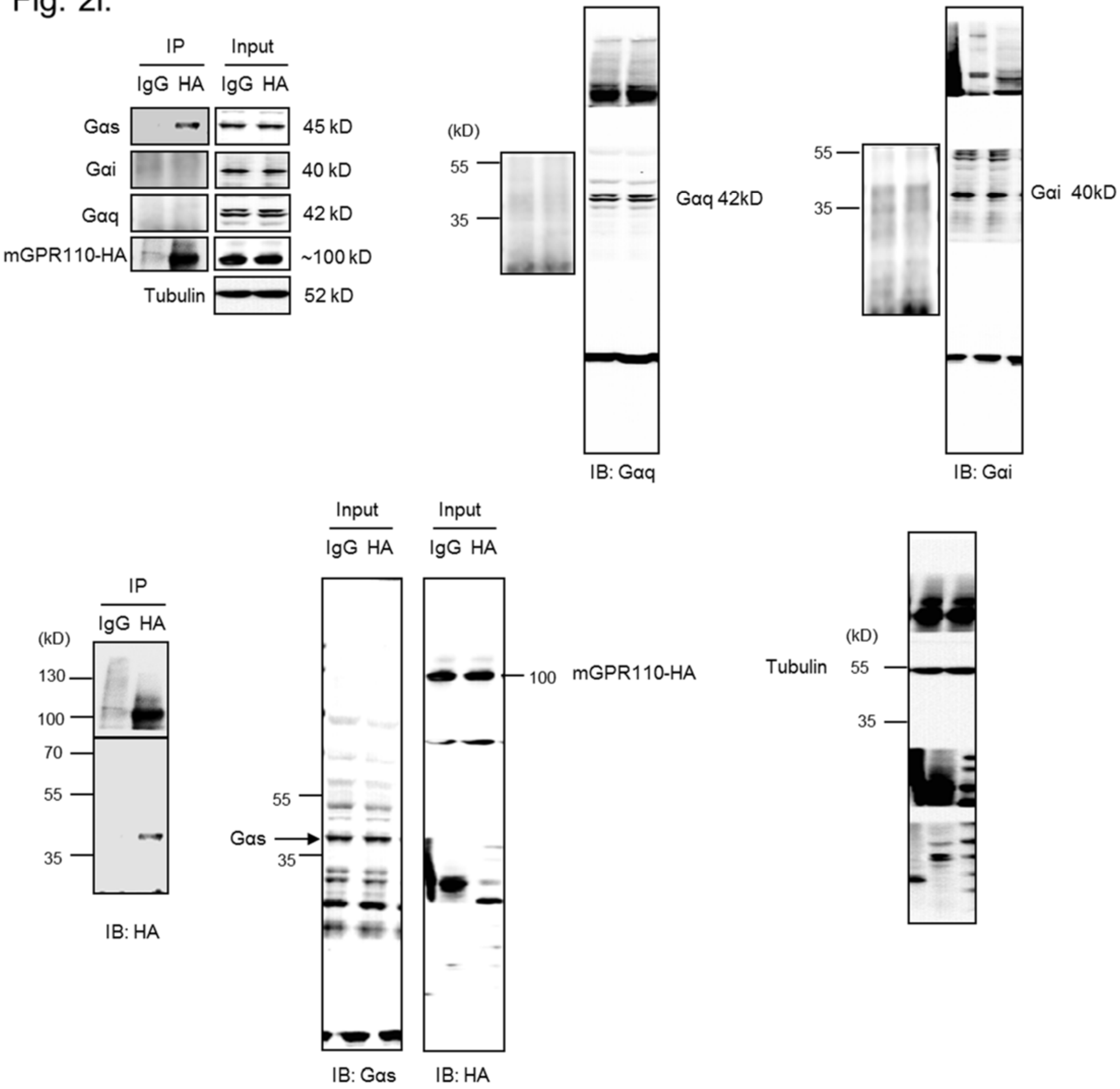
IP
Input
IgG HA IgG HA
Gαs
Gαi
Gαq
mGPR110-HA
Tubulin
45 kD
40 kD
42 kD
~100 kD
52 kD
(kD)
55
35
Gαq 42kD
IB: Gαq
55
35
Gαi 40kD
IB: Gαi
IP
IgG HA
(kD)
130
100
70
55
35
IB: HA
Input
IgG HA
Input
IgG HA
55
Gαs
35
100
mGPR110-HA
IB: Gαs
IB: HA
(kD)
Tubulin
55
35

Fig. 3d.

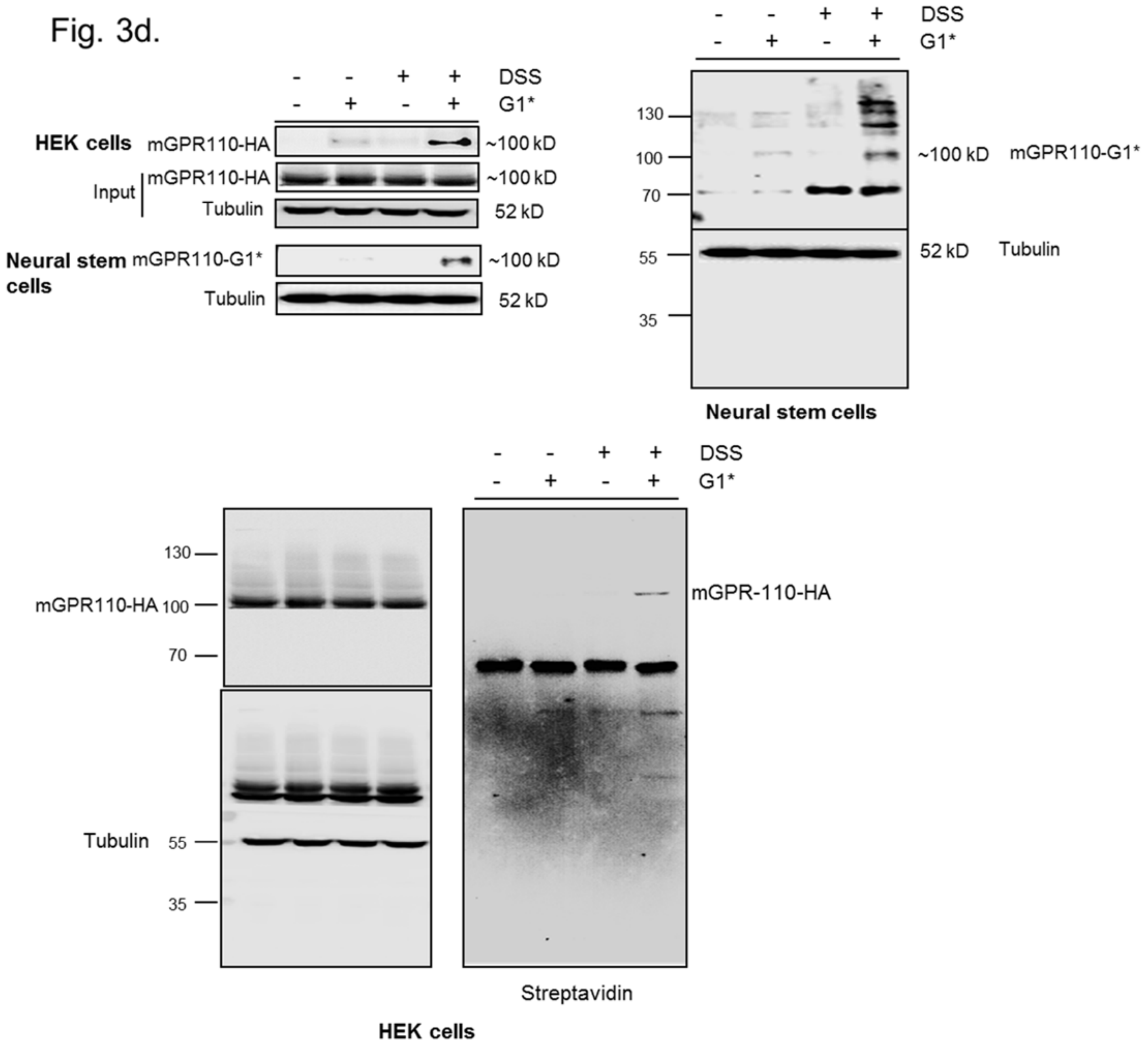
DSS
G1*
HEK cells
mGPR110-HA
~100 kD
Input
mGPR110-HA
~100 kD
Tubulin
52 kD
Neural stem cells
mGPR110-G1*
~100 kD
Tubulin
52 kD
130
100
70
55
35
~100 kD
mGPR110-G1*
52 kD
Tubulin
Neural stem cells
mGPR110-HA
Tubulin
mGPR-110-HA
Streptavidin
HEK cells

Fig. 4b.

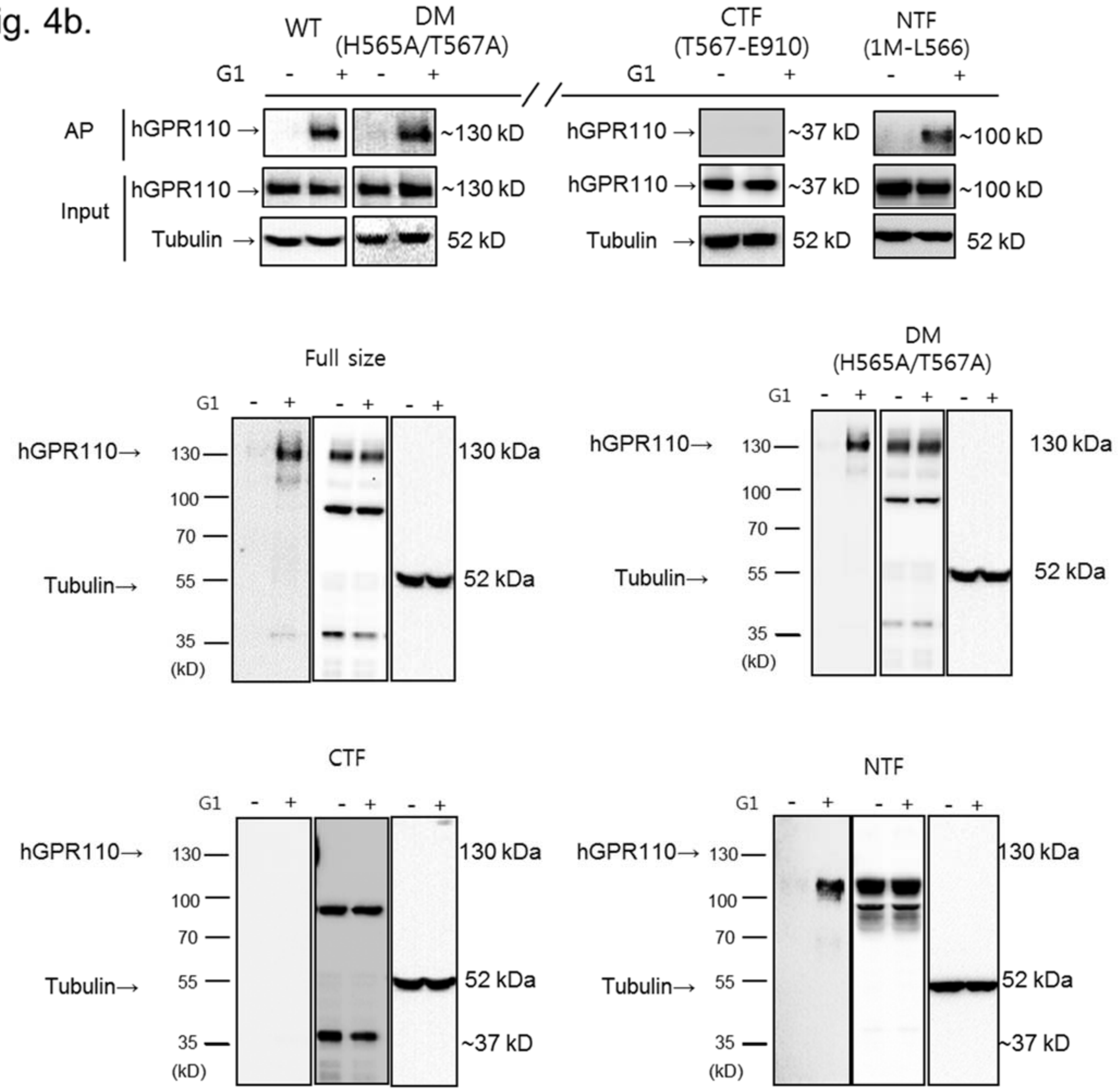

WT
DM (H565A/T567A)
CTF (T567-E910)
NTF (1M-L566)
G1
AP
Input
hGPR110
Tubulin
~130 kD
~37 kD
~100 kD
52 kD
Full size
DM (H565A/T567A)
CTF
NTF
130 kDa
52 kDa
~37 kD
130
100
70
55
35
(kD)

Fig.7a.

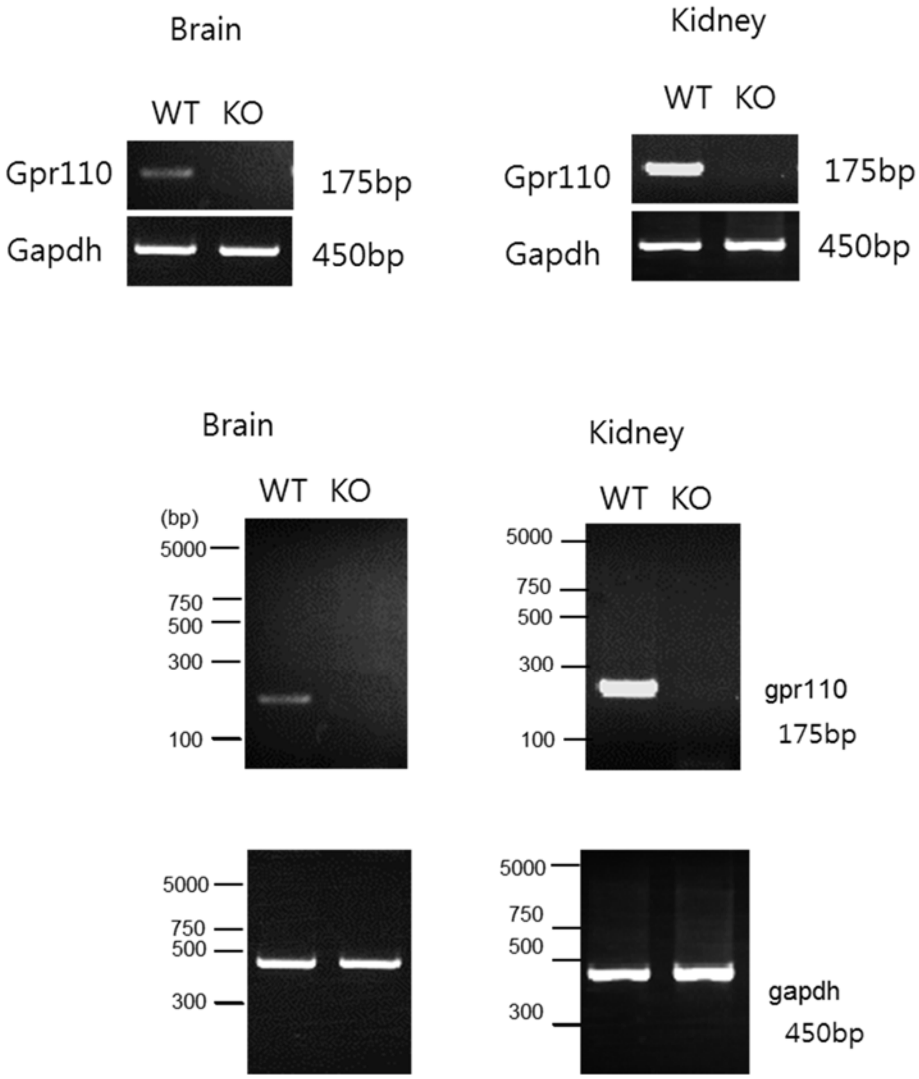
Brain
WT KO
Gpr110
175bp
Gapdh
450bp
Kidney
WT KO
Gpr110
175bp
Gapdh
450bp
Brain
WT KO
(bp)
5000
750
500
300
100
Kidney
WT KO
5000
750
500
300
100
gpr110
175bp
5000
750
500
300
5000
750
500
300
gapdh
450bp

Fig. 5e.

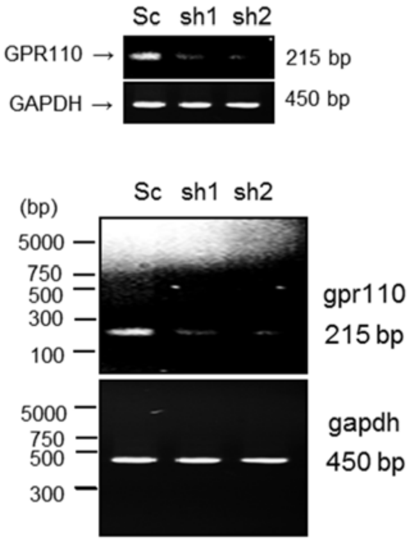
Sc sh1 sh2
GPR110 →
215 bp
GAPDH →
450 bp
Sc sh1 sh2
(bp)
5000
750
500
300
100
gpr110
215 bp
5000
750
500
300
gapdh
450 bp

Fig. 5i.

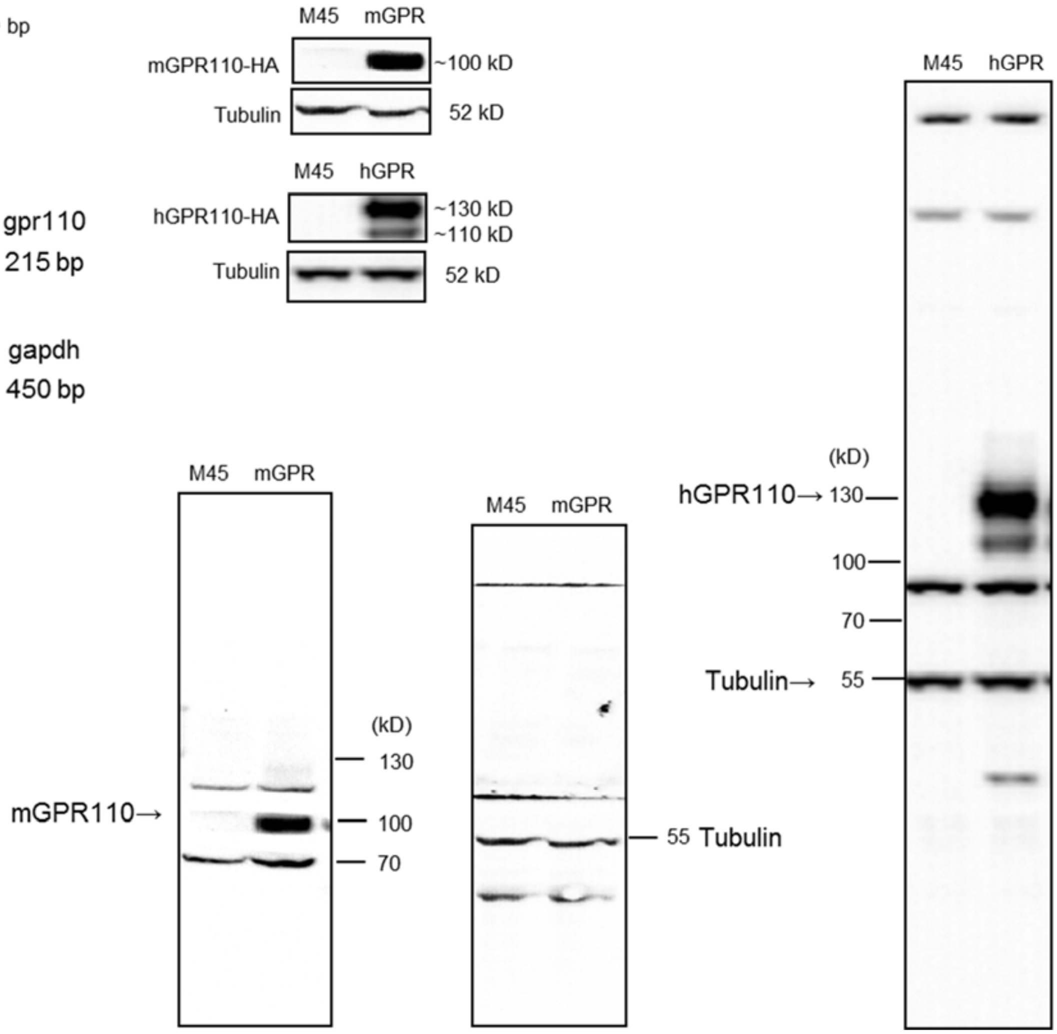
M45 mGPR
mGPR110-HA
~100 kD
Tubulin
52 kD
M45 hGPR
hGPR110-HA
~130 kD
~110 kD
Tubulin
52 kD
M45 hGPR
(kD)
hGPR110→ 130
100
70
Tubulin→ 55
M45 mGPR
(kD)
130
mGPR110→
100
70
M45 mGPR
55 Tubulin

Fig. S3

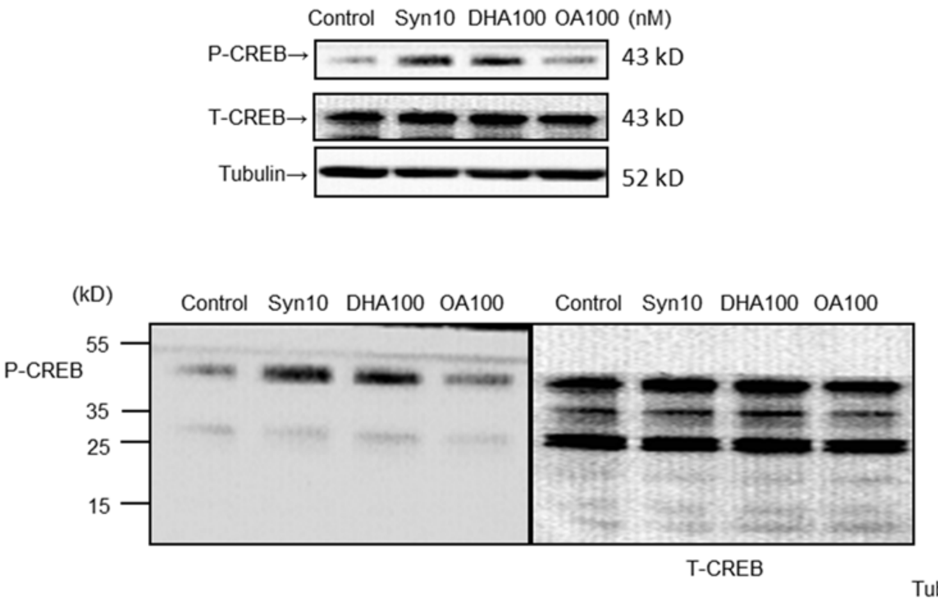
Control Syn10 DHA100 OA100 (nM)
P-CREB→ 43 kD
T-CREB→ 43 kD
Tubulin→ 52 kD
(kD)
Control Syn10 DHA100 OA100 Control Syn10 DHA100 OA100
55
P-CREB
35
25
15
T-CREB

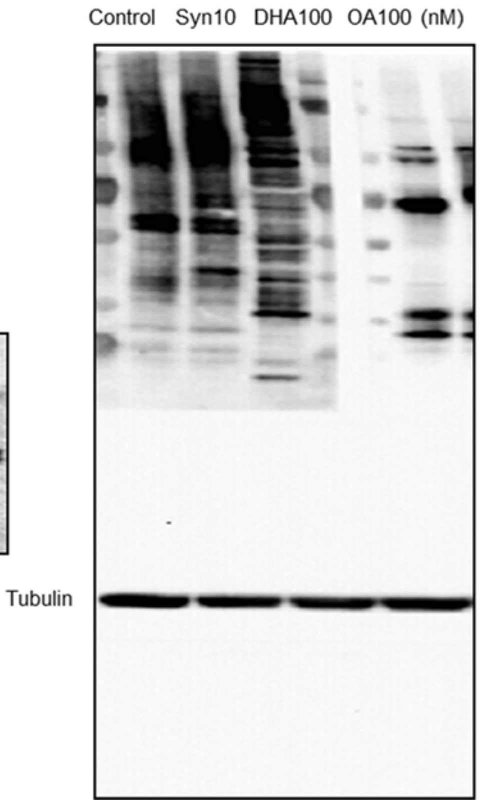
Control Syn10 DHA100 OA100 (nM)
Tubulin

Fig. S6

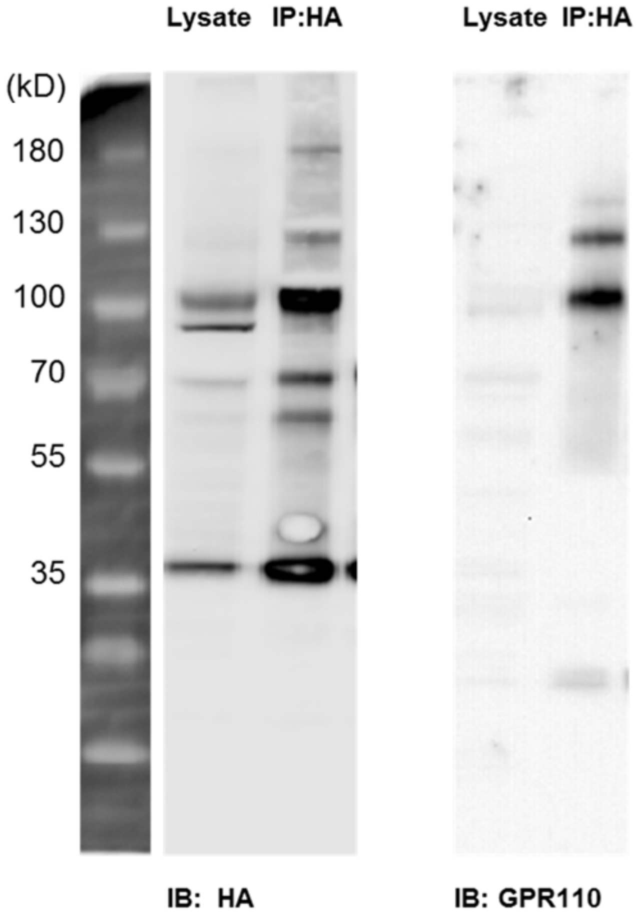

Fig. S7a

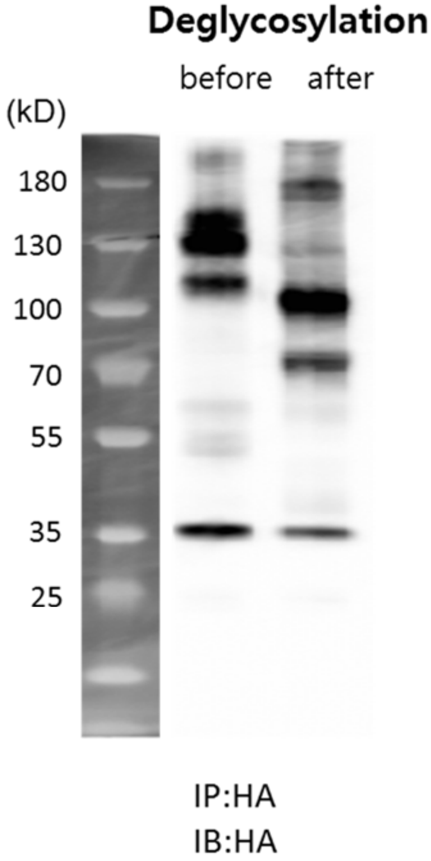

Fig. S10

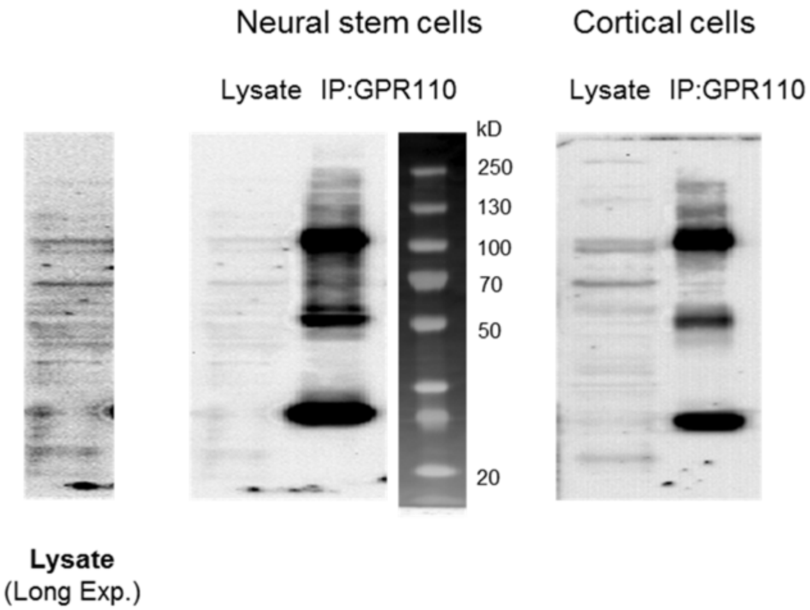

Neural stem cells
Cortical cells
Lysate
IP:GPR110
Lysate
IP:GPR110
kD
250
130
100
70
50
20
Lysate
(Long Exp.)

Fig. S17

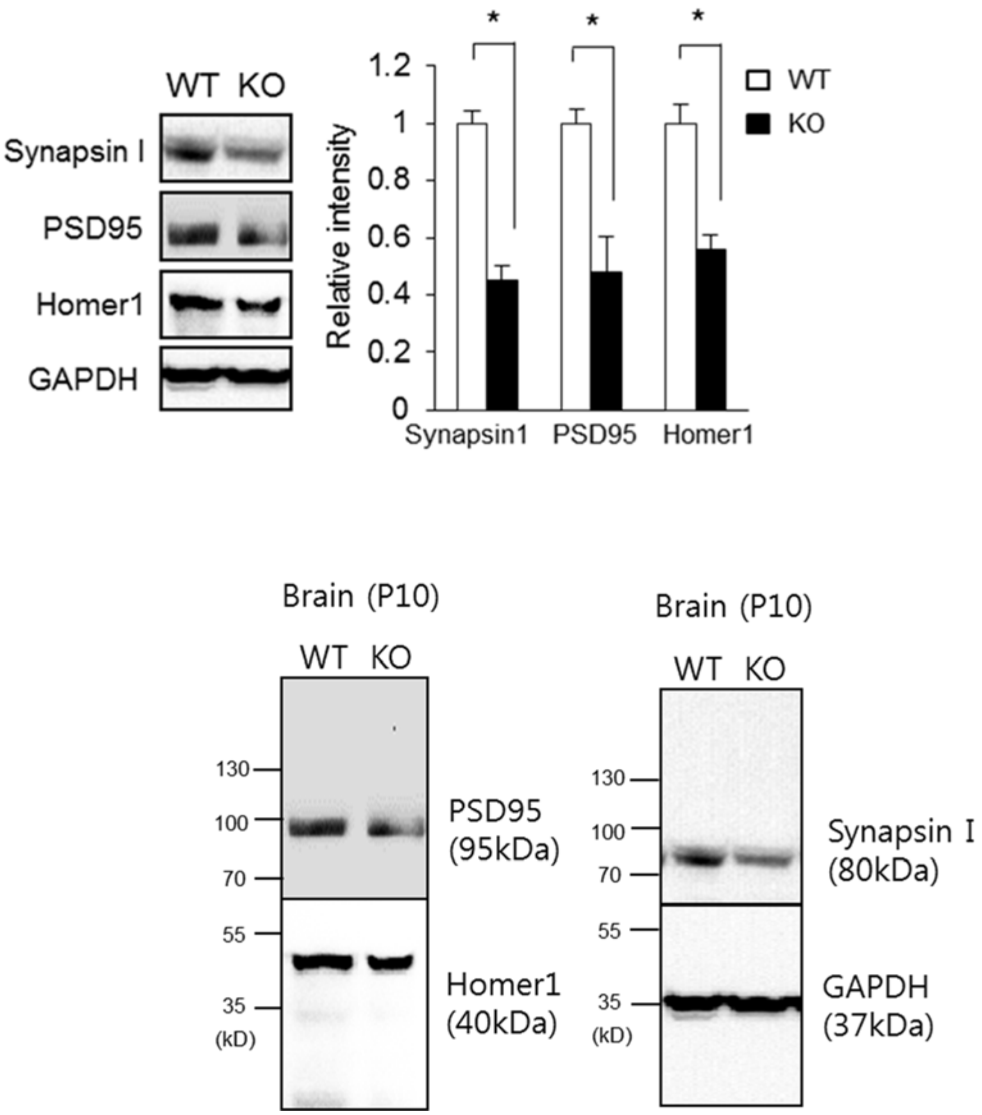
WT KO
Synapsin I
PSD95
Homer1
GAPDH
Relative intensity
1.2
1
0.8
0.6
0.4
0.2
0
*
*
*
WT
KO
Synapsin1
PSD95
Homer1
Brain (P10)
WT KO
130
100
70
55
35
(kD)
PSD95
(95kDa)
Homer1
(40kDa)
Brain (P10)
WT KO
130
100
70
55
35
(kD)
Synapsin I
(80kDa)
GAPDH
(37kDa)

Fig. S15

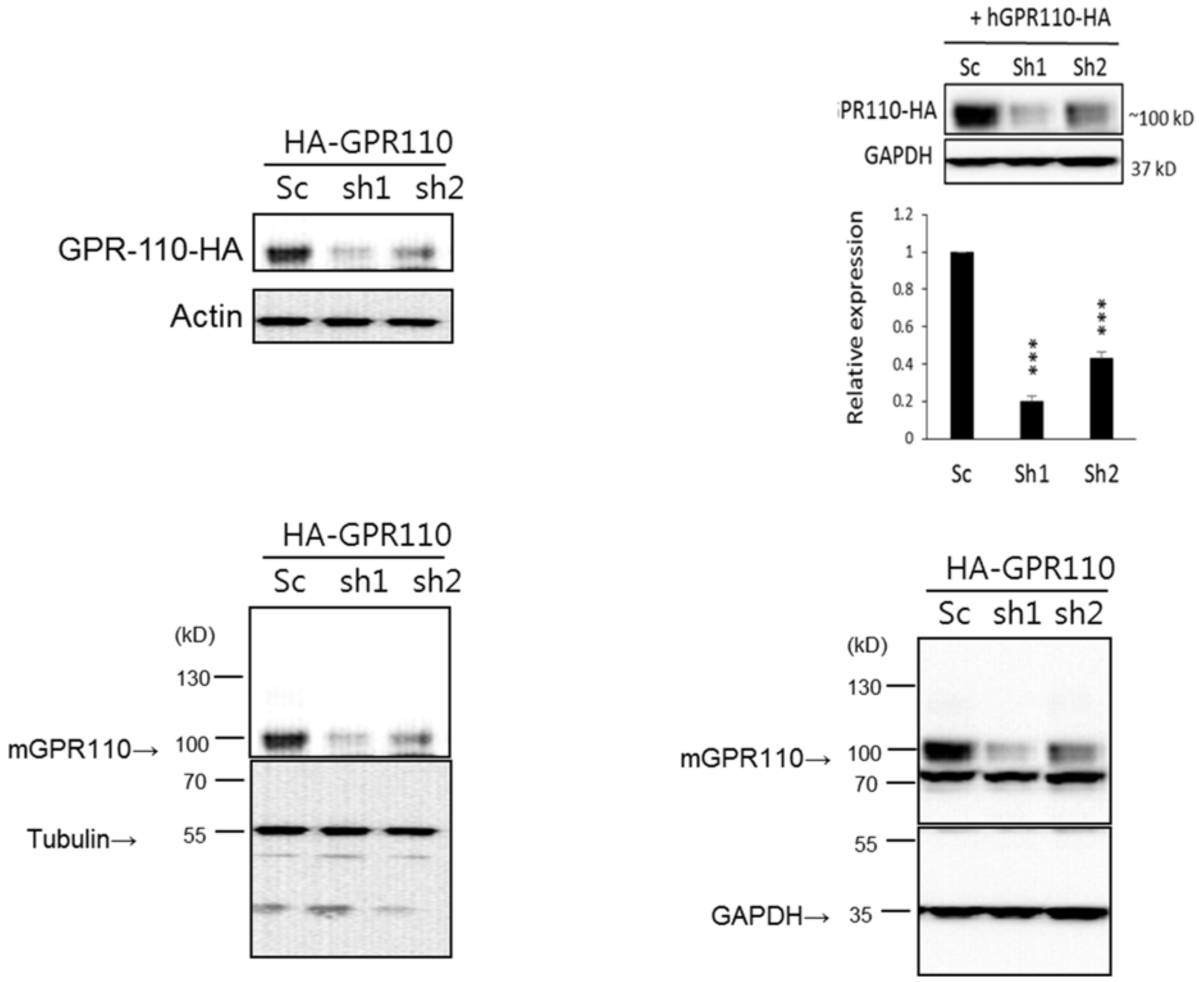
HA-GPR110
Sc sh1 sh2
GPR-110-HA
Actin
+ hGPR110-HA
Sc Sh1 Sh2
PR110-HA
~100 kD
GAPDH
37 kD
Relative expression
1.2
1
0.8
0.6
0.4
0.2
0
***
***
Sc Sh1 Sh2
HA-GPR110
Sc sh1 sh2
(kD)
130
100
70
55
mGPR110→
Tubulin→
HA-GPR110
Sc sh1 sh2
(kD)
130
100
70
55
35
mGPR110→
GAPDH→

Supplementary Table 1. Unique GPR110 peptides identified from fetal brain after affinity purification, SDSPAGE, in-gel digestion and mass spectrometric analysis

prot_acc #	prot_desc	prot_ Score	prot_ Mass	prot_ Match _sig	pep_ Rank	pep_ Is_ bold	pep_ Is_ unique	pep_exp_ m/z	pep_exp_ Mr	pep_ exp_z	pep_calc_ Mr	pep_error	pep_ Score	pep_ Expect	pep_res _before	pep_seq	pep_res _after
gi|17512539	G protein-coupled receptor 110	86	102829	3	1	1	1	594.3187	1186.623	2	1186.631	-0.0077	62.97	2.80E-06	K	GTPVTQIQSTR	G
gi|17512539	G protein-coupled receptor 110	86	102829	3	1	1	1	722.8595	1443.705	2	1443.715	-0.0102	40.49	0.00027	R	GHVFIEPDQFQK	S

Supplementary Table 2. Unique peptides of mGPR110 (gi/17512539) overexpressed in HEK cells identified from ~ 100 kD band (band 2 in supplementary Fig. 6) by mass spectrometry

pep_exp_m/z	pep_exp_mr	exp_z	pep_calc_mr	pep_error	pep_score	pep_expect	pep_sequence	pep_var_mod
468.2635	934.5124	2	934.5124	0	32.04	0.00099	K.LTTLSSWK.Q	
514.7851	1027.5556	2	1027.556	-0.0007	33.46	0.0018	R.TFNILQHR.G	
546.2733	1090.5321	2	1090.533	-0.0007	31.04	0.012	K.EIVSMVEER.H	
594.322	1186.6295	2	1186.631	-0.0011	33.27	0.005	K.GTPVTQIQSTR.G	
596.2494	1190.4842	2	1190.485	-0.0006	47.46	3.50E-05	R.GYNYQMEMR.Q	
611.7843	1221.554	2	1221.556	-0.0021	50.25	0.00032	R.CQSSGWQITR.E	
612.2443	1222.4741	2	1222.475	-0.0006	44.97	6.60E-05	R.GYNYQMEMR.Q	2 Oxidation (M)
717.8599	1433.7053	2	1433.707	-0.0019	51.79	0.00045	R.ESCVLSQLEELK.K	
718.8158	1435.6171	2	1435.619	-0.0019	75.95	8.20E-08	R.NDVCWLNWSDK.S	
722.8641	1443.7137	2	1443.715	-0.0009	45.42	0.00033	R.GHVFIEPDQFQK.S	
781.9081	1561.8017	2	1561.802	-0.0004	44.1	0.0019	R.ESCVLSQLEELKK.E	
796.866	1591.7175	2	1591.72	-0.0026	67.54	1.40E-06	K.RNDVCWLNWSDK.S	
626.3351	1875.9835	3	1875.984	-0.0008	38.95	0.0018	K.FIDWKGTPVTQIQSTR.G	
628.3323	1881.9749	3	1881.974	0.0012	52.61	8.50E-05	R.HPVHEYEVLLQVTYR.D	
1047.0707	2092.1268	2	2092.124	0.003	75.33	1.00E-07	K.TIISMASLTFGDILPITQR.G	Oxidation (M)
1111.0948	2220.175	2	2220.171	0.0039	38.81	0.0025	K.TLESISSLIPSMALPLNFSGK.F	Oxidation (M)
621.0648	2480.2303	4	2480.234	-0.0033	35.8	0.0047	R.HPVHEYEVLLQVTYRDPEEK.R	
848.1269	2541.359	3	2541.363	-0.0035	21.29	0.011	K.SHPKTIISMASLTFGDILPITQR.G	Oxidation (M)
1334.6648	2667.315	2	2667.311	0.004	63.52	1.10E-06	R.CSHLTSFSMLMSPFVPSSVVPVVK.W	2 Oxidation (M)
992.7772	3967.0799	4	3967.067	0.0133	17.92	0.045	R.GHVFIEPDQFQKSHPKTIISMASLTFGDILPITQR.G	Oxidation (M)

Supplementary Table 3. Unique peptides of mGPR110 (gi/17512539) overexpressed in HEK cells identified from ~130 kD band (band 1 in supplementary Fig. 6) by mass spectrometry

pep_exp_m/z	pep_exp_mr	exp_z	pep_calc_mr	pep_error	pep_score	pep_expect	pep_sequence	pep_var_mod
468.2631	934.5117	2	934.5124	-0.0007	25.78	0.0038	K.LTTLSSWK.Q	
546.273	1090.531	2	1090.533	-0.0014	34.07	0.0088	K.EIVSMVEER.H	
594.3225	1186.63	2	1186.631	-0.0002	37.26	0.0013	K.GTPVTQIQSTR.G	
596.2491	1190.484	2	1190.485	-0.0013	44.36	6.90E-05	R.GYNYQMEMR.Q	
718.8159	1435.617	2	1435.619	-0.0018	43.11	9.10E-05	R.NDVCWLNWSDK.S	
722.863	1443.711	2	1443.715	-0.0033	31.83	0.001	R.GHVFIEPDQFQK.S	
781.9067	1561.799	2	1561.802	-0.0033	36.1	0.0014	R.ESCVLSQLEELKK.E	
628.3304	1881.97	3	1881.974	-0.0042	18.51	0.018	R.HPVHEYEVLLQVTYR.D	
1047.068	2092.121	2	2092.124	-0.0032	55.79	6.10E-06	K.TIISMASLTFGDILPITQR.G	Oxidation (M)
1111.091	2220.167	2	2220.171	-0.0039	23.82	0.007	K.TLESISSLIPSMALPLNFSGK.F	Oxidation (M)

Supplementary Table 4. Unique hGPR110 (gi/61743940) peptides identified by mass spectrometry from ~ 130 kD band (band 1 in supplementary Fig. 7)

pep_exp_m/z	pep_exp_mr	exp_z	pep_calc_mr	pep_error	pep_score	pep_expect	pep_sequence	pep_var_mod
376.2103	750.4061	2	750.4065	-0.0004	17.85	0.032	K.IWGTFK.I	
446.2498	890.485	2	890.4861	-0.0011	37.41	0.0026	K.LSALSSWK.Q	
327.523	979.5471	3	979.5491	-0.002	21.58	0.022	R.TKIWGTFK.I	
574.8182	1147.622	2	1147.624	-0.0018	44.47	0.0054	K.YANGIEIQLK.K	
581.8138	1161.613	2	1161.614	-0.0012	47.28	0.0012	R.VLIGSDQFQR.S	
590.8023	1179.59	2	1179.592	-0.0024	43.41	0.001	K.LFPLEDGSFR.V	
611.2861	1220.558	2	1220.561	-0.0032	66.39	1.90E-05	K.CESSGWQVIR.E	
426.2464	1275.718	3	1275.719	-0.0012	30.79	0.0083	K.YANGIEIQLKK.A	
660.3576	1318.701	2	1318.703	-0.0028	25.11	0.014	K.FSKPFNPLQNK.G	
721.8376	1441.661	2	1441.666	-0.0054	83.57	1.50E-08	K.AQCNDIVFGFGSK.D	
769.8999	1537.785	2	1537.789	-0.0036	66.08	2.20E-06	R.IQGFESVQVTQFR.N	
774.4026	1546.791	2	1546.791	-0.0005	80.87	1.20E-06	R.ETCVLSLLEELNK.N	
781.8246	1561.635	2	1561.636	-0.0009	44.59	6.60E-05	K.DDEYTLPCSSGYR.G	
817.9656	1633.917	2	1633.919	-0.0025	81.69	2.20E-08	R.QLLFNKLSALSSWK.Q	
548.3423	1642.005	3	1642.008	-0.003	49.32	2.40E-05	K.LLKPPLLWSHGLIR.I	
577.644	1729.91	3	1729.915	-0.005	45.43	7.90E-05	K.TALHKLFPLEDGSFR.V	
1029.553	2057.092	2	2057.095	-0.0025	114.13	2.00E-11	K.HLGPVEEYQLLLQVTYR.D	
1093.603	2185.191	2	2185.19	0.0011	100.29	4.00E-10	K.KHLGPVEEYQLLLQVTYR.D	
1150.642	2299.268	2	2299.271	-0.0024	48.42	2.90E-05	R.SLPETIISMASLTLGNILPVSK.N	Oxidation (M)
796.755	2387.243	3	2387.249	-0.0054	32.75	0.00099	K.HLGPVEEYQLLLQVTYRDSK.E	
629.842	2515.339	4	2515.343	-0.0045	23.82	0.023	K.KHLGPVEEYQLLLQVTYRDSK.E	
662.1032	2644.384	4	2644.386	-0.0024	27.01	0.0053	K.HLGPVEEYQLLLQVTYRDSKEK.R	
1493.653	2985.292	2	2985.291	0.0013	68.24	4.00E-07	K.AQCNDIVFGFGSKDDEYTLPCSSGYR.G	
1033.885	3098.632	3	3098.636	-0.004	26.01	0.0036	R.QNPSTTVGNLASVVSILSNISSLSLASHFR.V	

Supplementary Table 5. Unique hGPR110 (gi/61743940) peptides identified by mass spectrometry from ~ 110 kD band (band 2 in supplementary Fig. 7)

pep_exp_m/z	pep_exp_mr	exp_z	pep_calc_mr	pep_error	pep_score	pep_expect	pep_sequence	pep_var_mod
446.2498	890.485	2	890.4861	-0.0011	34.21	0.0052	K.LSALSSWK.Q	
557.3103	1112.606	2	1112.609	-0.0031	32.53	0.0043	K.LWRPTVGER.L	
574.8179	1147.621	2	1147.624	-0.0025	58.29	3.30E-05	K.YANGIEIQLK.K	
581.8128	1161.611	2	1161.614	-0.0032	58.79	1.30E-05	R.VLIGSDQFQR.S	
590.8018	1179.589	2	1179.592	-0.0033	35.67	0.0063	K.LFPLEDGSFR.V	
611.2871	1220.56	2	1220.561	-0.0012	55.51	9.40E-05	K.CESSGWQVIR.E	
660.3576	1318.701	2	1318.703	-0.0026	19.48	0.029	K.FSKPFNPLQNK.G	
721.8386	1441.663	2	1441.666	-0.0033	71.33	2.10E-07	K.AQCNDIVFGFGSK.D	
769.9001	1537.786	2	1537.789	-0.0033	70.11	9.70E-07	R.IQGFESVQVTQFR.N	
774.4017	1546.789	2	1546.791	-0.0024	82.89	2.20E-07	R.ETCVLSLLEELNK.N	
781.8235	1561.633	2	1561.636	-0.0029	24.18	0.0054	K.DDEYTLPCSSGYR.G	
822.0115	1642.009	2	1642.008	0.0002	37.2	0.00032	K.LLKPPLLWSHGLIR.I	
830.4144	1658.814	2	1658.815	-0.001	83.61	4.60E-08	R.FTNDLLNSSSAIYSK.Y	
1029.555	2057.096	2	2057.095	0.0013	115.65	1.80E-11	K.HLGPVEEYQLLLQVTYR.D	
1150.643	2299.272	2	2299.271	0.0012	82.72	3.00E-08	R.SLPETIISMASLTLGNILPVSK.N	Oxidation (M)
968.4862	2902.437	3	2902.456	-0.0191	26.81	0.0036	R.NGSIVAGYEVVGSSSASELLSAIEHVAEK.A	
996.1024	2985.285	3	2985.291	-0.0057	47.87	3.20E-05	K.AQCNDIVFGFGSKDDEYTLPCSSGYR.G	
1033.886	3098.636	3	3098.636	-0.0004	42.87	0.00034	R.QNPSTTVGNLASVVSILSNISSLSLASHFR.V	

Supplementary Table 6. Unique hGPR110 (gi/61743940) peptides identified by mass spectrometry from ~ 100 kD band (band 3 in supplementary Fig. 7)

pep_exp_m/z	pep_exp_mr	exp_z	pep_calc_mr	pep_error	pep_score	pep_expect	pep_sequence	pep_var_mod
446.2492	890.4838	2	890.4861	-0.0023	36	0.0024	K.LSALSSWK.Q	
371.8767	1112.608	3	1112.609	-0.0009	26.73	0.019	K.LWRPTVGER.L	
574.8178	1147.621	2	1147.624	-0.0026	50.17	0.00023	K.YANGIEIQLK.K	
581.8127	1161.611	2	1161.614	-0.0033	68.56	2.20E-06	R.VLIGSDQFQR.S	
590.8025	1179.59	2	1179.592	-0.002	62.28	4.80E-05	K.LFPLEDGSFR.V	
611.2854	1220.556	2	1220.561	-0.0046	45.21	0.0018	K.CESSGWQVIR.E	
638.865	1275.716	2	1275.719	-0.0031	49.47	0.00041	K.YANGIEIQLKK.A	
660.3575	1318.7	2	1318.703	-0.003	30.02	0.0049	K.FSKPFNPLQNK.G	
721.8379	1441.661	2	1441.666	-0.0048	59.3	2.80E-06	K.AQCNDIVFGFGSK.D	
769.8985	1537.782	2	1537.789	-0.0066	71.47	1.30E-06	R.IQGFESVQVTQFR.N	
774.4009	1546.787	2	1546.791	-0.004	82.86	5.60E-07	R.ETCVLSLLEELNK.N	
781.8234	1561.632	2	1561.636	-0.0032	55.17	6.70E-06	K.DDEYTLPCSSGYR.G	
548.3421	1642.004	3	1642.008	-0.0038	43.67	8.00E-05	K.LLKPPLLWSHGLIR.I	
830.4126	1658.811	2	1658.815	-0.0044	62.11	3.90E-06	R.FTNDLLNSSSAIYSK.Y	
577.6451	1729.913	3	1729.915	-0.0017	42.07	0.00011	K.TALHKLFPLEDGSFR.V	
1029.553	2057.092	2	2057.095	-0.0025	99.25	6.80E-10	K.HLGPVEEYQLLLQVTYR.D	
729.4029	2185.187	3	2185.19	-0.0028	56.95	5.20E-06	K.KHLGPVEEYQLLLQVTYR.D	
1150.642	2299.27	2	2299.271	-0.0006	64.87	8.30E-07	R.SLPETIISMASLTLGNILPVSK.N	Oxidation (M)
796.7554	2387.244	3	2387.249	-0.0041	46.99	3.90E-05	K.HLGPVEEYQLLLQVTYRDSK.E	
662.103	2644.383	4	2644.386	-0.0033	61.63	6.00E-06	K.HLGPVEEYQLLLQVTYRDSKEK.R	
1493.652	2985.289	2	2985.291	-0.0019	55.71	6.00E-06	K.AQCNDIVFGFGSKDDEYTLPCSSGYR.G	
1033.885	3098.633	3	3098.636	-0.0034	47.53	3.50E-05	R.QNPSTTVGNLASVVSILSNISSLSLASHFR.V	
1148.629	3442.865	3	3442.875	-0.0097	26.16	0.0035	R.VLIGSDQFQRSLPETIISMASLTLGNILPVSK.N	Oxidation (M)
